# ArgR of *Streptomyces coelicolor* Is a Versatile Regulator

**DOI:** 10.1371/journal.pone.0032697

**Published:** 2012-03-05

**Authors:** Rosario Pérez-Redondo, Antonio Rodríguez-García, Alma Botas, Irene Santamarta, Juan F. Martín, Paloma Liras

**Affiliations:** 1 Área de Microbiología, Facultad de Ciencias Biológicas y Ambientales, Universidad de León, León, Spain; 2 Instituto de Biotecnología, Parque Científico de León, León, Spain; University of Alberta, Canada

## Abstract

ArgR is the regulator of arginine biosynthesis genes in *Streptomyces* species. Transcriptomic comparison by microarrays has been made between *Streptomyces coelicolor* M145 and its mutant *S. coelicolor* Δ*argR* under control, unsupplemented conditions, and in the presence of arginine. Expression of 459 genes was different in transcriptomic assays, but only 27 genes were affected by arginine supplementation. Arginine and pyrimidine biosynthesis genes were derepressed by the lack of ArgR, while no strong effect on expression resulted on arginine supplementation. Several nitrogen metabolism genes expression as *glnK*, *glnA* and *glnII*, were downregulated in *S. coelicolor* Δ*argR.* In addition, downregulation of genes for the yellow type I polyketide CPK antibiotic and for the antibiotic regulatory genes *afsS* and *scbR* was observed. The transcriptomic data were validated by either reverse transcription-PCR, expression of the gene-promoter coupled to the luciferase gene, proteomic or by electrophoresis mobility shift assay (EMSA) using pure Strep-tagged ArgR. Two ARG-boxes in the arginine operon genes suggest that these genes are more tightly controlled. Other genes, including genes encoding regulatory proteins, possess a DNA sequence formed by a single ARG-box which responds to ArgR, as validated by EMSA.

## Introduction

Arginine metabolism is feedback repressed by arginine in different Gram-positive and Gram-negative bacteria. This effect is mediated by ArgR, a hexameric protein that represses arginine biosynthesis genes, using L-arginine as co-repressor, in *Escherichia coli* [1] and *Pseudomonas* [2]. A similar effect is exerted by the homologous regulatory protein AhrC in *Bacillus subtilis* [3] or *Lactococcus* [4] and by ArgR in *Corynebacterium* [5].

Characterization of *argR*, encoding the ArgR repressor [6] and the use of AhrC protein permitted to understand the arginine biosynthesis cluster regulation in *Streptomyces clavuligerus* and to locate ARG-boxes, for ArgR binding, upstream of several arginine biosynthesis genes [7]. This provided the basis to study arginine regulation in other *Streptomyces* species. This group of soil-dwelling bacteria produces many secondary metabolites that use arginine, or arginine-related molecules, as precursors. Streptomycin, mitomycin, streptothricin or clavulanic acid are metabolites that contain moieties of guanidine, carbamoyl groups, ornithine or arginine, all of which are compounds related to the arginine biosynthesis or catabolism [8]–[11]. The C5 moiety of clavulanic acid, produced by *S. clavuligerus*, derives directly from an arginine molecule [11]; in addition, undecylprodigiosin [12] and other metabolites contain proline, which as arginine derives from glutamate, and might share common regulatory mechanisms with arginine.

DNA microarrays and proteomic studies are useful tools to understand metabolic pathway regulation in microorganisms. In *Pseudomonas*, which has four arginine catabolic pathways: arginine deiminase (ADI), arginine succinyl transferase (AST), arginine decarboxylase (ADC) and arginine dehydrogenase (ADH), the transcriptomic studies identified 38 genes related to the ADH pathway that are induced by arginine in the absence of ArgR, as well as 27 arginine-induced genes of the AST pathway [2], [13]. Using transcriptomic studies Caldara *et al*. [14] have also found that arginine uptake systems encoded by the *art* and the *hisJQMP* genes are repressed by arginine in *E. coli*.

Transcriptomic studies have been very useful to understand the molecular mechanisms of global control by high hierarchy regulators, for example, A-factor regulation in *Streptomyces griseus* [15] and phosphate and nitrogen regulation in *S. coelicolor* [16]–[17]. These studies allowed us to understand networks interconnecting phosphate and nitrogen metabolism in *Streptomyces* [18], carbon regulation [19] and the connection between the global regulator AfsR, a protein controlling antibiotic production in *S. coelicolor*, and the phosphate regulation exerted by PhoP, the response regulator of the two components of the PhoRP system that controls the *pho* regulon [20].

Therefore, it was convenient to analyze gene expression under control and arginine-supplemented conditions, to obtain better knowledge about arginine transport and catabolism in this model microorganism, and to determine whether ArgR is a regulatory protein involved only in arginine control, or if it has wider regulatory functions.

## Results

### Comparison of *S. coelicolor* strains grown in the presence and absence of arginine

**Figure 1:**
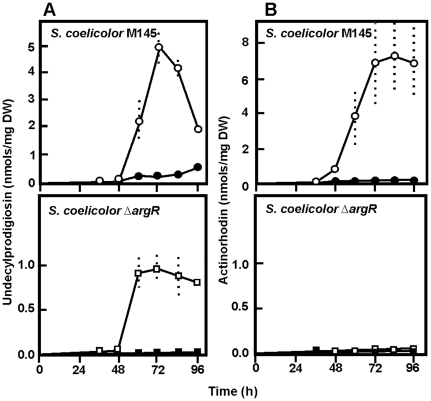
Growth and antibiotic production by *S. coelicolor* M145 and *S. coelicolor* Δ*argR.* (A) Undecylprodigiosin production. (B) Actinorhodin production. Antibiotic production in MG medium by *S. coelicolor* M145 (white circles) and *S. coelicolor* Δ*argR* (white squares) and in MG supplemented with 25 mM arginine (black circles and squares). Vertical bars show standard deviation of three replicates.

Experiments were performed to define the optimal conditions to achieve similar growth kinetics in MG medium, as well as good reproducibility, for the parental strain *S. coelicolor* M145 and the *argR* (SCO1576)-deleted mutant *S. coelicolor* Δ*argR*. A high arginine concentration is required in *Streptomyces* to produce a clear effect on enzymes of the arginine biosynthesis pathway in MG medium [6], [21], therefore, the MG cultures were supplemented with 25 mM arginine. The pattern of arginine utilization was similar for both strains; approximately 15% of the arginine was consumed at 32 h of growth and no arginine was left at 96 h. Antibiotic onset in liquid MG medium using *S. coelicolor* M145 occurred at about 40 h for actinorhodin and 50 h for undecylprodigiosin. Under non-supplemented conditions, *S. coelicolor* Δ*argR* produced only about 20% undecylprodigiosin in relation to the parental strain and lacked completely actinorhodin formation. Arginine supplementation strongly impaired the production of the pigments in both strains (Fig. 1).

### Transcription profiles of *S. coelicolor* M145 and the Δ*argR* mutant in response to arginine

**Figure 2:**
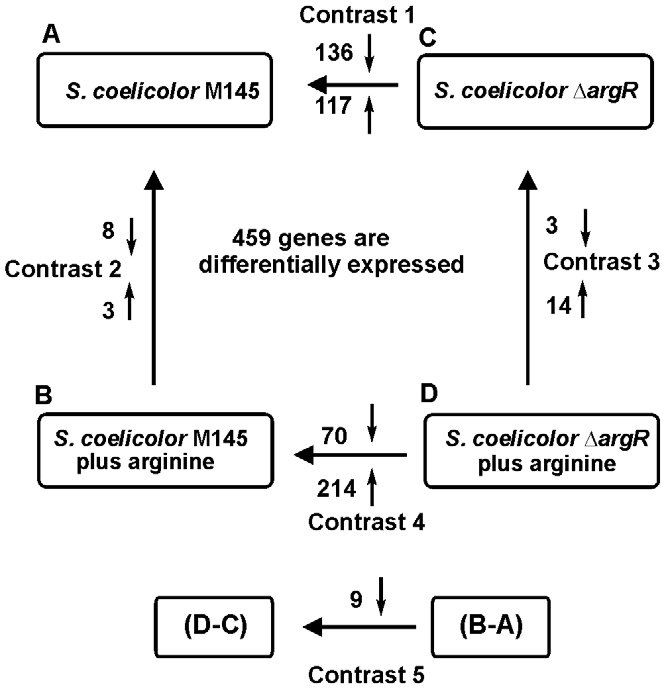
Scheme showing the number of genes differentially expressed in the five contrasts. *S. coelicolor* M145 and *S. coelicolor* Δ*argR* were grown in absence (A and C) or in presence of 25 mM arginine (B and D). The arrow orientation refers to the comparison between two conditions. Differential transcription values were obtained by subtracting the Mg values of the first condition from those of the second condition (corresponding to the arrowheads). The small vertical arrows indicate the number of genes up-expressed (up-oriented arrows) or down-expressed (down-oriented arrows) in each of the contrasts. Contrast 5 is the interaction contrast that reflects the differential response to the arginine supplementation of the two strains.

The statistical analysis of four biological replicates for each experimental condition indicated in Materials and Methods identified 459 genes with significant differential transcription (Dataset S1) in at least one out of the five contrasts shown in Fig. 2. Only 27 genes (6.1%) were differentially expressed in the arginine-supplemented conditions, with respect to the control, indicating a weak transcriptional response to the presence of arginine (Fig. 2, contrasts 2 and 3). Most of the 459 differentially expressed genes corresponded to the comparisons between the wild-type strain and the Δ*argR* mutant in control (contrast 1) or arginine-supplemented (contrast 4) cultures. About 50% of the genes differentially expressed encode membrane proteins, secreted proteins or proteins with unknown functions.

**Figure 3:**
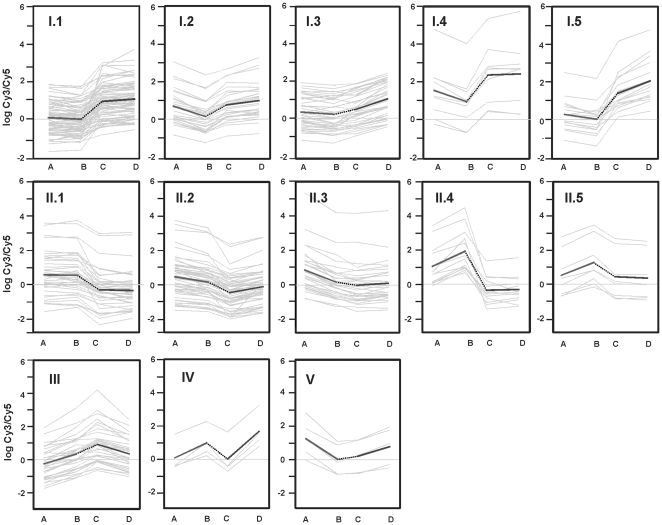
Transcription profiles. Genes differentially expressed in MG cultures of *S. coelicolor* M145 (A); *S. coelicolor* M145 supplemented with arginine (B); *S. coelicolor* Δ*argR* (C) and *S. coelicolor* Δ*argR* supplemented with arginine (D). The genes have been grouped in types and subtypes according with their expression profile. Each grey line corresponds to the change in transcription of a given gene. The thicker line is the transcription mean profile for all the genes in the proposed group.

The transcriptional profiles analysis permitted to establish a classification of the genes into five types. The patterns of genes differentially transcribed in the two strains in unsupplemented or supplemented conditions fell mainly into types I and II (Fig. 3) with different modulations of the expression (subtypes 1–5). Forty-three genes showed the pattern of expression indicated in types III to V (Fig. 3). Here we will analyze general genes included in the five different types, while genes with specific functions will be analyzed in subsequent sections.

#### Type I genes

For type I genes, ArgR appeared to act as a repressor protein. Arginine itself did not affect expression of 76 genes (subtype I.1) and produced a small negative effect in the presence of ArgR or a stimulatory effect in the absence of ArgR in the other 69 genes (subtypes I.2 and I.3, respectively), which was stronger in subtypes I.4 and I.5. The arginine effect, if detected, was negative in the parental strain, but positive in the mutant strain (subtypes I.2–I.5). Thus, arginine appeared to function as a co-repressor for the genes classified into types I.2, I.4 and I.5. Surprisingly, subtypes I.1–I.3 comprise 14 genes for regulatory proteins including regulators of the TetR, DeoR, AraC, GntR or LipR families and sigma, anti-sigma or anti-sigma antagonist factors (Table S1). Other genes of type I encode proteases, peptidases or aminotransferases, adenosine deaminases and glutamyl-tRNA-charging amidotransferases, or are related to amino acid metabolism, such as those for serine hydroxymethyltransferase (SCO4837), NAD-dependent glutamate dehydrogenase (SCO2999), glutamate binding protein (SCO5776), and the B_12_-dependent and the B_12_-independent methionine synthases MetH and MetE (SCO1657 and SCO0985, respectively). Two genes involved in sporulation regulation, and five genes for aerial mycelium formation are also included in this group (Table S1).

#### Type II genes

For type II genes, ArgR behaved as an activator. Addition of arginine (contrasts 2 and 3) did not modify these genes expression (42 genes in type II.1) or produced a slightly positive or negative differential expression in the presence or absence of ArgR, respectively (56 and 32 genes in subtypes II.2 and II.3). Genes encoding regulators of AsnC, AraC, PadR, AbaA, TetR or DeoR type or members of two component systems are present in these subtypes. Type II includes genes for proteases, peptidases and protease inhibitors (Table S1). Some secondary metabolism genes, such as those encoding the putative actinorhodin transporter (SCO5083) and the butyrolactone-binding protein ScbR (SCO6265), belong to this type.

Subtypes II.4 and II.5 include 20 genes that were upregulated by arginine supplementation in the parental strain (contrast 1), but not in the *argR* mutant. The most interesting genes in these subtypes are the sigma-like *afsS* gene (SCO4425), a gene that encodes a tetracenomycin-like transporter (SCO2373) and a member of the two component system for nitrate sensing (SCO1160).

#### Type III genes

This type comprises 34 genes whose transcription was stimulated by arginine in the strain containing ArgR but downregulated in the absence of ArgR (i.e. the opposite behaviour to that of types I.4 and I.5). Genes of relevance in this type are transcriptional regulators (SCO2184 and SCO3769), a member of a two-component system (SCO6421), and the ATP-dependent polyphosphate kinase (SCO1781).

#### Type IV genes

The expression of the four genes of this type was stimulated by arginine, independently on the presence of ArgR. Two of these genes encode, respectively, an amidinotransferase (SCO1222) and the γ-aminobutyric acid aminotransferase (*gabT*, SCO5676).

#### Type V genes

The five genes in this type showed patterns similar to those of types I.2 or I.5, with a more marked effect in the M145 strain. Two genes related to amino acid metabolism, a cysteine desulfurase and a threonine synthase (SCO2146 and SCO4293) are included in this type.

### Regulation of arginine biosynthesis and transport genes as measured by transcriptomics

**Figure 4:**
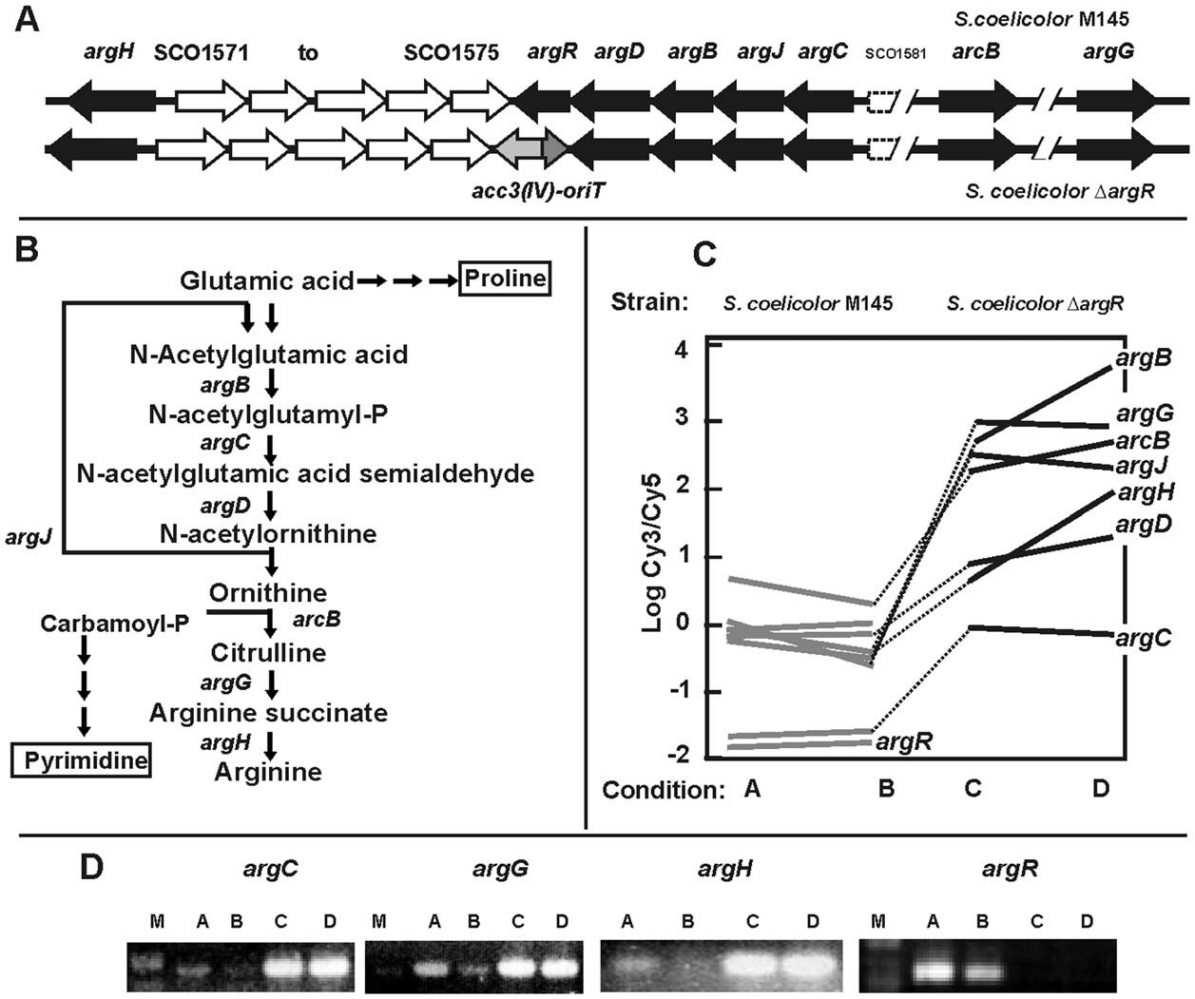
Arginine biosynthesis genes. (A) Cluster of genes for arginine biosynthesis (black arrows) in *S. coelicolor* M145 (above) and the *S. coelicolor* Δ*argR* mutant (below). SCO1581 is indicated with broken lane since it might not be a real gene. (B) Arginine biosynthesis pathway showing its relation to proline and to pyrimidine biosynthesis via carbamoyl-phosphate incorporation. (C) Expression of the arginine biosynthesis genes in A, B, C and D conditions as indicated in Fig. 3. (D) RT-PCR amplification of the *argC, argG*, *argH* and *argR* genes in the same conditions and strains as above. M, Markers.

Arginine biosynthesis genes in *S. coelicolor* are organized in the *argCJBDR* cluster (SCO1580–1576) and in three separate genes: *argH* (SCO1570), *arcB* (SCO5976, located in the *arc* cluster) and *argG* (SCO7036) (Fig. 4). Genes involved in the formation of carbamoyl-phosphate (*carAB*) and required to form citrulline from ornithine will be considered in the next section. Our transcriptional data of the *S. coelicolor* parental strain (containing ArgR) did not show significant arginine-dependent repression for most arginine biosynthetic genes (*argCJD, argG* and *arcB* are in the arginine-not-affected type I.1), in contrast to *E. coli* [14]. On the other hand, arginine supplementation in the Δ*argR* mutant cultures caused a slight upregulation of some *arg* genes as *argH* (type I.3) and *argB* (type I.5) with a 2.4 and 2.1-fold increase, respectively. The main transcriptional differences were the higher transcription in the Δ*argR* mutant (from 1.6-fold in *argD* to seven fold in *argB* or *arcB*) in relation to the wild-type strain. A putative gene, SCO1581, divergent to *argC*, is not differentially expressed in any condition.

The SCO5258–5260 genes, homologous to *Pseudomonas aeruginosa aotJQMOP* encoding proteins for arginine/ornithine uptake, were similarly transcribed in all conditions. However, transcription of the SCO5776–5777 genes, annotated as a putative ATPase and binding proteins for glutamate uptake (an arginine precursor) were negatively modulated by arginine and activated in the Δ*argR* mutant.

### Arginine catabolism

*S. coelicolor* M145 grew well on plates containing ornithine, arginine, urea or citrulline as sole carbon or nitrogen source. No significant differences were found in the growth of the *S. coelicolor* Δ*argR* mutant, with only a slightly lower growth on citrulline.

Of the multiple catabolic pathways for arginine catabolism found in other bacteria, only two catabolic genes, *arcA* and *arcA2* (SCO0613 and SCO5975), both for arginine deiminase, have been identified in *S. coelicolor* genome. They have a high similarity to *Pseudomonas* or *E. coli arcA* genes but they were not differentially expressed in our experimental conditions. All the genes of the ADC pathway of *Pseudomonas*, forming succinate from arginine, have homologous genes scattered in *S. coelicolor* genome (SCO7311, SCO5527, SCO6414, SCO5655, SCO4913, SCO5676 and SCO7035) with e-value scores of 10^−80^ to 10^−101^. These genes were not affected by arginine or by the ArgR regulator, with the exception of *gabT* (SCO5676), which encodes the enzyme that catabolizes 4-aminobutyrate to form succinate semialdehyde. Expression of this type IV gene was twice as high in cultures supplemented with arginine than in unsupplemented cultures of both the M145 and the Δ*argR* strain.

The AST pathway is encoded by the *aruCFGDBE* genes in *P. aeruginosa* [4]. In *S. coelicolor*, an *aruC* homologue, *gabT* (SCO5676, discussed above) was stimulated by arginine and SCO1865 (subtype I.1), encoding an aminotransferase, was overexpressed in the Δ*argR* mutant. Homologues of *aruF* or *aruG* are not present in *S. coelicolor* and those genes encoding proteins similar to AruD did not show differential expression under any conditions.

Genes encoding standard arginases (EC.3.5.3.1) have not been found in the *S. coelicolor* genome. Nevertheless, arginase activity is possibly associated with the carbamoyl-phosphate transferase activity of ArcB, as occurs in *S. clavuligerus* [22]. Expression of *arcB* (and the putative arginase activity) was strongly repressed in the presence of ArgR, as indicated above. No effect due to arginine or to the lack of ArgR was observed in the urea-utilizing genes (*ureDGFCBA,* SCO1231–1236, and *ureABC*, SCO5525–5526).

### Nucleotides and nucleic acid-biosynthesis-related genes

**Figure 5:**
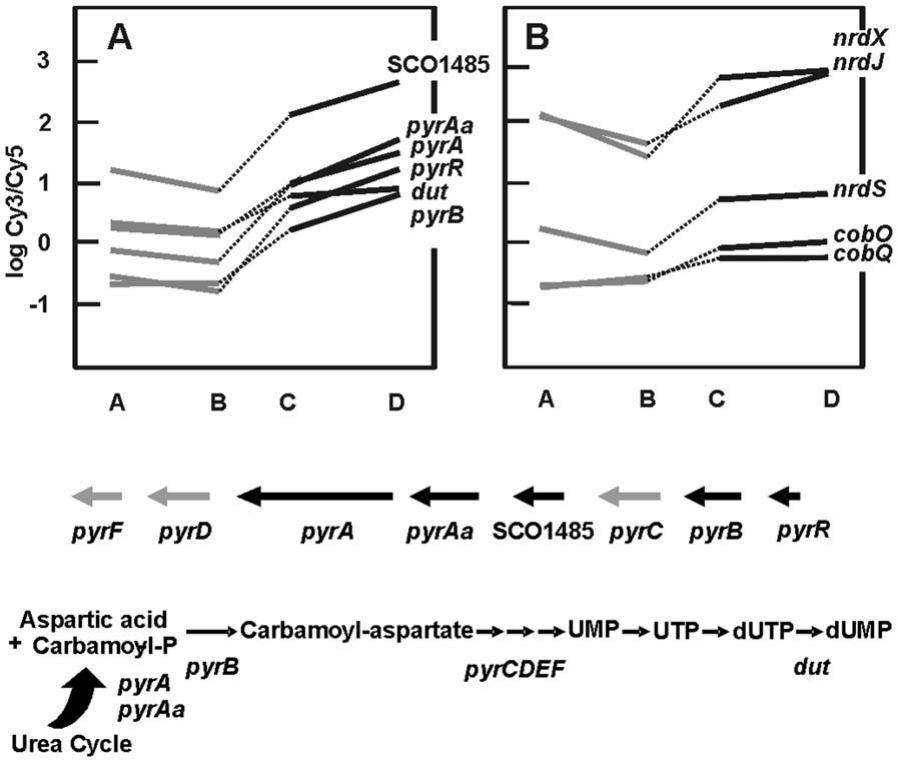
Differential expression of nucleotides biosynthesis genes. (A) Expression of genes related to pyrimidine biosynthesis. A scheme of the gene cluster and biosynthesis pathway is shown below. Genes marked as black arrows were differentially expressed. (B) Expression of genes related to deoxyribonucleotides and cobalamin biosynthesis. A, B, C and D correspond to the conditions indicated in Fig. 3. Notice that *nrdX* is not a real gene but a transcribed regulatory intergenic region.

Pyrimidine biosynthesis is related to arginine biosynthesis through the utilization of carbamoyl-phosphate (CP). CP is formed by the CP synthase, encoded by *pyrA* and *pyrAa* (Figs. 4 and 5) and condensed to ornithine by the ornithine carbamoyltransferase (*arcB*) to yield citrulline, and to aspartate to form carbamoyl-aspartate, by the aspartate carbamoyl transferase (*pyrB*). Both *arcB* (I.1 subtype) and *pyrB* genes were upregulated in the Δ*argR* mutant.

In the pyrimidine pathway, carbamoyl-aspartate is sequentially converted in UMP through the action of enzymes encoded by the *pyrB, pyrC, pyrD, pyrE* and *pyrF* genes [23], clustered in *S. coelicolor*, with exception of *pyrE* (Fig. 5A). Several genes in this cluster showed a significantly higher expression in the Δ*argR* mutant than in the parental strain (1.6–2.2-fold; contrast 1). Addition of arginine slightly reduced their transcription in the M145 strain but it increased in the Δ*argR* mutant (2.7–4-fold; contrast 4). Two sets of these genes were especially affected: *pyrR–pyrB* and SCO1485*–pyrA–pyrAa* showing profiles of type I.5, except for SCO1485 which was of type I.4. No significant differential expression of the *pyrE* gene, located outside of the cluster, was detected.

Expression of other genes directly or indirectly, involved in nucleotide metabolism was higher in the *argR* mutant. They included SCO2015 (encoding a putative secreted nucleotidase), SCO5662 (adenosine deaminase), SCO3060 (*purK*, phosphoribosylaminoimidazole carboxylase ATPase), SCO5743 (*thyX,* thymidylate synthase), and SCO5868 (*dut*, deoxyuridine 5′-triphosphate nucleotidohydrolase).

Two sets of independent ribonucleotide reductases (RNRs) *in Streptomyces* are encoded by the *nrdABS* (SCO5226–5224) and *nrdRJ* clusters (SCO5804–5805). The first cluster encodes the oxygen-dependent RNR and is regulated by a B_12_-dependent riboswitch located upstream of *nrdA* [24]. The microarray probes for the riboswitch region (associated to the fake gene *nrdX* because of the original genome annotation), the *nrdS* (encoding an AraC-like regulator) and the *nrdJ* (encoding the class II RNR) genes, indicated a 30–40% expression decrease in arginine supplemented cultures of the wild-type strain, and a higher expression in the Δ*argR*-mutant (up to 1.5-fold), especially in the presence of arginine (Fig. 5B). Two genes, *cobQ* and *cobO* (SCO1848 and SCO1851) for B_12_ biosynthesis had the type I.1 profile.

### Effect of arginine on the nitrogen metabolic network

The nitrogen utilization network in *Streptomyces* uses the glutamine synthetase, encoded by *glnA*, as a key enzyme and is strictly regulated by the OmpR-like regulators GlnR and GlnRII [17].

Our data showed that the transcription of several nitrogen metabolism genes was controlled by ArgR. The *glnK* gene (SCO5584), encoding a PII-like regulator, was downregulated in the Δ*argR* mutant (II.2 type). The same regulation was found for the *glnK* flanking genes, *amtB* and *glnD,* encoding respectively an ammonium tranporter and the PII-modifying adenylyltransferase. Similarly, *glnII* (SCO2210) that encodes the eukaryotic-like glutamine synthetase GlnII, and *glnA* (SCO2198) that encodes the prokaryotic type I glutamine synthetase, showed profiles of type II.1 and II.4. Therefore, all these genes appeared to require ArgR for optimal expression. No differences in transcription were found for the regulators *glnRII* and *glnR* (SCO2213 and SCO4159, respectively). Thus, it seems that the positive regulation of ArgR on nitrogen metabolism is not mediated by GlnR or GlnRII.

### Effect on morphology, differentiation and secondary metabolism genes

Arginine addition or deletion of *argR* resulted in a drastic reduction of actinorhodin and undecylprodigiosin, or even in complete lack of production of the latter pigment (Fig. 1). Indeed, two genes of the *act* pathway, *actII-orf2* (SCO5083) encoding a putative actinorhodin transporter [25] (profile II.3) and the ketoacylreductase ActIII (SCO5086; II.4 profile) were affected. Probably due to the early time of culture used to extract RNA, no differences were found in expression of the undecylprodigiosin-encoding genes. The effect on production of this antibiotic (Fig. 1) was not related to the supply of the proline precursor, since the three genes of the proline pathway (*proABC,* SCO2585, SCO2587 and SCO3337) were equally expressed in both strains or culture conditions.

Two regulatory genes, essential for antibiotic formation, *afsS* (SCO4425) encoding a sigma-like protein and *scbR* (SCO6265) for a butyrolactone-receptor protein [26]–[27] showed II.5 and II.1 profiles, respectively, with a strong decrease in expression in the Δ*argR* mutant. The repression in *afsS* may also contribute to the drastic reduction of both pigmented antibiotics.

**Figure 6:**
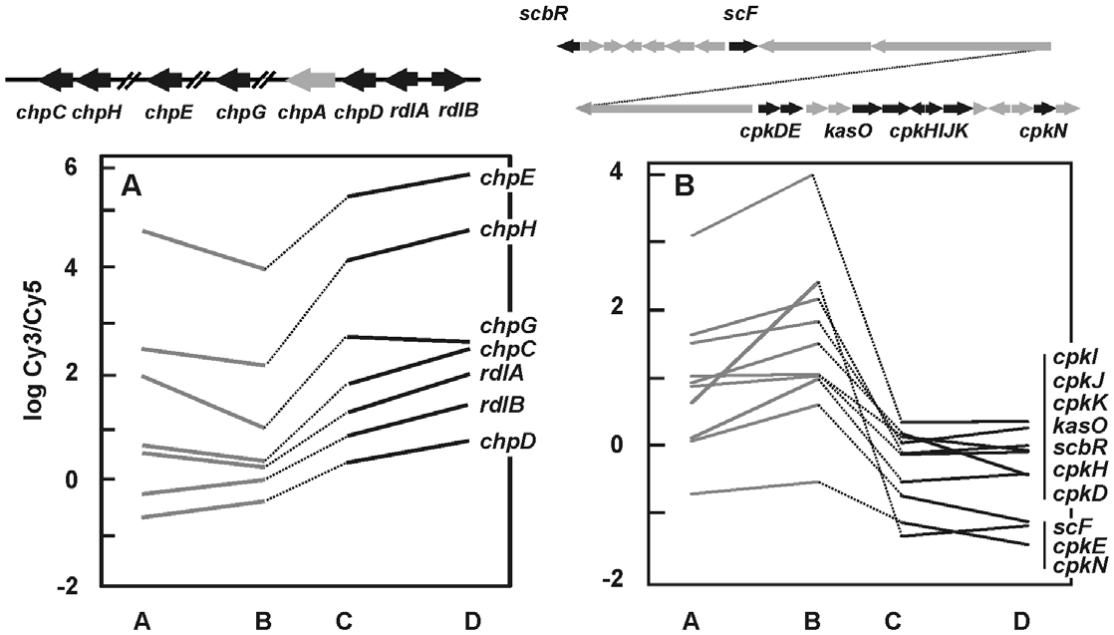
Differential expression of genes related to morphology and secondary metabolism. (A) Transcriptomic of genes for rodlins and chaplins. The cluster *chp–rdl* is shown above with differentially expressed genes in black. (B) Transcriptomic of the genes for CPK biosynthesis. The *cpk* cluster is shown above. Genes with differential expression are indicated in black. Included are the genes for *scbR*, for a butyrolactone receptor protein, and *scF,* for a putative secreted FAD-binding protein. A, B, C and D correspond to the conditions indicated in Fig. 3.

A very strong effect due to the lack of ArgR was observed on the biosynthesis of rodlins and chaplins, required to form aerial mycelium and spores [28], and on the genes encoding the yellow polyketide pigment CPK [29] (Fig. 6). Genes for chaplins are located in five different sites in the genome being *chpA* and *chpD* clustered with *rdlA* and *rdlB* for rodlins (SCO2716–2719). Although expression of *chpF* and *chpA* was not affected, the other genes showed profiles of the I.4 type (*chpG* and *chpE*, SCO2699 and SCO1800, respectively) and I.5 type (*chpH*, *rdlA* and *chpC,* SCO1675, SCO2718 and SCO1674, respectively).

Most genes of the *cpk* cluster were downregulated in the Δ*argR* mutant. In the parental strain, higher transcription of some genes, especially *cpkE*, was detected in arginine-supplemented cultures. The profile of these genes was of II.1 type (*kasO, cpkJ, cpkN*, SCO6280, SCO6283 and SCO6288, respectively) or II.4 type (*cpkDE, cpkHI, cpkK*, SCO7276–7277, SCO6281–6282 and SCO6284, respectively) (Fig. 6B). The observed effect was probably due to a cascade effect mediated by the *Streptomyces* type of regulators encoded by *kasO* and *cpkN*, which were downregulated in the Δ*argR* mutant, but it may also reflect a general effect of ArgR on the glutamate supply, because the CPK antibiotic production is stimulated by glutamate [29]. Two genes located adjacent to the *cpk* cluster, *scbR* (already mentioned) and *scF* for a putative secreted FAD-binding protein, were also affected by the lack of ArgR (Fig. 6B).

### Proteomic studies

**Figure 7:**
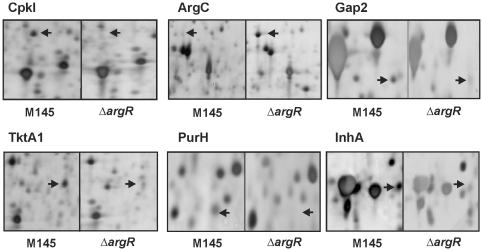
Proteomic Analysis. Detailed view of 2D-SDS-PAGE gels showing differences on the proteomes of *S. coelicolor* M145 (left panels) and *S. coelicolor* Δ*argR* (right panels). Arrows indicate the protein spots differentially represented corresponding from left to right in the upper panels to CpkI (SCO6282), ArgC (SCO1580), and Gap2 (SCO7511), and in the lower panels to TktA1 (SCO1935), PurH (SCO4814) and InhA (SCO1814).

Comparative analysis of the proteomes of *S. coelicolor* wild-type and Δ*argR* mutant grown with and without arginine supplementation revealed no significant differences, confirming the low effect of arginine detected in the transcriptomic studies. The largest proteome differences were found in the comparison of the wild-type and the Δ*argR* mutant. Twenty-six differentially represented proteins were identified, several of which were consistent with the transcriptomic results. They are listed in Table 1 (see Fig. 7 and Dataset S2 for details).

**Table 1:** Proteins differentially represented in *S. coelicolor* and *S. coelicolor* Δ*argR.*

spot IN(1)	SCO n°(2)	gene name	annotated function(3)	*p*-value	up/down in Δ*argR*
6149	SCO0379	*katA*	catalase	0.000	down
6577	SCO1570^(a)^	*argH*	argininosuccinate lyase	0.005	up
6655	SCO1580^(a)^	*argC*	N-acetyl-gamma-glutamyl-phosphate reductase	0.000	up
6550	SCO1814	*inhA*	enoyl-(acyl carrier protein) reductase	0.007	Down
6445	SCO1935	*tktA1*	transketolase	0.033	Down
6497	SCO1945	*tpiA*	triosephosphate isomerase	0.015	Down
6233	SCO1946	*pgk*	phosphoglycerate kinase	0.008	Down
6193	SCO1947	*gap1*	glyceraldehyde-3-phosphate dehydrogenase	0.029	Down
6666	SCO1965		export associated protein	0.015	Down
6494	SCO1998	*rpsA*	30S ribosomal protein S1	0.009	Up
6143	SCO2198^(a)^	*glnA*	glutamine synthetase I	0.000	Down
6647	SCO2368		hypothetical protein	0.007	Down
6685	SCO2633	*sodF*	superoxide dismutase	0.011	Up
6366	SCO3649	*fba*	fructose-bisphosphate aldolase	0.012	Down
6264	SCO4770	*guaB*	inosine-5′-monophosphate dehydrogenase	0.032	Down
6269	SCO4771		inositol-5′-monophosphate dehydrogenase	0.040	Down
6400	SCO4809	*sucD*	succinyl-CoA synthetase subunit alpha	0.045	Down
6125	SCO4814	*purH*	formyltransferase/IMP cyclohydrolase	0.030	Down
6167	SCO4837^(b)^	*glyA*	serine hydroxymethyltransferase	0.010	Down
6678	SCO4856^(a)^	*sdhA*	succinate dehydrogenase flavoprotein subunit	0.000	Up
6257	SCO4958	*metB*	cystathionine gamma-synthase	0.027	Down
6340	SCO6282^(a)^	*cpkI*	enoyl-(acyl carrier protein) reductase	0.017	Down
6513	SCO7036^(a)^	*argG*	argininosuccinate synthase	0.000	Up
6771	SCO7510	*cypH*	peptidyl-prolyl cis-trans isomerase	0.009	Down
6317	SCO7511	*gap2*	glyceraldehyde 3-phosphate dehydrogenase	0.026	Down

(1) spot identification number automatically generated from *ImageMaster* software.

(2) Among the 459 differentially transcribed genes, the products of seven genes were detected in the Proteomics experiments. Six genes showed the same transcriptomic and proteomic profile ^(a)^ and only one gene gave opposite results ^(b)^.

(3) from ScoDB.

#### Arginine biosynthesis

In agreement with the transcriptomic results, several enzymes belonging to the arginine biosynthetic pathway were identified as protein spots only in the proteome of the mutant strain Δ*argR*. These included the N-acetyl-γ-glutamyl-phosphate reductase ArgC (SCO1580, spot IN 6655) and the last two enzymes in the pathway, argininosuccinate lyase ArgH (SCO1570, spot IN 6577), and argininosuccinate synthase ArgG (SCO7036, spot IN 6513).

#### Nitrogen metabolism proteins

Another transcriptomic and proteomic matching result was for glutamine synthetase I, GlnA (SCO2198, spot IN 6143), which was under-represented in the mutant proteome, confirming that *glnA* expression required ArgR as transcriptional activator. As glutamine is the amino donor for carbamoyl-phosphate synthesis, ArgR may influence the urea cycle and the pyrimidine biosynthesis by controlling the glutamine levels.

#### Pentose phosphate and glycolysis enzymes

Several proteins related to these pathways are under-represented in the *S. coelicolor* Δ*argR* proteome as compared with the wild-type. These the proteins are the transketolase TktA1 (encoded by SCO1935, spot IN 6445), that interconverts D-fructose-6-phosphate and D-xylulose-5-phosphate in the pentose phosphate pathway, which finally results in formation of D-glyceraldehyde-3-phosphate; the triose phosphate isomerase TpiA (SCO1945, spot IN 6497), which interconverts glyceraldehyde-3-phosphate and hydroxyacetone; the phosphoglycerate kinase Pgk (SCO1946, spot IN 6233) and a glyceraldehyde-3-phosphate dehydrogenase isoenzyme Gap1 (SCO1947, spot IN 6193). Also, the deoxyribose-phosphate aldolase encoded by SCO4914 (spot IN 6381), the fructose biphosphate aldolase Fba (SCO3649, spot IN 6366), and the second glyceraldehyde-3-phosphate dehydrogenase Gap2 (SCO7511, spot IN 6317), were under-represented in the Δ*argR* mutant when compared to the wild-type strain. These proteomic results revealed that glycolytic and pentose phosphate pathways were more active in the wild-type strain.

#### Proteins related to succinate metabolism

Two of the enzymes involved in arginine biosynthesis, ArgH and ArgG, have succinate either as substrate or reaction product. Surprisingly several proteins using succinate were under-represented in the mutant proteome. This is the case of MetB (SCO4958, spot IN 6257) a cystathionine γ-synthase that uses O-succinyl/acetyl homoserine as substrate and SucD (SCO4809, spot IN 6400) the α subunit of succinyl-CoA synthetase. Although no significant changes in the expression of these genes were observed, other genes related to succinate metabolism showed a differential expression in transcriptomic experiments. That is the case for *gabT* (forming succinate), the adenylosuccinate-synthetase-encoding gene *purA* (SCO3629, profile II.2) and for *dhsA* (SCO4856), *dhsB* (SCO4855) and SCO0922, three out of the 10 genes encoding succinate dehydrogenases which showed I.3 profile.

#### Purine metabolism

In the proteome of the wild-type strain, three protein spots involved in purine metabolism were detected, which were under-represented in the mutant strain. Proteins with inosine 5′-monophosphate (IMP) dehydrogenase (SCO4770) and 5-inositol-5-monophosphate dehydrogenase (SCO4771) were identified in spots IN 6264 and IN 6269, respectively. These activities carry out the conversion of IMP to xanthosine 5′-phosphate. The same profile was shown by the PurH protein, (spot IN 6125), encoded by SCO4814 involved in the synthesis of 1-(5′-phosphoribosyl)-5-formamide-4-imidazole carboxamide which connects purines and histidine pathways.

#### Other proteins

Three polyketide and fatty acid biosynthesis enzymes were also under-represented in the mutant proteome. The 3-oxoacyl-ACP reductase CpkI (encoded by SCO6282, spot IN 6648), required for the production of the yellow-pigmented CPK associated metabolite [29] which was consistent with the transcriptomic results. The enoyl-ACP-reductase FabI/InhA (SCO1814, spot IN 6550) was also under-represented.

Post-transcritional regulation, protein turnover, mRNA stability or translation efficiency might account for the low correlation in the transcriptomic and proteomic results in some cases [30].

### Validation experiments: RT-PCR and heterologous genes expression

In addition to proteomic studies, validation of the transcriptomic results was performed for nearly 40 representative genes by either (i) RT-PCR, (ii) coupling promoters to the *Vibrio harveyi* promoter-less *lux*AB genes to measure luciferase activity and (iii) EMSA using a recombinant Strep-ArgR fusion protein (Table 2).

**Table 2:** Validation assays. Summary of EMSA, promoter probe assays or RT-PCR.

	EMSA					Promoter-*luxAB* fusion					RT-PCR	
Promoter region	Probe chromosome coordinates	*R*i (bits) of possible ARG boxes			Strep-ArgR binding(1)	Gene	Ratio of contrast(2)				Gene assayed	Results (3)
							1	2	3	4		
SCO0800–SCO0801	847420–847787	4,1			ND	–					–	
SCO1086	1146430–1146605	3,4	16,5		+	–					–	
SCO1220–SCO1221	1291917–1292299	5,2			ND	–					–	
SCO1236 (*ureA*)	1310577–1310873	7,5			+	–					–	
SCO1483 (*pyrA*)	1587383–1587719	1,8			+	–					*pyrA*	↑Δ*argR*
SCO1487 (*pyrB*)	1591391–1591624	7,1			+	*pyrB*	Low(4)	Low(4)	Low(4)	Low(4)	–	
SCO1488 (*pyrR*)	1592025–1592381	7,7	5,1		+	*pyrR*	2.3(5)	2.3	1.6(5)	1.6(5)	*pyrR*	↑Δ*argR*
SCO1570 (*argH*)	1681863–1682008	17,3	7,7		+	*argH*	2.2	0.3	2.2(5)	13.9(5)	*argH*	↑Δ*argR*
SCO1580 (*argC*)*-*SCO1581	1691340–1691628	12,1	9,4	6,1	+	–					*argC*	↑Δ*argR*
SCO2014 (*pyk1*)-SCO2015	2157777–2157998	8,2			+	*pyk1*	Low(4)	Low(4)	Low(4)	Low(4)	–	
SCO2054 (*hisD*)-SCO2055	2202564–2202769	5,7	4,4		+	*hisD*	NS	2.0	NS	NS	–	
SCO2210 (*glnII*)(6)	2373630–2374004	5,3			ND	–					*glnII*	↓Δ*argR*
SCO2231 (*malE*)-SCO2232 (*malR*)	2400346–2400648	9,3	8,6		+	–					–	
SCO2686	2930701–2931003	12,8			+	–					–	
SCO3034 (*whiB*)	3321020–3321291	6,0			+	*whiB*	NS	0.5(5)	NS	1.7(5)	*whiB*	↑Δ*argR*
SCO3067–SCO3068 (*sig15*)	3360383–3360707	5,2			+	–					–	
SCO3943 (*rstP*)	4339152–4339343	9,2			+	–					–	
SCO3978–SCO3979	4381777–4382142	10,1			+	–					–	
SCO4158(7)	4381777–4382142	7,4			ND	–					–	
SCO4293	4708341–4708657	9,1			+	–					–	
*leuA*-SCO2529	2727205–2727535	10,4			+	*leuA*	NS	NS	NS	1.3	–	
SCO5226 (*nrdA*)	5688147–5688631	4,6	6,0		+	*nrdA*	NS	NS	1.6	1.4(5)	–	
SC5583 (*amtB*)	6085631–6086027	8,3			+	*amtB*	NS	1.5	0.01	0.01(5)	–	
SCO5864	6421240–6421614	7,0			+	–					–	
SCO5976 (*arcB*)	6550204–6550395	10,9	9,5		+	*arcB*	3.6(5)	2.0	2.3	4.1(5)	–	
SCO7036 (*argG*)	7824665–7824928	8,1	16,7		+	–					*argG*	↑Δ*argR*
SCO7302–SCO7303	8110855–8111154	7,4	4,6		ND	–					–	
SCO7314	8120320–8120720	7,5			+	–					–	
–											SCO1485(8)	↑Δ*argR*
–											SCO1674 (*chpC*)(9)	↑Δ*argR*
–											SCO1800 (*chpE*)(9)	↑Δ*argR*
–											SCO2198 (*glnA*)(9)	↓Δ*argR*
–											SCO2718 (*rdlA*)(9)	↑Δ*argR*
–											SCO5584 (*glnK*)(10)	↓Δ*argR*
–											SCO6265 (*scbR*)(11)	↓Δ*argR*
–											SCO6282 (*cpkI*)(9)	↓Δ*argR*
–											SCO6283 (*cpkJ*)(9)	↓Δ*argR*

(1) ND, not detected.

(2) Values are ratios of specific luminescences at 32 h of culture. Values are shown when t-test *p*-value <0.05 (4 biological replicates for each condition). NS, no significant differences. 1, 2, 3 and 4 as in Figure 2.

(3) Relative up or down expression of the gene in mutant strain.

(4) Luminescence measures too low (<1 arbitrary unit).

(5) Microarray results (when uncorrected p-value <0.05 for the contrast, if not, no comparison was done) showed the same sign of regulation.

(6) The probe does not correspond to the promoter region but to the upstream gene SCO2209 which contains the predicted ARG box.

(7) The predicted ARG box is located in the 25 nucleotides of the 3′end of SCO4159 (*glnR*).

(8) Predicted polycistronic transcript from SCO1488 (*pyrR*).

(9) Putative ARG boxes not found (*R*i≥3, 800-nt region centered at the start codon).

(10) Predicted polycistronic transcript from SCO5583 (*amtB*).

(11) Predicted ARG box overlapping the start codon, *R*i 7.7 bits.

**Figure 8:**
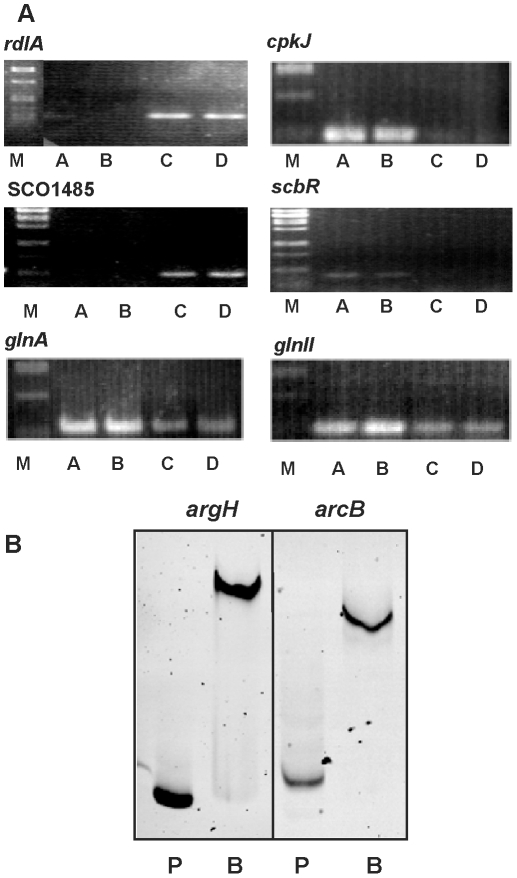
Validation experiments by RT-PCR and EMSA. (A) RT-PCR amplification of mRNA corresponding to the *rdlA*, *cpkJ*, (upper panels), SCO1485, *scbR* (medium panels), *glnA* and *glnII* (lower panels) genes. A, B, C and D correspond to the conditions indicated in Fig. 3. M, Marker. (B) EMSA of 6-FAM labelled *argH* (199 bp) and *arcB* (251 bp) promoters using Strep-ArgR protein. P indicates the free probe and B the binding reaction.

Genes for arginine biosynthesis (*argC*, *argR*, *argH* and *argG*), secondary metabolism (*cpkJ* and *cpkI*) and morphology (*whiB*, *scbR*, *rdlA*, *chpE* and *chpC*), controlling nitrogen metabolism (*glnA*, *glnK* and *glnII*), and for pyrimidine biosynthesis (*pyrA*, *pyrR* and SCO1485), were tested. In all cases, the amplification pattern observed was concordant with the microarray experiment (Figs. 4D and 8A).

**Figure 9:**
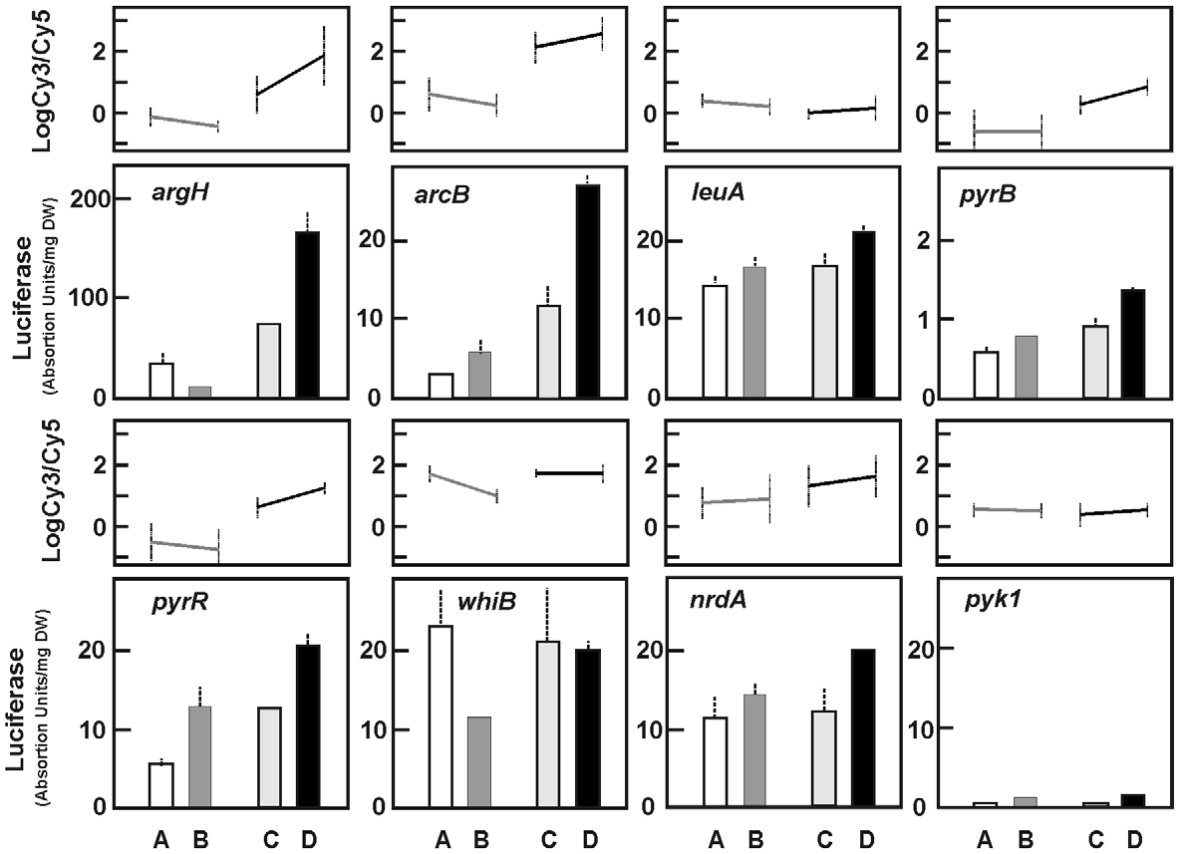
Validation experiments by heterologous gene expression. Luciferase activity of *luxAB-*fused promoters transformed in the adequate *S. coelicolor* strain and grown in the A, B, C and D conditions indicated in Fig. 3. Activity corresponds to the promoter of *argH*, *arcB*, *leuA*, *pyrB* (upper panels) *pyrR*, *whiB*, *nrdA* and *pyk1* (lower panels). On top of each panel is shown the expression profile of the corresponding gene in the transcriptomic studies. The luciferase activity values correspond to 32-h cultures. Vertical bars show standard deviation of two biological replicates measured twice.

Promoters of differentially expressed genes were fused to the promoter-less *luxAB* genes; these constructions were introduced into *S. coelicolor* M145 and *S. coelicolor* Δ*argR* and cultured in MG medium with or without arginine supplementation. The promoters tested were those belonging to *argH* and *arcB* for arginine biosynthesis, *pyrR*, *pyrB* and *nrdA* (SCO5226) related to nucleotide biosynthesis, *leuA* for amino acids biosynthesis, *whiB* for morphological differentiation, and SCO1086, a strongly ArgR affected gene, of unknown function. The promoter of the *pyk1* gene, which is constitutively expressed in our culture conditions, was used as a negative control. In general, the luminescence values in *S. coelicolor* Δ*argR* exconjugants correlated well with the transcriptomic experiment, although *S. coelicolor* M145 exconjugants in the presence of arginine gave a slightly higher activity than that expected from the microarray expression data (Fig. 9).

### Identification of ArgR binding sites

With the aim of finding putative ArgR binding sites that correlated with the differential expression results, we made a matrix (model 1) from the alignment of the 16 experimentally tested *S. clavuligerus* ARG boxes upstream of *argC*, *argG*, *argH* and *arcB* [6] and their complementary sequences. The *S. coelicolor* 5′ regions were scanned by means of the Patser algorithm and the RSA tools server. A neutral separator of two positions was inserted between a duplicated matrix to take into account the tandem arrangement of the ARG boxes. Using model 1 the only sequences with *R*i values (individual information content) higher than 12.2 were those of the above ARG boxes, and a new one in the promoter region of SCO1086. This gene encodes a protein with a transglutaminase-like motif (PF01841) that might be a protease according to Makarova *et al.* [31] and is repressed by ArgR (1.5 profile) as predicted by Castro-Melchor *et al.* [32].

**Figure 10:**
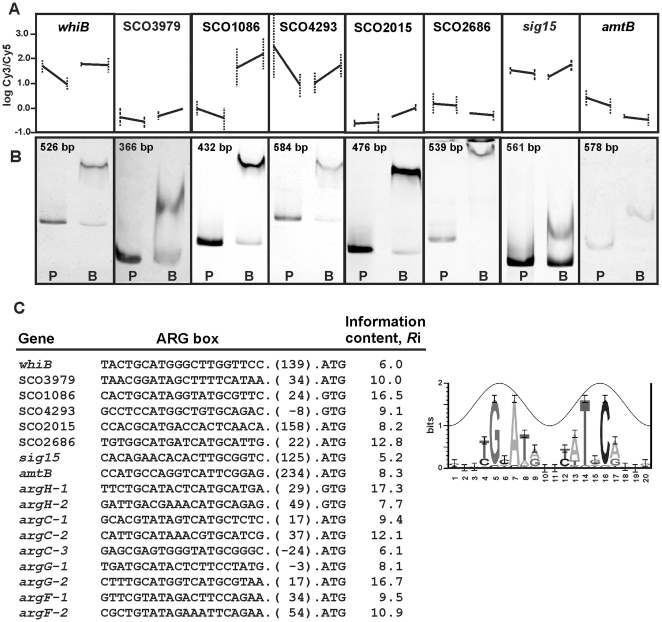
Functional analysis of ARG boxes by EMSA. (A) Transcriptomic expression of the tested genes in the A, B, C and D conditions indicated in Fig. 3. (B) EMSA of PCR-amplified DNA fragments containing putative ARG boxes located upstream of the indicated genes. In all cases, P indicates the free probe and B binding reaction using ArgR (0.8 µM protein). The size of the probes is indicated in the panels. (C) Sequence of the putative ARG box present upstream of the indicated gene and *R*i value determined using the ARG box model represented on the right. The numbers in parenthesis indicate the nucleotides to the translation start codon. The *argH*, *argC*, *argG* and *arcB* ARG boxes are included for comparison.

Although no more ArgR binding sites were detected, more ArgR binding sites in *S. coelicolor* genome could have been expected. They might include low-conserved ARG boxes, boxes with different length of separation, or single (half-length) ARG boxes, as occurs in *Thermotoga* or *B. subtilis* [33]–[34]. A single putative ARG box of *R*i 10.4 was found upstream of the *leuA* (SCO2528) gene. This region was found to be clearly retarded by ArgR. The 22 ARG boxes of *argC*, *argG*, *argH*, *arcB,* SCO1086 and *leuA* were integrated in a new weight matrix. In the new ARG box *Streptomyces* model (model 2, Fig. 10C), the binding site was formed by an imperfect 20 nt palindromic sequence. This matrix allowed us to locate 1583 ARG box candidates with a Ri>5. Manual selection of them was performed, paying attention to those located in the neighbouring of differentially transcribed genes.

Nearly thirty DNA fragments containing putative ARG boxes were used in binding assays in the presence of Strep-ArgR in EMSA experiments. They correspond to upstream regions of: (i) genes related to amino acids biosynthesis (other than arginine) such as SCO4293 for a putative threonine synthase, *leuA* or the bidirectional promoter region *hisD*–SCO2055; (ii) genes for nucleotide biosynthesis or regulation as *nrdA,* SCO2015 or *rstP*; (iii) genes related to nitrogen or carbon metabolism: *glnII*, *amtB*, *glnR* (internal), SCO1086, *ureA* (SCO1236) or the bidirectional promoter region *pyk1*–SCO2015; (iv) genes for transcriptional regulators: *whiB*, *malE*–*malR*, SCO2686, SCO7302–7303, SCO1220–1221, SCO3978–3979, SCO0800–0801, or for sigma or anti-sigma factors: SCO3067–*sig15*, SCO7314; (v) genes encoding hypothetical conserved proteins (SCO5864); and (vi) genes related to arginine (*argH*, *arcB*; *argG*, *argC* and SCO1086) or pyrimidine (*pyrR*, *pyrB* and *pyrA*) biosynthesis as positive controls. The tested ARG boxes have *R*i values that range from 5 (intergenic SCO3067–*sig15* region) to 17 (upstream of *argH*). In most of the analyzed regions the ARG box (20 nucleotides) is unique, but in some cases two ARG boxes, separated from 1 to 208 nucleotides, can be identified. The two ARG boxes upstream of *argH*, *arcB*, *argG* and *argC* are adyacent.

Twenty-four fragments were bound by ArgR. In general a good correlation with the gene expression in transcriptomic studies was found (Figs. 8, 10 and Table 2). Only occasionally, probe shift was unclear or not present (i.e. for *glnII*, *glnR*, SCO7302–7303, SCO0800–0801 and SCO1220–1221). The occasional lack of relation between ARG box and differential expression in the microarray experiments will be discussed below.

## Discussion

ArgR is known as the regulator of the arginine biosynthesis pathway and, in most bacteria, the *argR* gene is clustered with arginine biosynthesis or transport genes [7], [35].

The transcriptomic and proteomic studies shown in this work indicated that in addition to arginine biosynthesis ArgR controls directly or indirectly 452 genes (contrasts 1 and 4, Fig. 2) involved in different aspects of nitrogen metabolism, purine and pyrimidine biosynthesis, cell morphology, and antibiotic production. More interesting is the effect of ArgR on the expression of genes encoding general regulators (GntR-, AbaA- and TetR-like regulators), sigma factors (BldN, SigR, AfsS), or two component regulatory systems, which could be mediated by a cascade regulatory mechanism. Binding assays validated the ARG boxes detected in Figure 10 and in most of the promoters shown in Table 2.

In *E. coli* arginine acts as co-repressor binding ArgR and controlling arginine biosynthesis. However, in *Streptomyces* the effect of arginine as co-repressor is weak and high levels of arginine (25 mM) were required to exert effect on the expression of only 27 genes (contrasts 2 and 3, Fig. 2).

Addition of arginine to *S. coelicolor* cultures reduced the production of actinorhodin and undecylprodigiosin by 92 and 99%, respectively, even though none of the pigmented antibiotic derives directly from arginine. Even more interesting was the observation that the Δ*argR* mutant showed a strong reduction (80%) in undecylprodigiosin production in relation to the wild-type strain and lacked detectable actinorhodin production. Therefore, ArgR is a regulator required for production of these secondary metabolites.

This drastic effect of ArgR on the production of both antibiotics might be mediated by the transcriptional response of the global positive regulator *afsS*. In addition, the effect on actinorhodin production could be correlated with the lower expression of *actIII* and *actII-orf2* in the Δ*argR* mutant. The transporter protein encoded by *actII-orf2* is essential for actinorhodin secretion [25] because accumulation of intracellular antibiotic feedback represses the biosynthetic genes. Identification of additional control mechanisms related to antibiotic production will require further experiments using RNA samples taken during the production phase, because the samples for this omics analyses were taken at an earlier time (32 h) than that of intense antibiotic production.

The ArgR protein acts as a transcriptional regulator after binding to the promoters of the *arg* regulon genes. The binding sites are composed of imperfect palindromes, known as ARG boxes [36]–[38]. Both the *argR* genes and the ARG boxes are well conserved among different bacteria [33], [39]. In *E. coli* the binding sites are composed of two ARG boxes of 18 bp that, with only one known exception, overlap the promoter. The separation between boxes is 3 bp except the 2 bp of separation of the *argR* operator [40], [41]. ArgR functions in *E. coli* as a repressor on arginine biosynthesis and transport genes [14], [42] and on the glutamate synthase operon [43], but as activator of the *ast* operon [44]. The *B. subtilis* AhrC protein – the ArgR orthologue –, which was found to bind *S. coelicolor argC* promoter [45], is also a repressor of the arginine biosynthesis genes [46], [47] and an activator of the arginine catabolism genes in *Bacillus* [48]; similar regulatory behavior is found for ArgR in *P. aeruginosa* and *Salmonella* [35], [49]. In *B. subtilis argC* gene two operators have been found. The operator with the highest affinity for AhrC is formed by two ARG boxes, separated by 11 bp. The second operator, within *argC* coding region, has a single ARG box. A model for the holo-AhrC-*argC* complex proposes that the AhrC hexamer interacts with the three ARG boxes [34]. Other operators formed by a single ARG box are found in the *Bacillus rocA* and *rocD* catabolic genes and show a lower affinity for AhrC [48].

We identified by DNase I footprinting the ArgR binding site that controls the *argCJBDR* operon of *S. clavuligerus*. This operator is formed by two 18 nucleotides ARG boxes separated by 2 bp (6). This arrangement is conserved in ArgR putative binding sites of other *Streptomyces* promoter and in the upstream sequences of *S. coelicolor* genes (*argC*, *argG*, *arcB* and *argH*) involved in arginine biosynthesis.

To fit with the experimental transcriptomic and gel-shift data presented in this work a new ArgR binding site model has been designed (Fig. 10C). This sequence is formed by a 20 nucleotide-single ARG box and permitted the location of new ARG boxes with different *R*i values. The presence of two tandem ARG boxes in the arginine operon genes suggests that these genes are more tightly controlled than others containing a single ARG box. In some of the DNA fragments tested by EMSA two separated ARG boxes were predicted (Table 2). In the intergenic *malE*–*malR* region the two putative boxes with *R*i values of 9.3 and 8.6 are separated 83 nucleotides. The upstream region of *nrdA* and the intergenic SCO7302–SCO7303 or SCO2055–*hisD* regions also presented two putative sites with lower *R*i. Additional studies will be necessary to demonstrate whether or not ArgR binds both or only one of these ARG boxes.

In most of the cases shown in Fig. 10, the gel shift correlated with a statistically significant differential expression of the gene whose promoter was tested, indicating that the model 2 is functional, although additional EMSA should be done with increasing amounts of protein to determine the different ArgR affinity to the operators. However, this was not always the case, as occurred with the *amtB*, SCO3067–*sig15* or SCO2686 probes. This apparent lack of correlation in a few cases might be explained by the specific experimental conditions used in this study. The differential transcription observed in the microarray reflects the sampling of a 32-h culture grown in MG medium; however, in other media or culture times, these genes giving only *in vitro* binding might also show *in vivo* significant differential expression.

Gel-shift assays confirmed that the ArgR-dependent luciferase activity of these promoters results from the direct control of ArgR on these regions. As shown in Fig. 10 and Table 2, ArgR is a transcriptional regulator of pyrimidines (*pyrR*, *pyrA*, *pyrB*), nucleotides (i.e. SCO2015, *nrdA*), several amino acids (SCO4293), nitrogen metabolism (*amtB, ureA*) and several regulators (*whiB*, *rstP*, SCO2686 and SCO3979). ArgR also bound the intergenic region SCO3067–SCO3068 (for anti-anti-sigma and sigma factors) and the upstream region of SCO7314 encoding a sigma factor suggesting that many of the observed transcriptional effects are likely to be due to a cascade mechanism promoted by ArgR.

Regarding nitrogen control, putative ARG boxes were observed upstream of *amtB* (*R*i 8.3), upstream of *glnII* (into the SCO2209 coding region, *R*i 5.3) and in the 3′ end of *glnR* (*R*i 7.4). However, only the one located upstream of *amtB* was controlled by ArgR, as indicated by the luciferase assay and EMSA. Therefore, ArgR did not regulate *glnR* and the down-expression of *glnII* observed in the deleted mutant was not directly due to ArgR. Interestingly the *amtB* operator also binds GlnR but this regulatory protein and ArgR have different binding sites [50].

## Methods

### Culture conditions

Growth and manipulation of *Streptomyces* strains were carried out according to standard procedures [51]. Spore suspensions were obtained in TBO medium: 20 g/L tomato paste, 20 g/L oat flakes, and 20 g/L agar, pH 6.5. *S. coelicolor* cultures were grown at 30°C, 300 rpm (2.5 cm orbit), in starch and glutamate defined MG medium [16] that contained 2.5 mM potassium phosphate and 25 mM arginine when indicated. Baffled flasks (500 mL), that contained 100 mL of MG medium, were inoculated with 10^6^ spores/mL. Dry weight was determined in 2-mL culture samples that were washed twice with MilliQ water and dried at 65°C for 4 days. Arginine consumption was followed using the method of Hess *et al.* [52]. Cultures of plasmid-bearing cells were supplemented with ampicillin (50 µg/mL), chloramphenicol (25 µg/mL), kanamycin (25 µg/mL) or apramycin (50 µg/mL), as appropriate. *E. coli* DH5α was used as the general cloning host.

Cultures of 32 h were selected for the expression analysis due to the intense growth and good expression of amino acid biosynthesis pathways at this culture time. The experimental conditions chosen were as follows: (A) *S. coelicolor* M145 grown in MG medium, (B) *S. coelicolor* M145 grown in MG medium supplemented with 25 mM arginine, (C) *S. coelicolor* Δ*argR* grown in MG medium and (D) *S. coelicolor* Δ*argR* grown in MG medium supplemented with 25 mM arginine.

### Construction of a mutant of *S. coelicolor* with *argR* gene deletion

An *argR*-deleted mutant was constructed by PCR targeting [53] using oligonucleotides Coe-argR1 and Coe-argR2 on plasmid pTC123-*aphII,* a pTC182-derived plasmid [54] that contained the *neo* gene and a 5.7-kb SphI DNA insert carrying *S. coelicolor argBDR* genes as well as upstream and downstream sequences. After conjugation, four apramycin-resistant, kanamycin-sensitive *S. coelicolor* recombinants were characterized by Southern hybridization. We used as probes a SalI–PauI DNA fragment containing *argR* and the flanking sequences, and the apramycin resistance *acc(3)IV* gene. The hybridization pattern obtained confirmed the *argR* replacement deletion in the four identical clones that were named *S. coelicolor* Δ*argR.*

### Nucleic acid isolation and purification

Samples (2 mL) from 32-h cultures of *S. coelicolor* M145 and *S. coelicolor* Δ*argR* grown in MG medium, with or without arginine, were stabilized with RNA Protect Bacteria Reagent (Qiagen). For RNA isolation, mycelia were treated with lysozyme (30 mg/mL); the lysates were extracted with phenol and then transferred to RNeasy Midi Spin Columns (Qiagen), according to the manufacturer's instructions. RNA preparations were incubated with Turbo DNase (Ambion) to eliminate chromosomal DNA contamination. Sample quantification was done with a NanoDrop ND-1000 UV-Vis Spectrophotometer. Total genomic DNA (gDNA) was isolated from a stationary-phase culture following the Kirby mix procedure [51].

### PCR and semi-quantitative RT-PCR analysis

Oligonucleotide primers used in this study are shown in Table S2. All PCRs were performed in a TGradient (Biometra) thermocycler using Platinum Pfx DNA Polymerase (Invitrogen). The dNTP mix contained a higher proportion of G-C (35% each) than A-T nucleotides (15% each), to improve the amplification of high G+C DNA content. PCR products subcloned into pBluescript II SK(+) were sequenced to check the amplification fidelity. Gene expression analysis by RT-PCR was done with the SuperScript One-Step System (Invitrogen) using 150 ng total RNA as a template. For semi-quantitative analysis, samples were taken at three-cycle intervals between cycles 24 and 33, to compare non-saturated PCR product formation. Negative controls were carried out with each set of primers and Platinum *Taq* DNA polymerase (Invitrogen) to verify the absence of contaminating DNA in the RNA preparations.

### Purification of Strep-tagged ArgR

To purify the ArgR protein, plasmid pET-Strep-*argR* was constructed. The *argR* gene was amplified by PCR using oligonucleotides ArgR17/18 to place the Strep tag upstream of *argR*. The amplified product was subcloned in NdeI/HindIII digested plasmid pET-24a(+) (Novagen, Merck). The *E. coli* BL21(DE3)pLysS (Invitrogen) transformants carrying pET-Strep-*argR* were grown at 37°C to OD 0.6, induced with 1 mM IPTG, and the growth was continued at 20°C. After 18–20 h, the cells were harvested by centrifugation at 2640 rcf and kept at −80°C. Cells were broken with a Misonix XL-2000 sonifier, centrifuged at 16 100 rcf, and the supernatant was applied to a 1-mL StrepTrap HP column (GE Healthcare) and purified in an Akta Prime FPLC Protein Purification System (GE Healthcare) following the manufacturer's instructions.

### Electrophoresis mobility shift assay (EMSA)

The promoters cloned in pBluescript SK+ were sequenced and amplified by PCR using specific or universal 6-FAM labelled oligonucleotides (Table S2). The amplification products were used for EMSA as follows: the reaction contained 5 µL buffer (10 mM Tris–HCl, pH 7.4, 5 mM MgCl_2_, 2.5 mM CaCl_2_, 250 mM KCl, 0.5 mM DTT, 10 mM L-arginine, pH 7.4), poly-(dIdC) 1,3 µg/mL, 6-FAM-labelled probe 2 nM, glycerol 10% and Strep-ArgR protein 0.8 µM in a total volume of 15 µL. The reaction was maintained for 30 min at 30°C, and then the DNA was separated in a 5% acrylamide gel using 0.5× TBE as developing buffer at 50 V. The bands were visualized in an Ettan DIGE imager (GE Healthcare). In all cases, competition and specificity experiments were done with increasing amounts of unlabelled specific probe and with BSA. The *argH* promoter-probe was used to test the effect of L-arginine. ArgR affinity was higher in the presence of L-arginine, so it was mantained in the binding reaction mixture.

### Luciferase assay

For luciferase reporter analysis, promoter regions were amplified with primers containing NdeI and BamHI restriction sites (Table S2) to clone the promoters into the ATG codon of the *luxA* gene in pLUXAR-neo [55]. Cultures of *S. coelicolor* exconjugants harbouring the promoter–probe constructs were carried out in MG medium. Samples at 32, 47, 55 and 79 h were taken, spun down, kept frozen and processed simultaneously. Riboflavine was added to the cell suspension to improve the sensitivity of the luciferase assays, which were measured in a Luminoskan luminometer (Labsystems) [18], [55]. At least two different cultures from the same strain were analyzed and measured by duplicate.

### Labelling and microarray hybridizations

*S. coelicolor* microarrays (SCo3 design) were obtained from the Functional Genomics Laboratory, Surrey University (UK). They contained duplicated probes (50-mer) for 7728 chromosomal genes (out of 7825). The experimental design used *S. coelicolor* M145 gDNA as a common reference. RNA was extracted from two nutritional states, MG and MG with 25 mM arginine, and from two strains, *S. coelicolor* M145 and *S. coelicolor* Δ*argR*. Four biological replicates were made for each condition. The Pronto! Universal Microarray Hybridization kit (Corning) was used for prehybridization of the slides. Labelling reactions were performed according to the recommendations described in http://www.surrey.ac.uk/SBMS/Fgenomics. Total RNA was labelled with Cy3-dCTP (Amersham) using random primers and Superscript II reverse transcriptase (Invitrogen). gDNA was labelled with Cy5-dCTP (Amersham) from random primers extended with the Klenow fragment of DNA polymerase (Roche). The final products were purified with MinElute columns (Qiagen) and labelling efficiencies were quantified spectrophotometrically. Cy3-cDNA (100 pmol) and Cy5-labeled gDNA (20 pmol) were mixed, vacuum dried and resuspended in 40 µL Pronto! Long Oligo/cDNA Hybridization Solution (Corning), to be applied on the microarray surface. Hybridizations were carried out at 42°C and extended to 72 h to improve the quality of the results [56]. Washing, scanning with an Agilent DNA Microarray Scanner G2565BA, and image quantification were carried out as indicated previously [16].

### Identification of differentially transcribed genes and transcription profile classification

Microarray data were normalized and analysed with the Bioconductor package limma [57], [58]. Spot quality weights were estimated as indicated in Text S1. Local and global normalizations were both used [59]. First, weighted medians of log_2_ Cy3/Cy5 intensities were calculated for print-tip correction, and afterwards, global Loess was applied [57]. The normalized log_2_ of Cy3/Cy5 intensities is referred to in this work as the M*_g_* value, which is proportional to the abundance of transcripts for a particular gene [60]. The information from within-array spot duplicates [61] and empirical array weights [62] were taken into account in the linear models [58]. The transcription results of the four experimental conditions were compared using five contrasts. For each contrast, *p*-values and M*_c_* values (log measure of the differential transcription) were calculated. False-discovery rate (FDR) correction for multiple testing was applied. For each contrast or comparison between two experimental conditions, a result was considered as statistically significant if the FDR-corrected *p*-value was <0.05. A total of 459 genes showed statistically significant results in at least one contrast.

To classify the transcription profiles observed for this set of genes, we used the H*_r_* values, which summarized the results of the hypothesis tests. For each contrast and each gene, the H*_ri_* value of each gene was calculated as follows: (i) H*_ri_* = 0 indicated that the contrast result was not significant using uncorrected *p*-values (α = 0.05); and (ii) if the uncorrected *p*-value was <0.05, then H*_ri_* = 1 (indicating upregulation) if the respective M*_c_* value was positive, or H*_ri_* = −1, for a negative M*_c_* value (downregulation). The set of 459 genes yielded a total of 52 observed combinations of their H*_r_* values. Visual inspection of the transcription profiles allowed us to group a subset of 365 genes, which showed the profiles with more likely biological meaning, into 5 main types (I–V) and 13 subtypes as shown in Fig. 3 (see also Dataset S1 and Text S1).

### Bioinformatic analysis of ArgR binding sites

ArgR binding sites composed of two palindrome sequences, known as ARG boxes, have been identified previously in *S. clavuligerus* [6], [7]. ARG boxes were easily identified upstream of the arginine biosynthesis genes *argH*, *argG*, *argC* and *arcB* of *S. coelicolor*. The sequences of ARG boxes and their complementaries –due to the symmetry of the palindromic site– were used to create information theory models by the Delila programs makebk, encode, rseq, dalvec, ri and makelogo [63], [64]. To find new operator sites, the promoter regions (−300, +100 nt) of the *S. coelicolor* chromosome were scanned by means of the Patser algorithm and the RSA tools server [65].

### Proteomic

The mycelia from 20-mL culture samples of *S. coelicolor* M145 and *S. coelicolor* Δ*argR* grown for 32 h in MG medium were harvested by centrifugation (10 min at 2640 rcf), immediately frozen in liquid nitrogen, and stored at −80°C until use. Proteomic analysis and Mass Spectrometry identification were done as described by Santamarta *et al*. [66]. Positive identifications were based on the MOWSE score algorithm. A MOWSE score of 52 or higher was significant at the 5% level or better, and proteins typically gave scores well above 70. The analysis was performed in the Proteomic Service of the National Center for Biotechnology (Madrid, Spain).

## Supporting Information

Table S1 **Selected differentially expressed I and II profile genes.**
(DOC)

Table S2 **Primers used within this manuscript.**
(DOC)

Text S1 **Estimation of spot weights for microarray data analysis.**
(DOC)

Dataset S1 **Hypothesis-testing results for profile classification.**
(XLS)

Dataset S2 **Peptide mass fingerprints.**
(PDF)
