# Supplementary material for: ArgR of Streptomyces coelicolor Is a Versatile Regulator
Source: PLoS One. 2012 Mar 5;7(3):e32697. doi: 10.1371/journal.pone.0032697 (PMC3293853; doi:10.1371/journal.pone.0032697)
Supplement: Table S1 — Selected differentially expressed I and II profile genes. (DOC) [file pone.0032697.s001.doc]

**Table S1**

| **Type I genes** |  | |
| --- | --- | --- |
| **Subtypes I.1–I.3** | **TetR, DeoR, AraC, GntR or LipR-type of regulators** | SCO0337, SCO0428, *abaA* (SCO0703), *lipR* (SCO0712), SCO1636, SCO2182, SCO2094, SCO3979, SCO4920, *nrdS* (SCO5224), SCO5418, SCO5622, SCO5678 and SCO7610 |
|  | **sigma factors** | *sigR* (SCO5216) and *bldN* (SCO3323) |
|  | **anti-sigma factor~~s~~** | SCO5244 |
|  | **anti-anti sigma factor** | SCO4027 |
|  | **proteases, peptidases or aminotransferases** | SCO1676, SCO1865, SCO2074, SCO2617, SCO3168, SCO4752 and SCO4883 |
|  | **adenosine deaminases** | SCO5662 |
|  | **glutamyl-tRNA-charging amidotransferases** | *gatA* (SCO5499) and *gatB* (SCO5501) |
|  | **sporulation factors** | *whiH* (SCO5819) and *whiB* (SCO3034) |
| **Subtypes I.4–I.5** | **aerial hyphae formation** | *chpC* (SCO1674), *chpH* (SCO1675), *chpE* (SCO1800), *chpG* (SCO2699) and  *rdlA* (SCO2718) |
| **Type II genes** |  | |
| **Subtypes II.2–II.3** | **AsnC, AraC, PadR, AbaA, TetR or DeoR-type of regulators** | SCO0310, SCO0659, SCO0745, SCO1119, SCO3335, SCO3522, SCO3900, SCO4214, *cpkO* (SCO6280) and SCO6865 |
|  | **two component systems** | SCO2143, SCO3750, SCO3756 and SCO4009 |
|  | **proteases, peptidases and protease inhibitors** | SCO0762, SCO2549, SCO5149, SCO5795, SCO6076 and SCO7521 |
