# Supplementary material for: ArgR of Streptomyces coelicolor Is a Versatile Regulator
Source: PLoS One. 2012 Mar 5;7(3):e32697. doi: 10.1371/journal.pone.0032697 (PMC3293853; doi:10.1371/journal.pone.0032697)
Supplement: Table S2 — Primers used within this manuscript. (DOC) [file pone.0032697.s002.doc]

**Table S2**

| **Primer Name** | | **Sequence (5´🡪3´) (1)** | **Restriction site** | **Use (2)** |  |
| --- | --- | --- | --- | --- | --- |
| Coe-argR1 | | CCCACGGGGACGGACGATCCGGAGAATGAGACGACGATGattccggggatccgtcgacc |  | FP for PCR-targeted *argR* disruption |  |
| Coe-argR2 | | CGTGCCCGCGGTCGGCAGATCACCGCACCGCCCCCGTCAtgtaggctggagctgcttc |  | RP for PCR-targeted *argR* disruption |  |
| ArgR17 | | GGGGAATTCCATATGTGGAGCCACCCGCAGTTCGAAAAGAGCCACGCGCAGGAGCACGAGCAGC | NdeI | FP for the construction of plasmid pET-Strep-*argR* |  |
| ArgR18 | | Cggcggaagctttgaccaggagcgcggggaac | HindIII | RP for the construction of plasmid pET-Strep-*argR* |  |
| 6FAM-Direct | | CGACGTTGTAAAACGACGGCCAGT |  | FP to amplify 6-FAM labelled probes for EMSA |  |
| 6FAM-Reverse | | CAGGAAACAGCTATGAC |  | RP to amplify 6-FAM labelled probes for EMSA |  |
| CRP13 | | cccaggatccgccgtctatgcgctcataag | BamHI | FP for coupling *argH* promoter to the luciferase RS and EMSA |  |
| CRP14 | | Tgctcatatgggtcgctcctcaccgtacgatc | NdeI | RP for coupling *argH* promoter to the luciferase RS and EMSA |  |
| CRP17 | | GATGGTCATATGGAGCACTCCCTCGCTCGGCACGG | NdeI | RP for coupling *nrdA* promoter to the luciferase RS and EMSA |  |
| CRP18 | | GGGCGGATCCCTGTCGACGCAGGCCGCCAT | BamHI | FP for coupling *nrdA* promoter to the luciferase RS and EMSA |  |
| CRP19 | | CGATCATATGTCAGTCAATGACTTTTCAGGTG | NdeI | RP for SCO1086 promoter cloning and EMSA |  |
| CRP20 | | GCAGGGATCCTTCCGCTTCTACTTCTGGGA | BamHI | FP for SCO1086 promoter cloning and EMSA |  |
| CRP21 | | GATCATATGACCAATTCTCTCAGACCCGCG | NdeI | RP for coupling *hisD* promoter to luciferase RS and test intergenic- *hisD*-SCO2055 by EMSA |  |
| CRP22 | | GCGGATCCCTTCGGTCACGGTGCTTC | BamHI | FP for coupling *hisD* promoter to luciferase RS and test intergenic- *hisD*-SCO2055 by EMSA |  |
| CRP23 | | CGGTCATATGCGCCCCTCGTCTGTCTTTCG | NdeI | RP for coupling *whiB* promoter to the luciferase RS and EMSA |  |
| CRP24 | | GCGGATCCGCGTGTATCGGAACGGGATC | BamHI | FP for coupling *whiB* promoter to the luciferase RS and EMSA |  |
| CRP25 | | GCTGCATATGTCAGGCTCCGTAAGGCGATTC | NdeI | RP for coupling *pyrB* promoter to the luciferase RS and EMSA |  |
| CRP26 | | GCGGATCCTACGTCGGCAAGAACCTC | BamHI | FP for coupling *pyrB* promoter to the luciferase RS and EMSA |  |
| CRP27 | | CGATCATATGGACGGACTGGATATCGGTGCG | NdeI | RP for *pyrA* promoter cloning and EMSA |  |
| CRP28 | | CCGGATCCGGCACCTTCAAGCTGAAG | BamHI | FP for *pyrA* promoter cloning and EMSA |  |
| CRP29 | | CACCCATATGATGTCGGGCCCTTCGAGAAC | NdeI | RP for coupling *pyrR* promoter to the luciferase RS and EMSA |  |
| CRP30 | | GCGGATCCTATGGCTCCCCGGACAAG | BamHI | FP for coupling *pyrR* promoter to the luciferase RS and EMSA |  |
| CRP31 | | GTTGGCCATATGGGCGTGGGCTCCTCGTGATGTC | NdeI | RP for coupling *leuA* promoter to the luciferase RS and EMSA |  |
| CRP32 | | GTGGATCCACTGTCACTACCCGCAAC | BamHI | FP for coupling *leuA* promoter to the luciferase RS and EMSA |  |
| CRP33 | | GGCGCATATGTCGAGCCTATGACTTACTGCCGGGTAG | NdeI | RP for coupling *pyk1* promoter to the luciferase RS and test intergenic region *pyk1*-SCO2015 by EMSA |  |
| CRP34 | | CGGGATCCCAACGGCATGGCTGATTC | BamHI | FP for coupling *pyk1* promoter to the luciferase RS and test intergenic region *pyk1*-SCO2015 by EMSA |  |
| CRP35 | | CCGAATTCTGACCCGCGTTGGAGTTG | EcoRI | RP for *argC* promoter cloning and EMSA |  |
| CRP36 | | TCGGATCCACGACCAGCTCGACGATC | BamHI | FP for *argC* promoter cloning and EMSA |  |
| CRP37 | | CCGAATTCATGTCGGGCTCGTCGTAC | EcoRI | RP for *argG* promoter cloning and EMSA | |
| CRP38 | | CGGGATCCGATCGAGGAGTTCCTGGAGTAC | BamHI | FP for *argG* promoter cloning and EMSA | |
| CRP39 | | CCGCCATATGGGCGCTCCAGAGGTGCAGGTC | NdeI | RP for coupling *arcB* promoter to the luciferase RS and EMSA | |
| CRP40 | | CCGGATCCAACAGGGCATCGAGGTGA | BamHI | FP for coupling *arcB* promoter to the luciferase RS and EMSA | |
| CRP41 | CCGGATCCGTCGGGCGACGTGAACGTAAC | | BamHI | FP for *malE-malR* promoter cloning and EMSA | |
| CRP42 | TGGTCATATGGGTGGACTGTACCTGTCGGCC | | NdeI | RP for *malE-malR* promoter cloning and EMSA | |
| CRP43 | ccggatccccgaggatgttcgctttg | | BamHI | FP for SCO5864 promoter cloning and EMSA | |
| CRP44 | ttgccatatgcgctctccccctctgggtgtc | | NdeI | RP for SCO5864 promoter cloning and EMSA | |
| CRP45 | tgctgaagcttgccgatagtagggctcatgg | | HindIII | RP for *rstP* promoter cloning and EMSA | |
| CRP46 | cgggatccgtgagttgagacctcttc | | BamHI | FP for *rstP* promoter cloning and EMSA | |
| CRP47 | Tcggatcctcaaccgtcctggccttc | | BamHI | FP for SCO4293 promoter cloning and EMSA | |
| CRP48 | cagtcatatggctcctcctatcagaggtcgacggcggcgtcggtg | | NdeI | RP for SCO4293 promoter cloning and EMSA | |
| FAM-CRP51 | GATCGTCGCAGAGGCGGAAG | |  | FP to amplify 6-FAM labelled ARG-box in SCO2209 for EMSA | |
| CRP52 | ACCTCCACGGTGACCCTGCG | |  | RP to amplify 6-FAM labelled ARG-box in SCO2209 for EMSA | |
| FAM-CRP53 | GAGGTGGTGCGTGGTCATG | |  | FP to amplify 6-FAM labelled SCO7302-7303 promoter for EMSA | |
| CRP54 | TCGCAGACCTCCTCGATC | |  | RP to amplify 6-FAM labelled SCO7302-7303 promoter for EMSA | |
| FAM-CRP55 | TACCCCAGTTCCTTGATC | |  | FP to amplify 6-FAM labelled ARG-box in *glnR* for EMSA | |
| CRP56 | GGCGAGAAGAGCGATAAG | |  | RP to amplify 6-FAM labelled ARG-box in *glnR* for EMSA | |
| FAM-CRP57 | GAGGGCAACTCGACCGGATG | |  | FP to amplify 6-FAM labelled SCO3979 promoter for EMSA | |
| CRP58 | ATGCCCACCGCGTCGTAC | |  | RP to amplify 6-FAM labelled SCO3979 promoter for EMSA | |
| FAM-CRP59 | GGGCATCTGGATGTCGAGGAAG | |  | FP to amplify 6-FAM labelled SCO1220-1221 promoter for EMSA | |
| CRP60 | TCCGATCTCCGCGCCGATC | |  | RP to amplify 6-FAM labelled SCO1220-1221 promoter for EMSA | |
| CRP65 | gcatcaccgtggtcgttc | |  | FP for SCO7314 promoter cloning and EMSA | |
| CRP66 | CCATGCCGTGACGAGTTC | |  | RP for SCO7314 promoter cloning and EMSA | |
| CRP69 | TCCCGTACCTCGACCAGAAG | |  | FP for SCO3067-*sig15* promoter cloning and EMSA | |
| CRP70 | AGCCGGGGTGACATGATG | |  | RP for SCO3067-*sig15* promoter cloning and EMSA | |
| CRP71 | gaagacccaggctcgtcaag | |  | FP for SCO2686 promoter cloning and EMSA | |
| CRP72 | AAAGGCGCGGTCTTGAAC | |  | RP for SCO2686 promoter cloning and EMSA | |
| CRP73 | | cgtaggggctctccgagaac |  | FP for SCO0800-0801 promoter cloning and EMSA |  |
| CRP74 | | TCGCTCCCACGACGTCTC |  | RP for SCO0800-0801 promoter cloning and EMSA |  |
| CAR63 | | CCGTGGATCCGGCCGTACGCGATTTC | BamHI | FP for coupling *amtB* promoter to the luciferase RS and EMSA |  |
| CAR64 | | CTGGTACCATATGCGTCTCCTCGTCGTTG | NdeI | RP for coupling *amtB* promoter to the luciferase RS and EMSA |  |
| PHO-49 | | GAGCATCACCGCTTCGGCAT |  | RP for *ureA* promoter cloning and EMSA |  |
| PHO-50 | | CGTGCGGGGTCAGTTGCATT |  | FP for *ureA* promoter cloning and EMSA |  |
| RT-argC-1 | | GGTGGCCGGAGCGAGTGGGTATG |  | FP for *argC* RT-PCR analysis |  |
| RT-argC-2 | | CAGGGAGACGGCCGTGGGGTAGCA |  | RP for *argC* RT-PCR analysis |  |
| RT-argR-3 | | ACACCGACGGCGACCTCATCTACG |  | FP for *argR* RT-PCR analysis |  |
| RT-argR-4 | | TGGGCCAAGCGCAGCAAGTG |  | RP for *argR* RT-PCR analysis |  |
| RT-argH-5 | | GGATGATCGCCGGACTGGAC |  | FP for *argH* RT-PCR analysis |  |
| RT-argH-6 | | CCGGCTGGGCGTGCTGGAGGTG |  | RP for *argH* RT-PCR analysis |  |
| RT-argG-7 | | GCGGGCGGGCGTACTTCAACACCA |  | FP for *argG* RT-PCR analysis |  |
| RT-argG-8 | | ATGCCGGGCGCCTCGTAGATGC |  | RP for *argG* RT-PCR analysis |  |
| RT-pyrR-9 | | GGAGGTTCTTGCCGACGTAG |  | FP for *pyrR* RT-PCR analysis |  |
| RT-pyrR-10B | | GCTGGAGCAGATCACCGAAC |  | RP for *pyrR* RT-PCR analysis |  |
| RT-pyr1485-11 | | CCTTCCGTTTCCGTCTTGTTG |  | FP for SCO1485 RT-PCR analysis |  |
| RT-pyr1485-12B | | TCTTCGTCGCGCTCGTCTAC |  | RP for SCO1485 RT-PCR analysis |  |
| RT-pyrA-13 | | TGTCGTTCTTGTCGCGCATC |  | FP for *pyrA* RT-PCR analysis |  |
| RT-pyrA-14B | | TCATGACCGACCCGGAGATC |  | RP for *pyrA* RT-PCR analysis |  |
| RT-scbR-19 | | GCAGGTCTTCGAGAAGCAGGGCTAC |  | FP for *scbR* RT-PCR analysis |  |
| RT-scbR-20B | | TGACCACATGGGGCAGCAAC |  | RP for *scbR* RT-PCR analysis |  |
| RT-rdlA-23 | | CGGCTGCCGCTTCTGTGATC |  | FP for *rdlA* RT-PCR analysis |  |
| RT-rdlA-24B | | GGATCGGCACGAGGTTGATG |  | RP for *rdlA* RT-PCR analysis |  |
| RT-chpE-27 | | AGGCAGCGGCCGTGACGATG |  | FP for *chpE* RT-PCR analysis |  |
| RT-chpE-28B | | TGACGCCCAGGTTGCCGAAG |  | RP for *chpE* RT-PCR analysis |  |
| RT-chpC-29 | | GCAAGGGCCTGATGACGATG |  | FP for *chpC* RT-PCR analysis |  |
| RT-chpC-30 | | TCGGCACCTCGACGTGGTTG |  | RP for *chpC* RT-PCR analysis |  |
| RT-cpkJ-31 | | TCGACACACCCGGCTACTAC |  | FP for *cpkJ* RT-PCR analysis |  |
| RT-cpkJ-32 | | CGGAGACCTTGCTCTCGAAG |  | RP for *cpkJ* RT-PCR analysis |  |
| RT-cpkI-33 | | TGACTGCCCTCACGAACAAG |  | FP for *cpkI* RT-PCR analysis |  |
| RT-cpkI-34 | | TGACACCGGAGGAGATGTTG |  | RP for *cpkI* RT-PCR analysis |  |
| RT-whiB-35 | | ATGACCGAGCTGGTGCAGCAAC |  | FP for *whiB* RT-PCR analysis |  |
| RT-whiB-36 | | TTCTTGAGACGGCGGCGTTC |  | RP for *whiB* RT-PCR analysis |  |
| RT-glnK-37 | | ACCGGCTCGACGAGATCAAG |  | FP for *glnK* RT-PCR analysis |  |
| RT-glnK-38 | | TCGCCGGTCCTCACCCGTAC |  | RP for *glnK* RT-PCR analysis |  |
| RT-glnII-39 | | CCAAGCTCCGTTCCAAGACGAAG |  | FP for *glnII* RT-PCR analysis |  |
| RT-glnII-40 | | GCATGACCTCGGCGTTGATG |  | RP for *glnII* RT-PCR analysis |  |
| RT-glnA-41 | | ACGCCGACGACGTCAAGAAG |  | FP for *glnA* RT-PCR analysis |  |
| RT-glnA-42 | | GGAAGTAGCCGCCCTTGTAG |  | RP for *glnA* RT-PCR analysis |  |

1. Deletion cassette homologous region of PCR targeting oligonucleotides or restriction sites added in the 5’ region of cloning oligonucleotides are underlined
2. Abreviations: FP= forward primer; RP = Reverse primer; RS = Reporter system
