# Supplementary material for: ArgR of Streptomyces coelicolor Is a Versatile Regulator
Source: PLoS One. 2012 Mar 5;7(3):e32697. doi: 10.1371/journal.pone.0032697 (PMC3293853; doi:10.1371/journal.pone.0032697)
Supplement: Text S1 — Estimation of spot weights for microarray data analysis. (DOC) [file pone.0032697.s003.doc]

**Text S1. Estimation of spot weights for microarray data analysis**

1. ***Quality flags***

The Feature Extraction software quantifies the spot fluorescence intensities and provides a set of Boolean values to assess the data quality for each spot. These quality indicators evaluate both red and green channel data of each spot (indicators are listed in the first row of the table below; see the software manual for complete descriptions). Based on previous observations of the significance of each quality indicator, we classified the indicated combinations of Boolean values into a unique quality flag (see table below). Although spots with the best quality results were flagged as "1.00", other flag values were arbitrary.

**Determination of quality flags for spot data**

1. ***Empirical estimation of spot weights***

To obtain spot quality weights that could be entered into the data analysis, we followed the idea behind the array weight estimation of Ritchie *et al*. (2006). In our case, the data of the 16 array hibridizations (4 conditions x 4 biological replicates) were normalized, the array weights calculated, and a linear model was fitted as indicated in Materials and Methods. In this initial stage, all spots were equally weighted irrespective of their quality flags.

Spots with more reproducible transcription values —less variable— between replicates indicate a higher data quality. Hence, means of spot variances for each flag group were calculated. Spot weights were simply obtained by the normalized inverse of the mean variance. The results are summarized in the table bellow.

**Estimated weights for each spot quality flag**
