# Supplementary material for: ArgR of Streptomyces coelicolor Is a Versatile Regulator
Source: PLoS One. 2012 Mar 5;7(3):e32697. doi: 10.1371/journal.pone.0032697 (PMC3293853; doi:10.1371/journal.pone.0032697)
Supplement: Dataset S2 — Peptide mass fingerprints. (PDF) [file pone.0032697.s005.pdf]

Peptide Mass Fingerprint for spot 6149: SCO0379

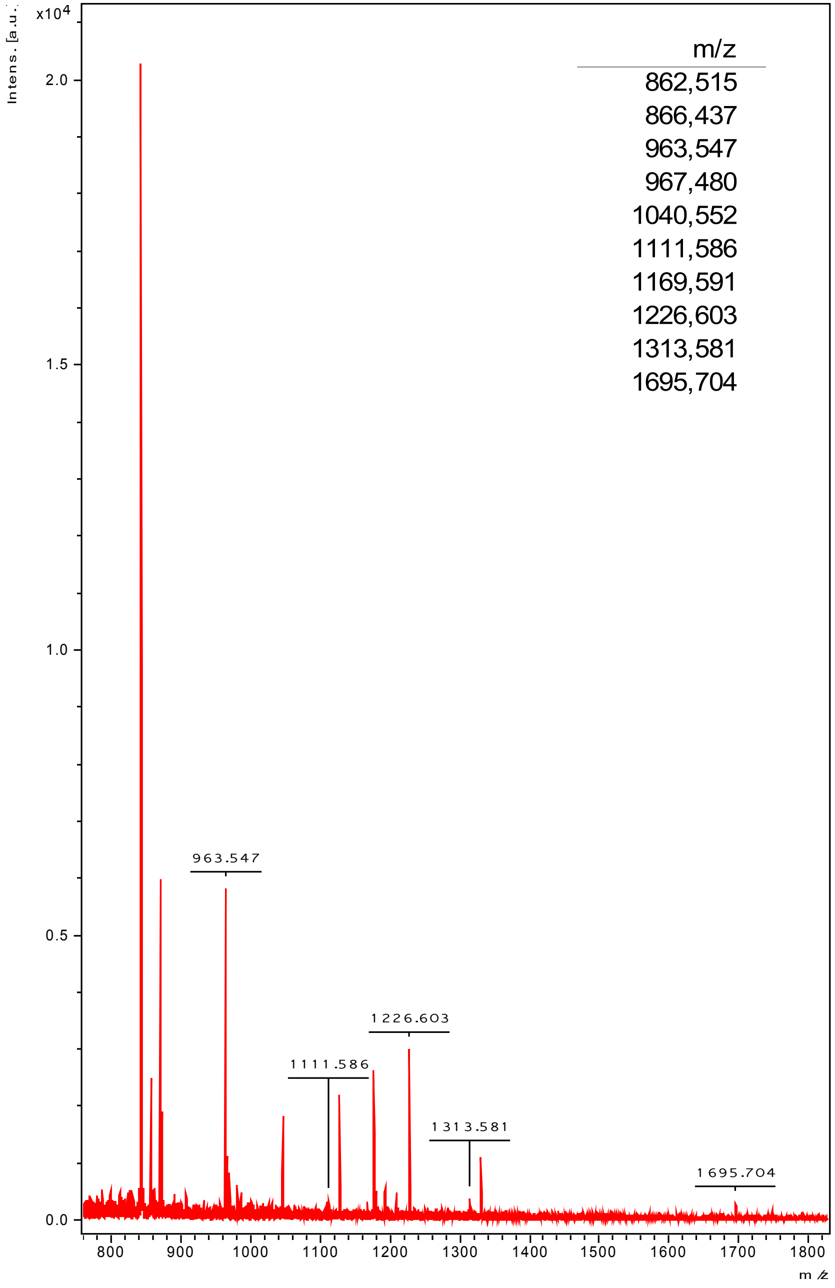

Mascot Search Results

User : irene
Email : isanh@unileon.es
Search title : spot 6149
Database : NCBInr 20100529 (11111565 sequences; 3786451707 residues)
Taxonomy : Streptomyces coelicolor (8575 sequences)
Timestamp : 4 Jun 2010 at 08:16:29 GMT
Top Score : 73 for gi|21218922, catalase [Streptomyces coelicolor A3(2)]

Mascot Score Histogram

Protein score is -10*Log(P), where P is the probability that the observed match is a random event.
Protein scores greater than 52 are significant (p<0.05).

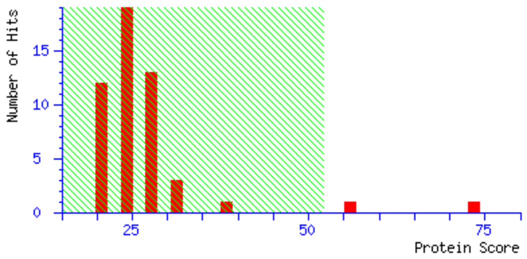

Index

	Accession	Mass	Score	Description
1.	gi|21218922	55139	73	catalase [Streptomyces coelicolor A3(2)]
2.	gi|2052219	55463	56	catalase [Streptomyces coelicolor]
3.	gi|21234037	35740	39	putative transposase [Streptomyces coelicolor A3(2)]
4.	gi|21225946	45243	30	cytochrome P450 [Streptomyces coelicolor A3(2)]
5.	gi|21220009	46650	30	histidyl-tRNA synthetase [Streptomyces coelicolor A3(2)]
6.	gi|21220698	194331	30	bi-functional protein (secreted alpha-amylase/dextrinase) [Streptomyces coelicolor A3(2)]
7.	gi|21218820	114800	29	hypothetical protein SCO0269 [Streptomyces coelicolor A3(2)]
8.	gi|21226044	10980	28	hypothetical protein SCO7792 [Streptomyces coelicolor A3(2)]
9.	gi|21225001	39963	28	ATP-dependent DNA ligase [Streptomyces coelicolor A3(2)]
10.	gi|21220329	57941	27	ABC transporter ATP binding protein [Streptomyces coelicolor A3(2)]

Sequence Coverage: 13%

Matched peptides shown in **Bold Red**

1 MPENNQKPLT TVAGAPVPDN QNSLTSGPRG PMLLQDVWFL EKLAHFDREV
51 IPERRMHAK**G** **SGAFGTFTVT** **HDITR**YTSAK IFSEIGK**KTP** **LFTRFSTVAG**
101 **ER**GAADAERD IRGFAVKFYT DEGNWDLVGN NTPVFFFRDP LK**FPDLNHAV**
151 **K**RDPRTNLRN AENNWDFWTN LPEALHQVTI VMSDRGIPAS YRHMHGFGSH
201 TYSLINAEGE RFWVKFHHRT QQGIKNLTDA EAEALVGKDR ESHQRDLFDA
251 IEDGDFPKWK LFIQVMPEAD AENYRFHPFD LTKVWSKKDY PLIEVGEWEL
301 NRNPDNYFAD VEQAAFSPAN VVPGISFSPD RMLQGRLFSY GDAQRYRLGV
351 NHHQIPVNAP KNPVNSYHRD GAMRVDGNQG ATPGVEPNSY GRWQEQPAYR
401 **DPAQAVGAVA** **DR**FNYREDDD NYFEQPGNLF RQMSPEQQQV LFENTAR**AID**
451 **GASAQTIER**H IGNCTQADPA YGAGVRKAIE ALAAGNL

Peptide Mass Fingerprint for spot 6577: SCO1570

m/z
917,520
985,497
1061,531
1089,557
1177,670
1183,643
1197,695
1485,814
1489,751
1525,845
2319,298

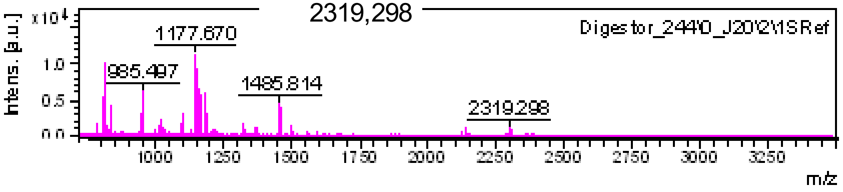

Mascot Search Results

User : irene
Email : isanh@unileon.es
Search title : spot 6577
Database : NCBInr 20100602 (11126451 sequences; 3793134231 residues)
Taxonomy : Streptomyces coelicolor (8575 sequences)
Timestamp : 8 Jun 2010 at 17:42:51 GMT
Top Score : 71 for gi|21220068, argininosuccinate lyase [Streptomyces coelicolor A3(2)]

Mascot Score Histogram

Protein score is -10*Log(P), where P is the probability that the observed match is a random event.
Protein scores greater than 52 are significant (p<0.05).

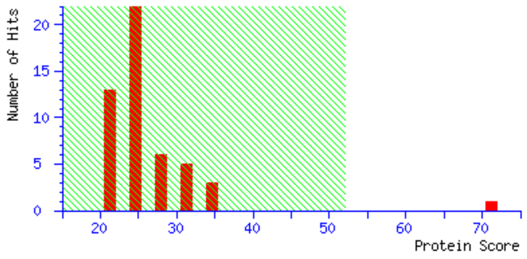

Index

	Accession	Mass	Score	Description
1.	gi|21220068	50967	71	argininosuccinate lyase [Streptomyces coelicolor A3(2)]
2.	gi|21225548	34681	34	ion channel subunit [Streptomyces coelicolor A3(2)]
3.	gi|21218772	28703	34	nitrate reductase delta chain NarJ2 [Streptomyces coelicolor A3(2)]
4.	gi|21219843	32751	33	oxidoreductase [Streptomyces coelicolor A3(2)]
5.	gi|21224934	38541	33	hypothetical protein SCO6637 [Streptomyces coelicolor A3(2)]
6.	gi|21234058	32616	32	putative DNA-binding protein [Streptomyces coelicolor A3(2)]
7.	gi|21223145	32307	32	transcriptional regulator [Streptomyces coelicolor A3(2)]
8.	gi|21234061	65898	31	putative atp/GTP-binding protein [Streptomyces coelicolor A3(2)]
9.	gi|21219988	35753	30	aspartate carbamoyltransferase catalytic subunit [Streptomyces coelicolor A3(2)]
10.	gi|21218824	55178	28	alpha-galactosidase SCF8502 [Streptomyces coelicolor A3(2)]

Sequence Coverage: 11%

Matched peptides shown in **Bold Red**

```
  1 MSSNSGDVRL WGGRFADGPA EALAKLSASV HFDWRLAPYD IAGSRAHARV
 51 LHAAGLLTED ELTRMIAGLD RLEADVADGS FTGTIADEDV HTALERGLLE
101 RLGPDLGGKL RAGRSRNDQV ATLFRMYLRD HARTVGSLIA DLQDALVGLA
151 EAHPDVAMPG RTHLQHAQPV LFAHHVLAHA QALGRDAERL RQWDARTAVS
201 PYGSGALAGS SLGLDPEAVA RDLGFEHGSV GNSIDGTASR DFVAEFAFIT
251 AMIGVNVSRI AEEIIIWNTK EFSFVTLHDA FSTGSSIMPQ KKNPDIAELA
301 RGKSGRLIGN LTGLMATLKA LPLAYNRDLQ EDKEPVFDSI DQLEVLLPAF
351 TGMMATLTVH RERMEELAPA GFSLATDIAE WLVKQGVPFR VAHEVAGECV
401 KVAEADGKEL DELTDEQFAK ISEHLTPEVR TVLNVPGALA SRDGRGGTAP
451 SAVAVQLAEV KADVAAQHAW ADAKK
```

Peptide Mass Fingerprint for spot 6655: SCO1580

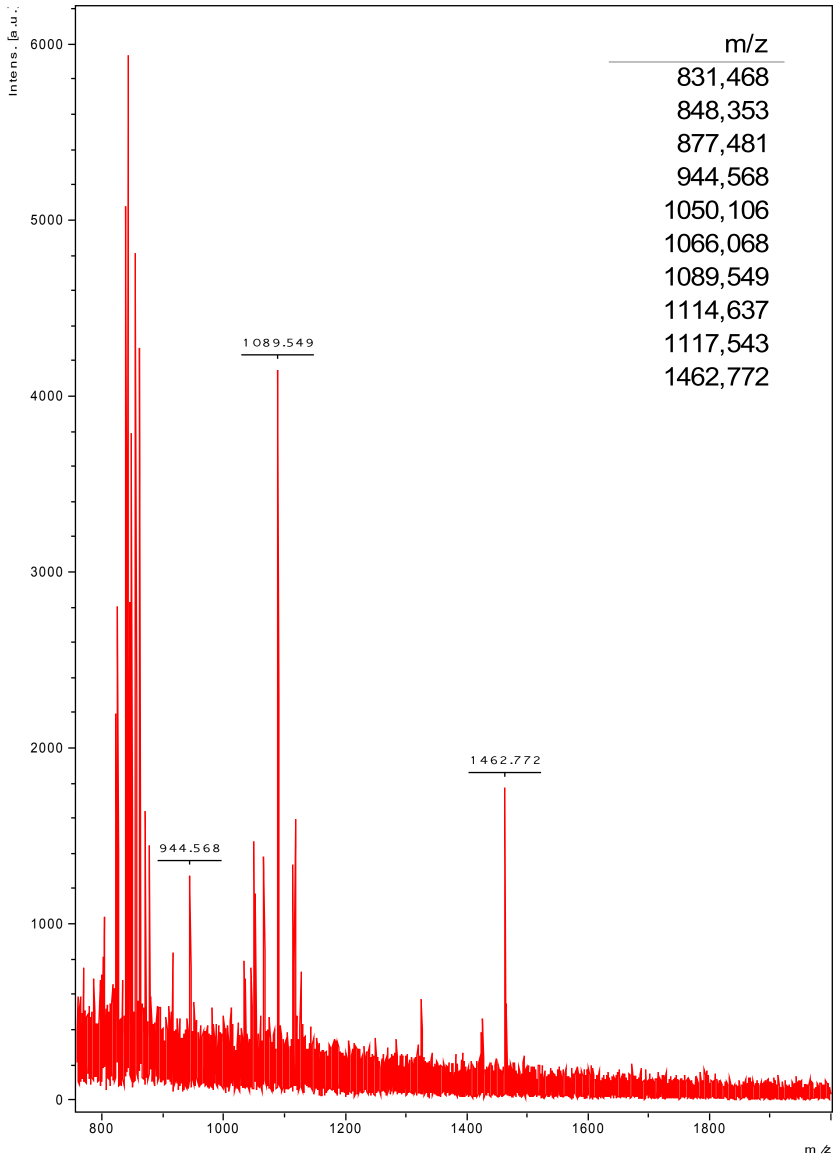

MATRIX SCIENCE **Mascot Search Results**

User : irene
Email : isanh@unileon.es
Search title : spot 6655
Database : NCBInr 20100529 (11111565 sequences; 3786451707 residues)
Taxonomy : Streptomyces coelicolor (8575 sequences)
Timestamp : 4 Jun 2010 at 09:03:29 GMT
Top Score : 82 for gi|21220077, N-acetyl-gamma-glutamyl-phosphate reductase [Streptomyces coelicolor A3(2)]

Mascot Score Histogram

Protein score is -10*Log(P), where P is the probability that the observed match is a random event.
Protein scores greater than 52 are significant (p<0.05).

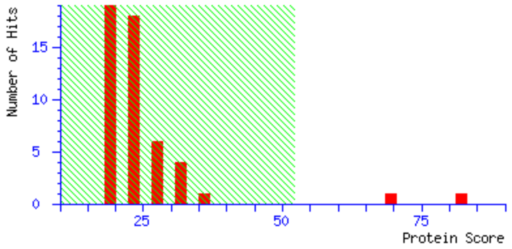

Index

	Accession	Mass	Score	Description
1.	gi|21220077	34708	82	N-acetyl-gamma-glutamyl-phosphate reductase [Streptomyces coelicolor A3(2)]
2.	gi|46824	25360	71	N-acetyl-gamma-glutamyl-phosphate reductase [Streptomyces coelicolor]
3.	gi|32141213	66287	38	molecular chaperone DnaK [Streptomyces coelicolor A3(2)]
4.	gi|21220801	5705	33	hypothetical protein SCO2333 [Streptomyces coelicolor A3(2)]
5.	gi|21224333	98902	31	hypothetical protein SCO5996 [Streptomyces coelicolor A3(2)]
6.	gi|21225060	40967	31	4-hydroxy-3-methylbut-2-en-1-yl diphosphate synthase [Streptomyces coelicolor A3(2)]
7.	gi|21224045	41169	31	4-hydroxy-3-methylbut-2-en-1-yl diphosphate synthase [Streptomyces coelicolor A3(2)]
8.	gi|21219150	48301	29	hypothetical protein SCO0618 [Streptomyces coelicolor A3(2)]
9.	gi|21218860	42898	28	acyl-CoA dehydrogenase [Streptomyces coelicolor A3(2)]
10.	gi|21220744	41758	27	lipoprotein [Streptomyces coelicolor A3(2)]

Sequence Coverage: **15%**

Matched peptides shown in **Bold Red**

1 MAVR**AAVAGA SGYAGGELLR** LLLTHPEVEI GALTGNSNAG QRLGALQPHL
51 LPLADRVLEA TTPEVLGGHD VVFLALPHGQ SAAVAEQLGP DVLVVDMGAD
101 FR**LKDAGDWE R**FYGSPHAGT WPYGLPELPG AR**AALEGSKR** IAVPGCYPTA
151 VSLALFPAYA ASLAEPEAVI VAASGTSGAG KAAKPHLLGS EVMGSMSPYG
201 VGGGHRHTPE MIQNLGAVAG EPVTVSFTPT LAPMPR**GILA TCTAKAKPGV**
251 **TAESVR**AAYE KALADEPFVH LLPEGQWPAT ASVYGSNAVQ VQVAHDAAAG
301 RIIAISAIDN LAKGTAGGAV QSMNLALGLD ETTGLTTIGV AP

Peptide Mass Fingerprint for spot 6550: SCO1814

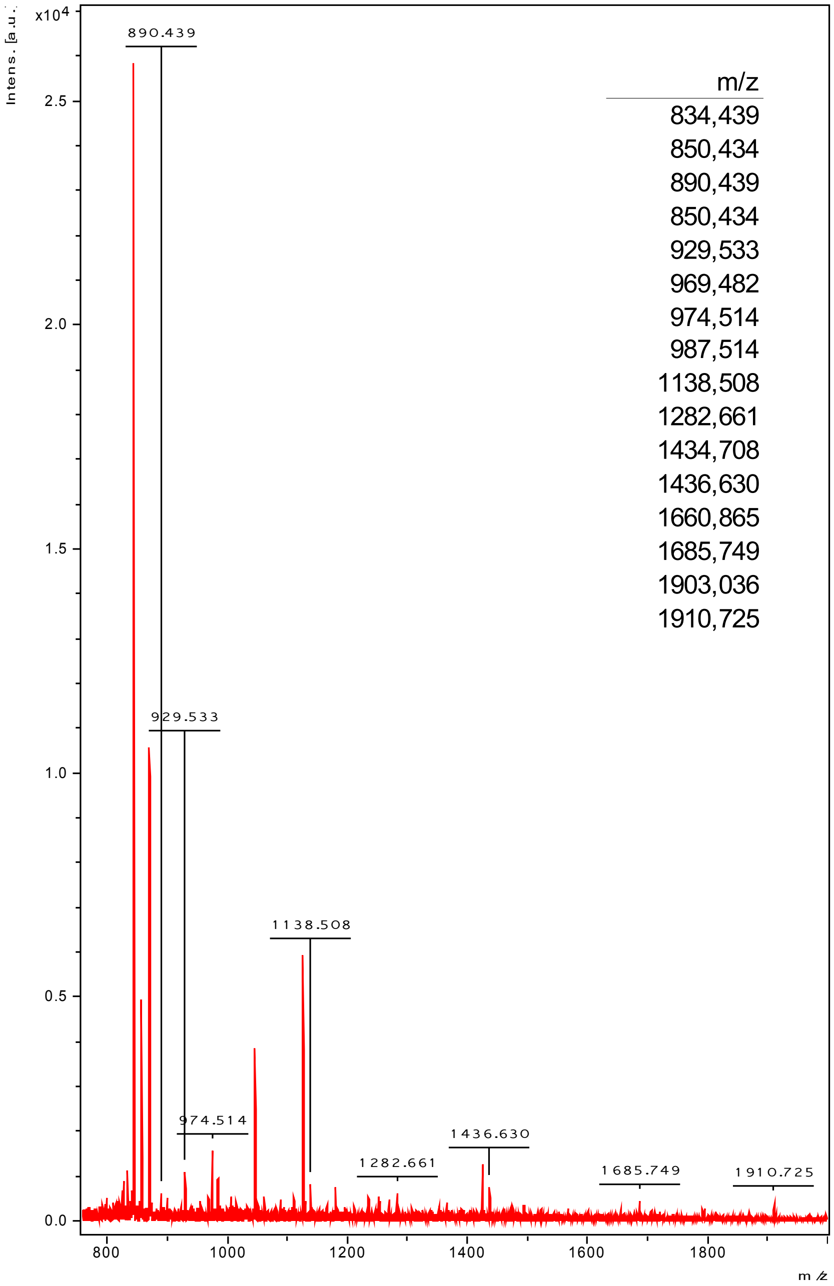

Mascot Search Results

```
User           : irene
Email          : isanh@unileon.es
Search title   : spot 6550
Database       : NCBInr 20100602 (11126451 sequences; 3793134231 residues)
Taxonomy       : Streptomyces coelicolor (8575 sequences)
Timestamp      : 8 Jun 2010 at 07:26:39 GMT
Top Score      : 64 for gi|21220304, enoyl-(acyl carrier protein) reductase [Streptomyces coelicolor A3(2)]
```

Mascot Score Histogram

Protein score is -10*Log(P), where P is the probability that the observed match is a random event.
Protein scores greater than 52 are significant (p<0.05).

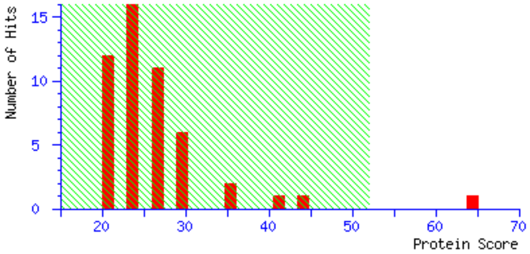

Index

	Accession	Mass	Score	Description
1.	gi|21220304	27250	64	enoyl-(acyl carrier protein) reductase [Streptomyces coelicolor A3(2)]
2.	gi|21223982	20774	44	ribosome recycling factor [Streptomyces coelicolor A3(2)]
3.	gi|21223255	34716	41	polysaccharide biosynthesis related protein [Streptomyces coelicolor A3(2)]
4.	gi|21223930	15952	35	hypothetical protein SCO5574 [Streptomyces coelicolor A3(2)]
5.	gi|21223426	56410	34	secreted protein [Streptomyces coelicolor A3(2)]
6.	gi|21220635	36900	30	kinase [Streptomyces coelicolor A3(2)]
7.	gi|21219464	31179	30	formamidopyrimidine-DNA glycosylase [Streptomyces coelicolor A3(2)]
8.	gi|21219445	31682	30	hypothetical protein SCO0926 [Streptomyces coelicolor A3(2)]
9.	gi|21224699	20339	29	hypothetical protein SCO6387 [Streptomyces coelicolor A3(2)]
10.	gi|21224661	145012	29	hypothetical protein SCO6348 [Streptomyces coelicolor A3(2)]

Sequence Coverage: 23%

Matched peptides shown in **Bold Red**

1 **MSGILEGK**R**V LITGVLMESS IAFHAAK**LAQ EQGAEIILTA FPRPTLTERI
51 AKKLPKPTKV IELDVTNDEH LARLADVVGE ELGGLDGVVH SIGFAPQDAL
101 GGNFLNTPFE SVATAMHVSA YSLKSLTMAC MPLMQNGGSV VGLTFDAQYA
151 WPQYDWMGPA KAALEATSR**Y MARDLGK**QNI RCNLVSAGPI GSMAAKSIPG
201 FSDLAAVWDS R**SPLEWDLK**D PEPAGRGIVA LLSDWFPK**TT GEIIHVDGGL**
251 **HAIGA**

Peptide Mass Fingerprint for spot 6445: SCO1935

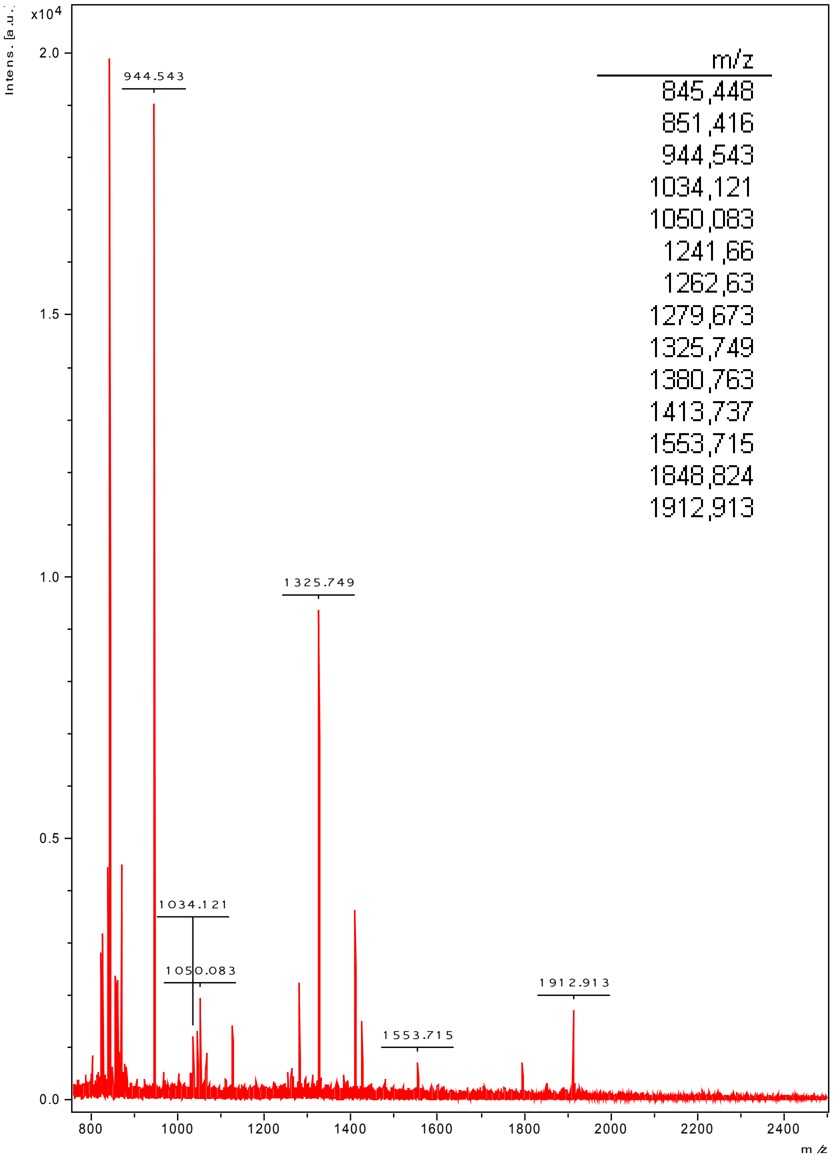

{MATRIX SCIENCE} Mascot Search Results

**User**	: irene
**Email**	: isanh@unileon.es
**Search title**	: spot 6445
**Database**	: NCBInr 20100529 (11111565 sequences; 3786451707 residues)
**Taxonomy**	: Streptomyces coelicolor (8575 sequences)
**Timestamp**	: 3 Jun 2010 at 14:24:41 GMT
**Top Score**	: 84 for gi|21220421, transketolase [Streptomyces coelicolor A3(2)]

Mascot Score Histogram

Protein score is -10*Log(P), where P is the probability that the observed match is a random event.
Protein scores greater than 52 are significant (p<0.05).

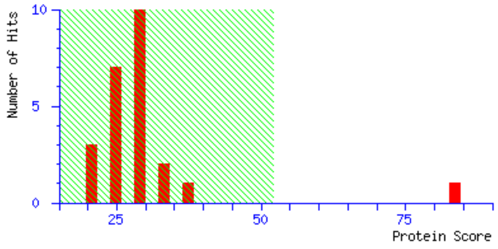

Index

	Accession	Mass	Score	Description
1.	gi|21220421	75085	84	transketolase [Streptomyces coelicolor A3(2)]
2.	gi|7481146	21183	35	probable pantoate-amino acid ligase - Streptomyces coelicolor (fragment)
3.	gi|21219522	43650	35	insertion element [Streptomyces coelicolor A3(2)]
4.	gi|21221636	25020	34	TetR family transcriptional regulator [Streptomyces coelicolor A3(2)]
5.	gi|21221716	26860	32	cobalt-precorrin-6x reductase [Streptomyces coelicolor A3(2)]
6.	gi|21222864	25430	31	MerR family transcriptional regulator [Streptomyces coelicolor A3(2)]
7.	gi|21220839	102585	31	pyruvate dehydrogenase subunit E1 [Streptomyces coelicolor A3(2)]
8.	gi|21225670	34131	30	binding-protein-dependent transport lipoprotein [Streptomyces coelicolor A3(2)]
9.	gi|32141201	35492	29	pantoate--beta-alanine ligase [Streptomyces coelicolor A3(2)]
10.	gi|21218793	33045	28	oxidoreductase [Streptomyces coelicolor A3(2)]

Sequence Coverage: **12%**

Matched peptides shown in **Bold Red**

1 MSTKPTTTDL EWTELDQRAV DTARVLAADA VQKVGNGHPG TAMSLAPAAY
51 TLFQKVMRHD PADANWVGRD RFVLSAGHSS LTLYTQLYLA GFGLELADLE
101 SFRTWGSKTP GHPEYGHTTG VETTTGPLGQ GVANAVGMAM AARYERGLFD
151 PEAAEGESPF DHFVYCIAGD GCLQEGISSE ASSTAGHQKL GNLVLLWDDN
201 HISIEGDTET AVSEDTCKRY EAYGWHVQRV APKPDGDLDP NAIYDAIEAA
251 KKVTDRPSFI AMRSIIAWPA PNAQNTEAAH GSALGDDEVA ATKRVLGFDP
301 EKSFEVSDEV IEHTRK**ALEK GQAAR**AVWEK **SFQQWR**DNNP ERAAEYDR**VA**
351 **KGELPAGWEE K**IPVFEAGKG LATRAASGKV LQALGAVIPE LWGGSADLAG
401 SNNTTIDKTS SFLPAGNPLP EADPYGRTIH FGIR**EHAMAA EMNGIALHGN**
451 **TR**IYGGTFLV FSDYMRNAVR LSALMHLPVT YVWTHDSIGL GEDGPTHQPV
501 EHLASLRAIP GLNVVRPADA NETAIAWREI LRRWTKEFGK **GQPHGLALTR**
551 QGVPTYEPNE DAAKGGYVLF EAEGGDAEVV LIATGSEVHV AVGAR**ERLQA**
601 **DGVPTR**VVSM PSVEWFEQQD QGYRDSVLPP SVKAR**VAVEA GIGLTWHK**YV
651 GDAGRIVSLE HFGASADAKV LFKEFGFTAE NVASAAR**ESL AAAQR**

Peptide Mass Fingerprint for spot 6947: SCO1945

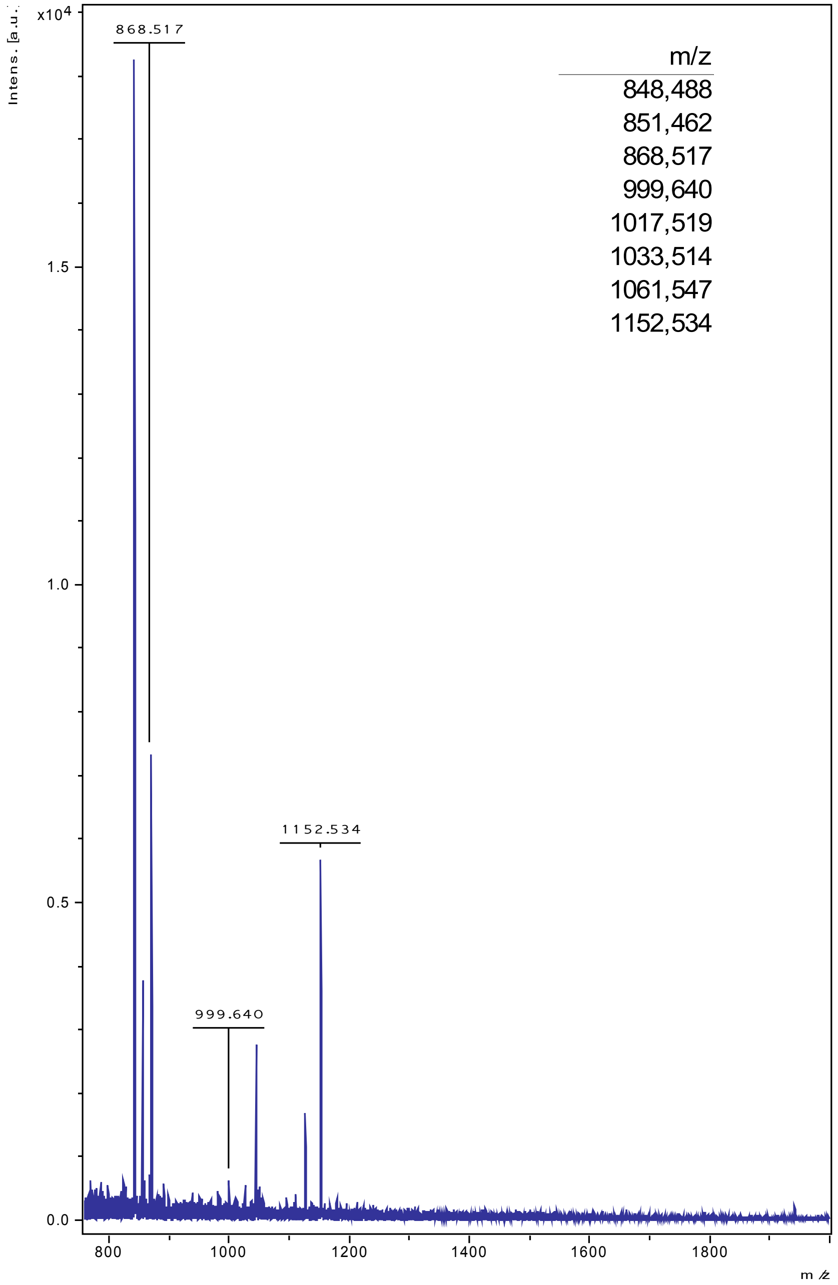

MATRIX SCIENCE Mascot Search Results

**User**	:	**irene**
**Email**	:	**isanh@unileon.es**
**Search title**	:	**spot 6947**
**Database**	:	**NCBInr 20100529 (11111565 sequences; 3786451707 residues)**
**Taxonomy**	:	**Streptomyces coelicolor (8575 sequences)**
**Timestamp**	:	**4 Jun 2010 at 10:58:50 GMT**
**Top Score**	:	**59 for gi|21220430, triosephosphate isomerase [Streptomyces coelicolor A3(2)]**

Mascot Score Histogram

Protein score is -10*Log(P), where P is the probability that the observed match is a random event.
Protein scores greater than 52 are significant (p<0.05).

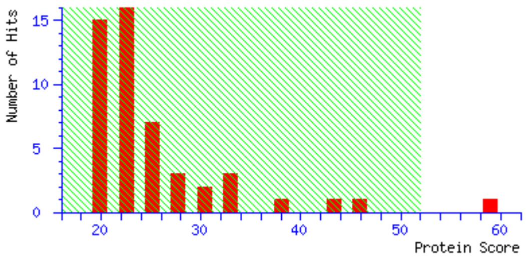

Index

	Accession	Mass	Score	Description
1.	gi|21220430	27947	59	triosephosphate isomerase [Streptomyces coelicolor A3(2)]
2.	gi|21219210	49783	47	ferredoxin/ferredoxin-NADP reductase (putative secreted protein) [Streptomyces coelicolor A3(2)]
3.	gi|21222497	17654	43	lipoprotein [Streptomyces coelicolor A3(2)]
4.	gi|21223671	32237	37	hypothetical protein SCO5308 [Streptomyces coelicolor A3(2)]
5.	gi|21220012	5061	32	hypothetical protein SCO1512 [Streptomyces coelicolor A3(2)]
6.	gi|21219314	37039	32	hypothetical protein SCO0792 [Streptomyces coelicolor A3(2)]
7.	gi|21219714	50275	32	succinic semialdehyde dehydrogenase [Streptomyces coelicolor A3(2)]
8.	gi|21223435	8955	31	hypothetical protein SCO5063 [Streptomyces coelicolor A3(2)]
9.	gi|21222189	48000	30	threonyl tRNA synthetase [Streptomyces coelicolor A3(2)]
10.	gi|21219658	64596	28	ABC transporter ATP-binding protein [Streptomyces coelicolor A3(2)]

Sequence Coverage: **13%**

Matched peptides shown in **Bold Red**

```
  1 MTTRTPLMAG NWKMNLNHLE AIAHVQKLAF ALADKDYDAV EVAVLAPFTD
 51 LRSVQTLVDG DKLKIKYGAQ DISAHDGGAY TGEISGPMLA KLKCTYVAVG
101 HSERRQYHAE TDEIVNAKVK AAYKHGLTPI LCVGEELDVR EAGNHVEHTL
151 AQVEGGLKDL AAEQAESVVI AYEPVWAIGT GKVCGADDAQ EVCAAIRGKL
201 AELYSQELAD KVRIQYGGSV KSGNVAEIMA KPDIDGALVG GASLDSDEFV
251 KIVRFRDQ
```

Peptide Mass Fingerprint for spot 6233: SCO1946

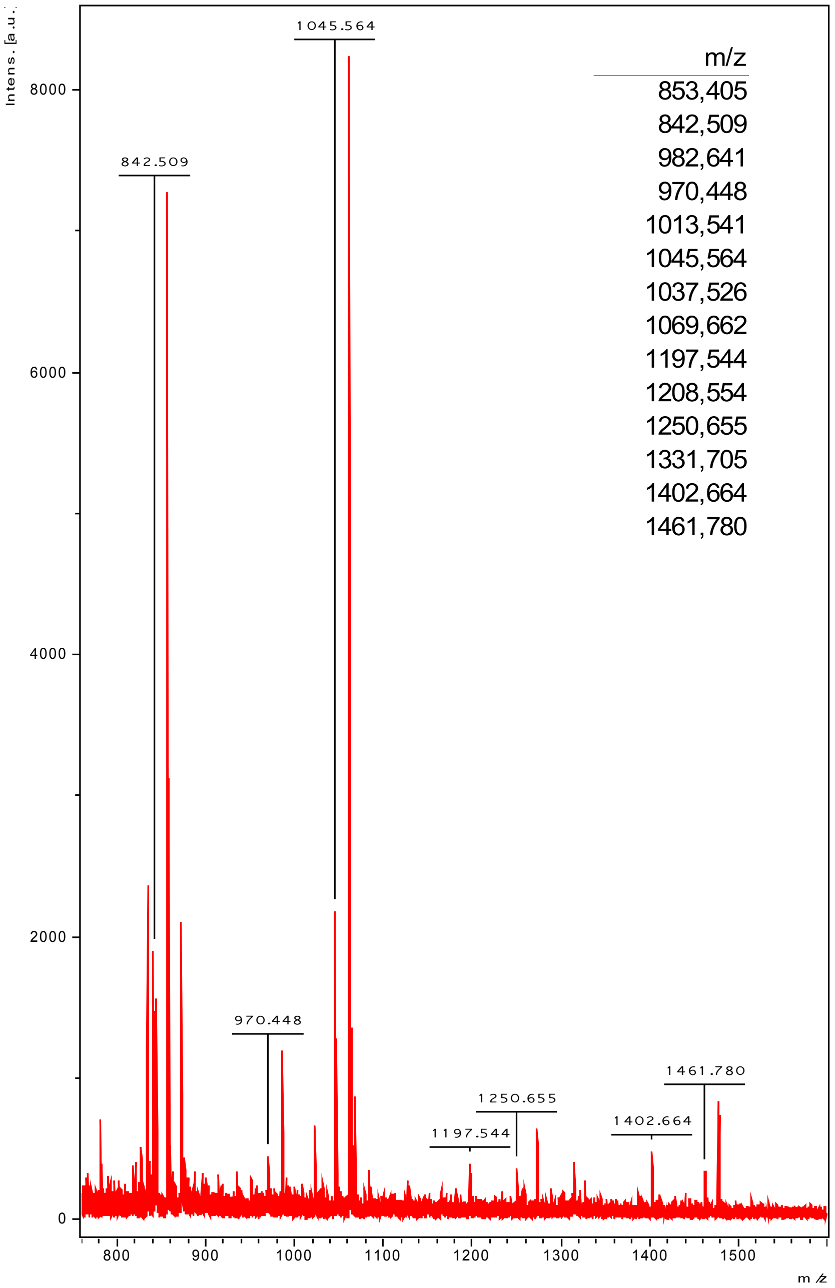

Mascot Search Results

**User**	:	**irene**
**Email**	:	**isanh@unileon.es**
**Search title**	:	**spot 6233**
**Database**	:	**NCBInr 20100529 (11111565 sequences; 3786451707 residues)**
**Taxonomy**	:	**Streptomyces coelicolor (8575 sequences)**
**Timestamp**	:	**4 Jun 2010 at 11:23:26 GMT**
**Top Score**	:	**83 for gi|21220431, phosphoglycerate kinase [Streptomyces coelicolor A3(2)]**

Mascot Score Histogram

Protein score is -10*Log(P), where P is the probability that the observed match is a random event.
Protein scores greater than 52 are significant (p<0.05).

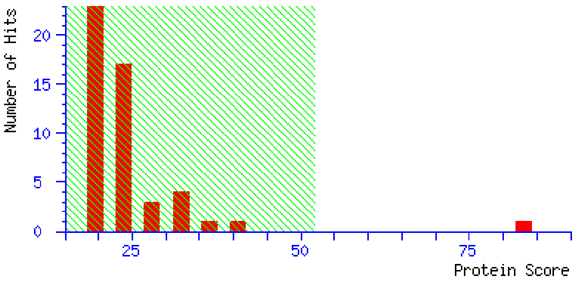

Index

	Accession	Mass	Score	Description
1.	gi|21220431	41740	83	phosphoglycerate kinase [Streptomyces coelicolor A3(2)]
2.	gi|21224012	26447	41	hypothetical protein SCO5661 [Streptomyces coelicolor A3(2)]
3.	gi|21225247	19601	35	hypothetical protein SCO6960 [Streptomyces coelicolor A3(2)]
4.	gi|21222919	47816	34	integral membrane protein [Streptomyces coelicolor A3(2)]
5.	gi|21223436	86651	32	bifunctional protein [Streptomyces coelicolor A3(2)]
6.	gi|21224983	122963	31	hypothetical protein SCO6688 [Streptomyces coelicolor A3(2)]
7.	gi|21225211	26499	30	hypothetical protein SCO6923 [Streptomyces coelicolor A3(2)]
8.	gi|21223951	27759	30	signal peptidase I [Streptomyces coelicolor A3(2)]
9.	gi|21224180	88928	27	DNA topoisomerase IV subunit A [Streptomyces coelicolor A3(2)]
10.	gi|21222693	9560	26	hypothetical protein SCO4300 [Streptomyces coelicolor A3(2)]

Sequence Coverage: **16%**

Matched peptides shown in **Bold Red**

```
  1 MKTIDELLSE GVAGKRVFVR ADLNVPLDGT TITDDGRIRA VVPTVKALAD
 51 AGARVIVASH LGRPKGAPDP AFSLAPAAAR LGELLGADVA FAEDTVGSSA
101 EAVVTGLADG GVAVIENLRF NAGETSKDDA ERAAFADKLA GLADVYVGDG
151 FGAVHRKHAS VFDLPKRLPH YAGYLIATEV GVLKKLTDEV KRPYVVALGG
201 AKVSDKLAVI DQLLGKADRL LIGGGMAYTF LKAKGYEVGI SLLQEDQIPA
251 VKEYIERAEK NGVELVLPVD VLVAPEFPDL KTKAPANPTT VAADAIPADQ
301 EGLDIGPETR KLYASKLADA GTVFWNGPMG VFEHPDYAEG TKAVAQALVD
351 APGFTVVGGG DSAAAVRTLG FDENAFGHIS TGGGASLEYL EGKTLPGLAA
401 LED
```

Matched (bold red) peptides: TIDELLSE GVAGK (1–15); IRA VVPTVK (38–46); F NAGETSKDDA ERAAFADK (120–138); HAS VFDLPK (158–166); LAVI DQLLGK (207–216); EYIERAEK (253–260).

Peptide Mass Fingerprint for spot 6193: SCO1947

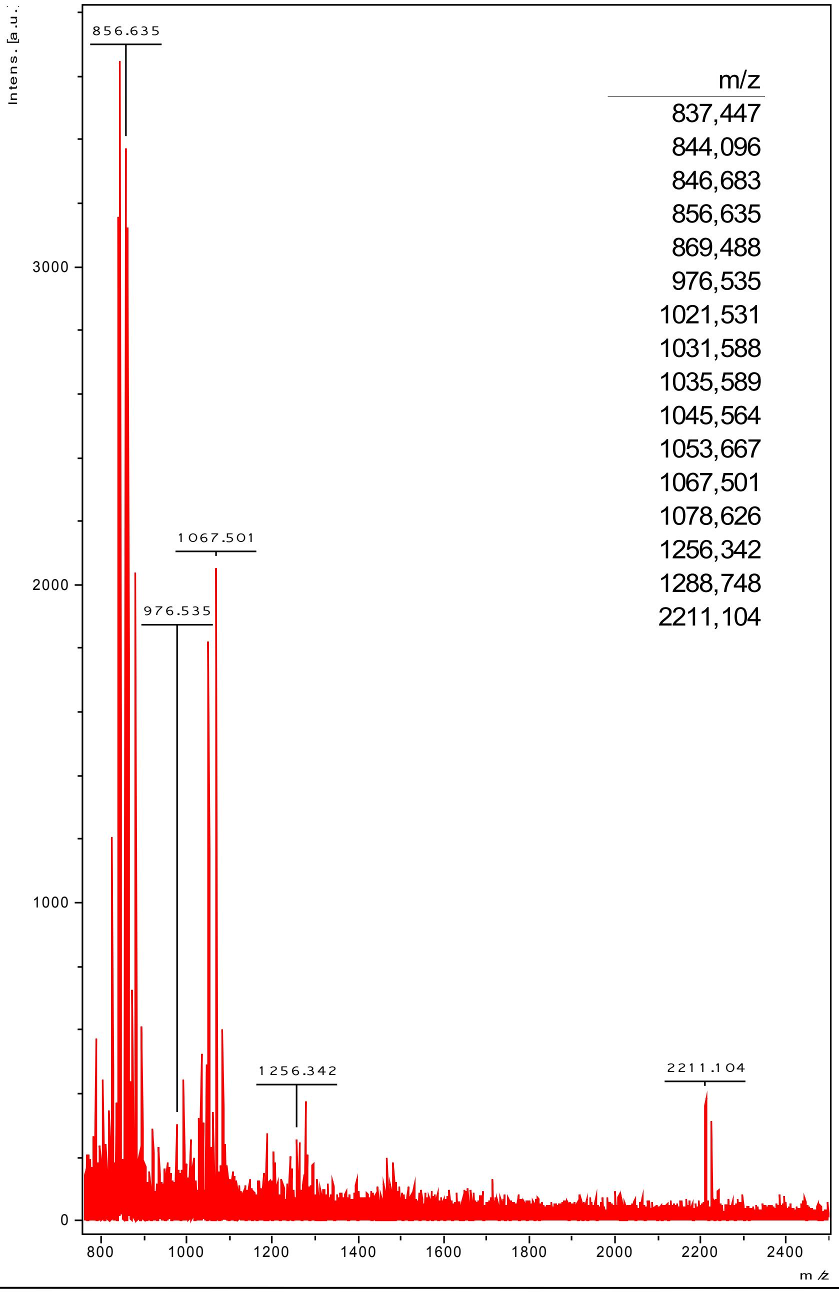

Mascot Search Results

```
User            : irene
Email           : isanh@unileon.es
Search title    : spot 6193
Database        : NCBInr 20100529 (11111565 sequences; 3786451707 residues)
Taxonomy        : Streptomyces coelicolor (8575 sequences)
Timestamp       : 4 Jun 2010 at 11:38:51 GMT
Top Score       : 65 for gi|21220432, glyceraldehyde-3-phosphate dehydrogenase [Streptomyces coelicolor A3(2)]
```

Mascot Score Histogram

Protein score is -10*Log(P), where P is the probability that the observed match is a random event.
Protein scores greater than 52 are significant (p<0.05).

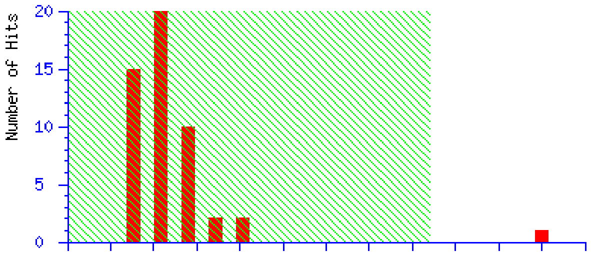

Index

	Accession	Mass	Score	Description
1.	gi|21220432	36417	65	glyceraldehyde-3-phosphate dehydrogenase [Streptomyces coelicolor A3(2)]
2.	gi|21218661	25682	30	hypothetical protein SCO0095 [Streptomyces coelicolor A3(2)]
3.	gi|21223676	31813	29	membrane protein [Streptomyces coelicolor A3(2)]
4.	gi|21225026	77958	28	fatty acid oxidative multifunctional enzyme [Streptomyces coelicolor A3(2)]
5.	gi|32141232	31838	26	aminoglycoside phosphotransferase [Streptomyces coelicolor A3(2)]
6.	gi|21220047	9476	25	hypothetical protein SCO1548 [Streptomyces coelicolor A3(2)]
7.	gi|21219602	39255	25	aldolase [Streptomyces coelicolor A3(2)]
8.	gi|21222458	40462	25	alcohol dehydrogenase [Streptomyces coelicolor A3(2)]
9.	gi|21224018	42554	24	polyamine ABC transporter ATP-binding protein [Streptomyces coelicolor A3(2)]
10.	gi|21223774	11954	24	hypothetical protein SCO5414 [Streptomyces coelicolor A3(2)]

Sequence Coverage: 13%

Matched peptides shown in **Bold Red**

1 MTIRVGINGF GRIGRNYFRA LLEQGADIEI VAVNDLGDTA TTAHLLK**YDT**
51 **ILGRLK**AEVS HTEDTITVDG KTIKVLSERN PADIPWGELG VDIVIESTGI
101 FTKKADAEKH IAGGAKKVLI SAPAKDEDIT IVMGVNQDKY DPANHHVISN
151 ASCTTNCVAP MAKVLDENFG IVKGLMTTVH AYTNDQR**ILD** **FPHK**DLRRAR
201 AAAENIIPTT TGAAK**ATALV** **LPQLKGKLDG** **IAMR**VPVPTG SATDLVVELQ
251 REVTK**DEVNA** **AFKK**AADDGD LKGILFYTED AIVSSDITGD PASCTFDSSL
301 TMVQEGKSVK ILGWYDNEWG YSNRLVDLTV FVGNQL

Peptide Mass Fingerprint for spot 6666: SCO1965

m/z
893,495
978,485
1084,538
1086,551
1139,597
1734,719

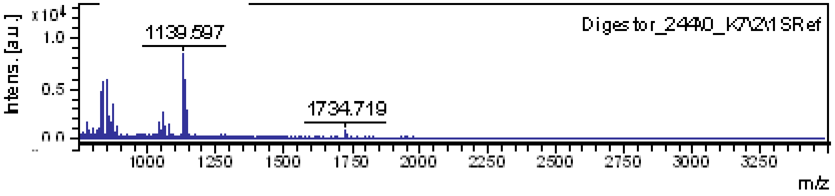

Mascot Search Results

```
User            : irene
Email           : isanh@unileon.es
Search title    : spot 6666
Database        : NCBInr 20100602 (11126451 sequences; 3793134231 residues)
Taxonomy        : Streptomyces coelicolor (8575 sequences)
Timestamp       : 8 Jun 2010 at 10:54:38 GMT
Top Score       : 68 for gi|21220450, export associated protein [Streptomyces coelicolor A3(2)]
```

Mascot Score Histogram

Protein score is -10*Log(P), where P is the probability that the observed match is a random event.
Protein scores greater than 52 are significant (p<0.05).

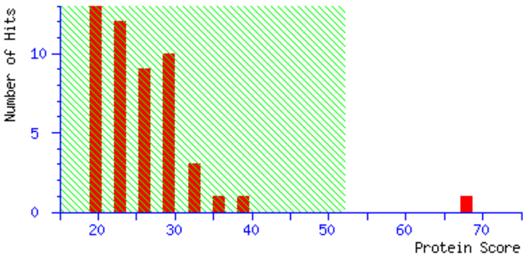

Index

	Accession	Mass	Score	Description
1.	gi|21220450	20619	68	export associated protein [Streptomyces coelicolor A3(2)]
2.	gi|21225517	81595	40	serine/threonine-protein kinase [Streptomyces coelicolor A3(2)]
3.	gi|21220979	47829	36	two-component sensor kinase [Streptomyces coelicolor A3(2)]
4.	gi|3451475	60022	33	hypothetical protein [Streptomyces coelicolor]
5.	gi|21223088	30312	33	30S ribosomal protein S3 [Streptomyces coelicolor A3(2)]
6.	gi|21220759	12371	32	hypothetical protein SCO2290 [Streptomyces coelicolor A3(2)]
7.	gi|21219250	14151	30	hypothetical protein SCO0725 [Streptomyces coelicolor A3(2)]
8.	gi|21219394	10060	30	hypothetical protein SCO0873 [Streptomyces coelicolor A3(2)]
9.	gi|21222740	62844	30	hypothetical protein SCO4349 [Streptomyces coelicolor A3(2)]
10.	gi|21222241	14933	30	transcriptional regulator [Streptomyces coelicolor A3(2)]

Sequence Coverage: 19%

Matched peptides shown in **Bold Red**

```
  1 MTVNMTKGQA ISLQKSDGGS LTSVRMGLGW QAAPRRGLFG SRTREIDLDA
 51 SAVLFADKQP VDVVFFRHLV SDDGSVRHTG DNLVGGVGQG GDDEAILVDL
101 QRVPVHIDQI VFTVNSFTGQ TFQEVQNAFC RLVDETNGQE LARYTLAGGG
151 AFTAQIMAKV HRAGQGWSMT AIGTPANGRT FQDLMPAILP VL
```

Peptide Mass Fingerprint for spot 6494: SCO1998

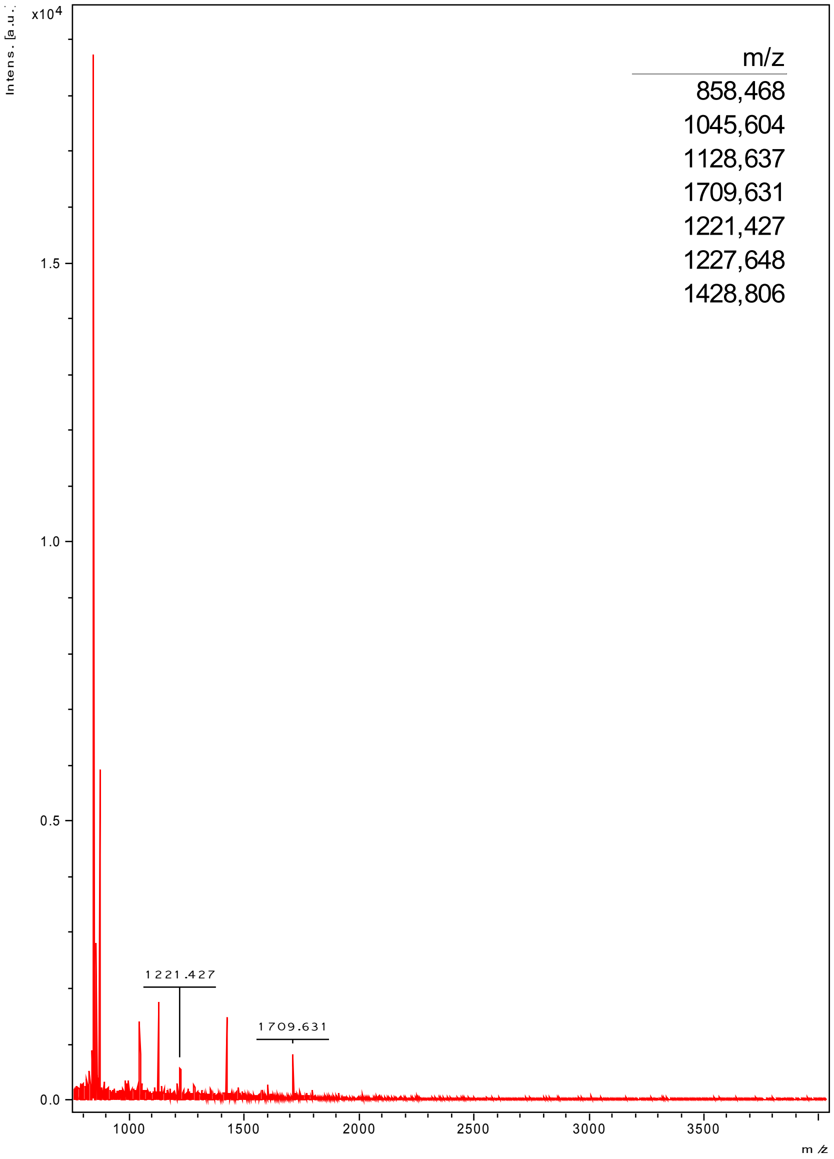

Mascot Search Results

User : irene
Email : isanh@unileon.es
Search title : spot 6494
Database : NCBInr 20100602 (11126451 sequences; 3793134231 residues)
Taxonomy : Streptomyces coelicolor (8575 sequences)
Timestamp : 8 Jun 2010 at 07:51:53 GMT
Top Score : 66 for gi|21220480, 30S ribosomal protein S1 [Streptomyces coelicolor A3(2)]

Mascot Score Histogram

Protein score is -10*Log(P), where P is the probability that the observed match is a random event.
Protein scores greater than 52 are significant (p<0.05).

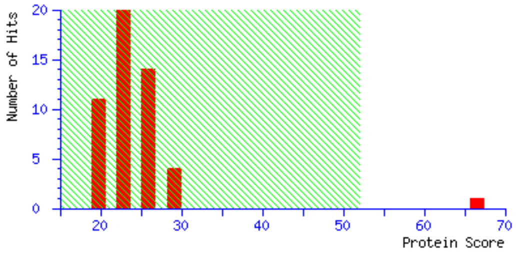

Index

	Accession	Mass	Score	Description
1.	gi|21220480	55001	66	30S ribosomal protein S1 [Streptomyces coelicolor A3(2)]
2.	gi|21224283	64042	30	transferase [Streptomyces coelicolor A3(2)]
3.	gi|21225057	75453	29	squalene-hopene cyclase [Streptomyces coelicolor A3(2)]
4.	gi|21219928	18002	28	transcription regulator AsnC [Streptomyces coelicolor A3(2)]
5.	gi|21222766	16859	28	hypothetical protein SCO4376 [Streptomyces coelicolor A3(2)]
6.	gi|21222268	19302	27	DNA-binding protein [Streptomyces coelicolor A3(2)]
7.	gi|21223657	21058	27	ATP/GTP-binding protein [Streptomyces coelicolor A3(2)]
8.	gi|21221965	20525	27	small secreted protein [Streptomyces coelicolor A3(2)]
9.	gi|21219079	55209	27	hypothetical protein SCO0544 [Streptomyces coelicolor A3(2)]
10.	gi|21224780	21162	26	adenylosuccinate lyase [Streptomyces coelicolor A3(2)]

Sequence Coverage: 8%

Matched peptides shown in **Bold Red**

```
  1 MTSSTETTAT TPQVAVNDIG NEEAFLAAID ETIKYFNDGD IVDGVIVKVD
 51 RDEVLLDIGY KTEGVIPSRE LSIKHDVDPN EVVAVGDEIE ALVLQKEDKE
101 GRLILSKKRA QYERAWGTIE KIKEEDGIVT GTVIEVVKGG LILDIGLRGF
151 LPASLVEMRR VRDLQPYVGK ELEAKIIELD KNRNNVVLSR RAWLEQTQSE
201 VRQTFLTTLQ KGQVRSGVVS SIVNFGAFVD LGGVDGLVHV SELSWKHIDH
251 PSEVVEVGQE VTVEVLDVDM DRERVSLSLK ATQEDPWQQF ARTHQIGQVV
301 PGKVTKLVPF GAFVRVDEGI EGLVHISELA ERHVEIPEQV VQVNDEIFVK
351 VIDIDLERRR ISLSLKQANE AFGADPSTVE FDPTLYGMAA SYDDQGNYIY
401 PEGFDPETND WLEGYETQRE AWETQYAEAQ TRFEQHQAQV IKSREADEKA
451 AAEGVDTAGA APAASGGGGG GSYSSEGGDN SGALASDEAL AALREKLAGG
501 QS
```

Matched peptides (bold red in original): TEGVIPSRE LSIK (61–74), AWGTIE KIK (115–123), VIDIDLERR (351–359), FEQHQAQV IK (433–442)

Peptide Mass Fingerprint for spot 6143: SCO2198

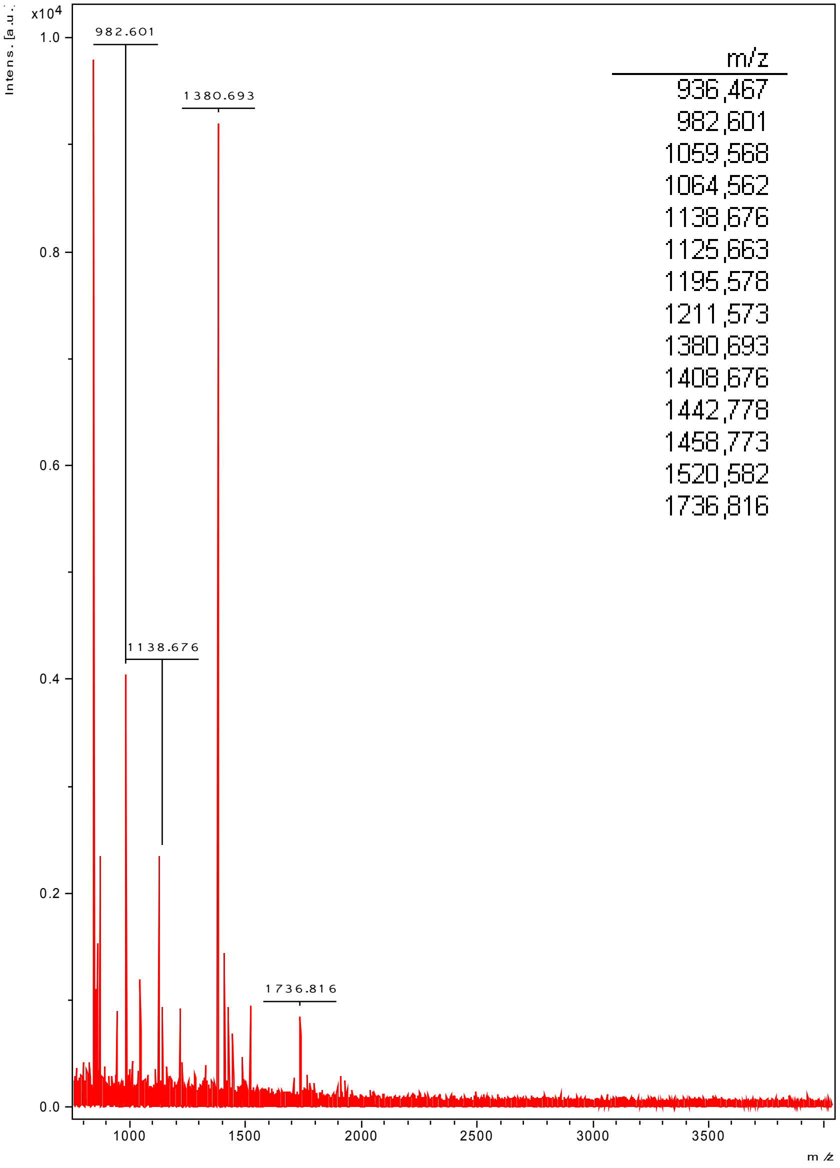

Mascot Search Results

User : irene
Email : isanh@unileon.es
Search title : spot 6143
Database : NCBInr 20100529 (11111565 sequences; 3786451707 residues)
Taxonomy : Streptomyces coelicolor (8575 sequences)
Timestamp : 3 Jun 2010 at 13:41:41 GMT
Top Score : 68 for gi|21220671, glutamine synthetase I [Streptomyces coelicolor A3(2)]

Mascot Score Histogram

Protein score is -10*Log(P), where P is the probability that the observed match is a random event.
Protein scores greater than 52 are significant (p<0.05).

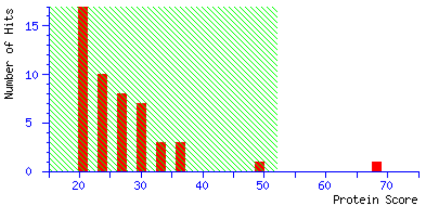

Index

	Accession	Mass	Score	Description
1.	gi|21220671	52592	68	glutamine synthetase I [Streptomyces coelicolor A3(2)]
2.	gi|21225936	7597	48	ferredoxin [Streptomyces coelicolor A3(2)]
3.	gi|21221444	17348	38	hypothetical protein SCO3001 [Streptomyces coelicolor A3(2)]
4.	gi|21223700	49278	37	plasmid transfer protein [Streptomyces coelicolor A3(2)]
5.	gi|21234112	42059	35	ecf family sigma factor [Streptomyces coelicolor A3(2)]
6.	gi|21223661	24234	33	TetR family transcriptional regulator [Streptomyces coelicolor A3(2)]
7.	gi|21225342	24951	32	oxidoreductase [Streptomyces coelicolor A3(2)]
8.	gi|21223654	27622	32	hypothetical protein SCO5288 [Streptomyces coelicolor A3(2)]
9.	gi|21220865	16638	32	MarR family transcriptonal regulator [Streptomyces coelicolor A3(2)]
10.	gi|21224042	71920	31	acyl CoA dehydrogenase [Streptomyces coelicolor A3(2)]

Sequence Coverage: **18%**

Matched peptides shown in **Bold Red**

```
  1 MFQNADDVKK FIADEDVKFV DVRFCDLPGV MQHFTLPATA FDPDAEQAFD
 51 GSSIRGFQAI HESDMSLRPD LSTARVDPFR RDKTLNINFF IHDPITGEQY
101 SRDPRNVAKK AEAYLASTGI ADTAFFGPEA EFYVFDSVRF ATRENESFYH
151 IDSEAGAWNT GALEDNRGYK VRYKGGYFPV PPVDHFADLR AEISLELERS
201 GLQVERQHHE VGTAGQAEIN YKFNTLLAAA DDLQLFKYIV KNVAWKNGKT
251 ATFMPKPIFG DNGSGMHVHQ SLWSGGEPLF YDEQGYAGLS DTARYYIGGI
301 LKHAPSLLAF TNPTVNSYHR LVPGFEAPVN LVYSQRNRSA AMRIPITGSN
351 PKAKRVEFRA PDASGNPYLA FSALLLAGLD GIKNKIEPAE PIDKDLYELA
401 PEEHANVAQV PTSLGAVLDR LEADHEFLLQ GDVFTPDLIE TWIDFKRANE
451 IAPLQLRPHP HEFEMYFDV
```

Matched (bold red) peptides: MFQNADDVKK FIADEDVKFV DVR (1–23); GGYFPV PPVDHFADLR AEISLELERS (175–200); YIV KNVAWK (238–246); NRSA AMRIPITGSN PKAK (337–354); NKIEPAE PIDK (384–394).

Peptide Mass Fingerprint for spot 6647: SCO2368

m/z
975,020
949,485
1030,522
1066,049
1092,580
1255,044
1256,659
1445,832
2210,902

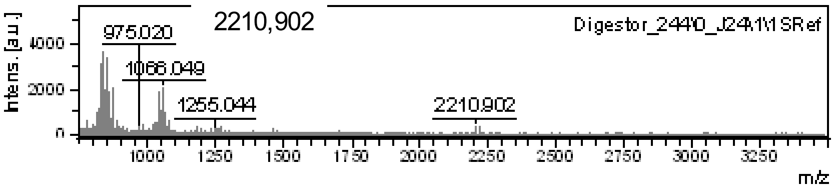

Mascot Search Results

```
User          : irene
Email         : isanh@unileon.es
Search title  : spo7 6647
Database      : NCBInr 20100602 (11126451 sequences; 3793134231 residues)
Taxonomy      : Streptomyces coelicolor (8575 sequences)
Timestamp     : 8 Jun 2010 at 11:44:28 GMT
Top Score     : 59 for gi|21220836, hypothetical protein SCO2368 [Streptomyces coelicolor A3(2)]
```

Mascot Score Histogram

Protein score is -10*Log(P), where P is the probability that the observed match is a random event.
Protein scores greater than 52 are significant (p<0.05).

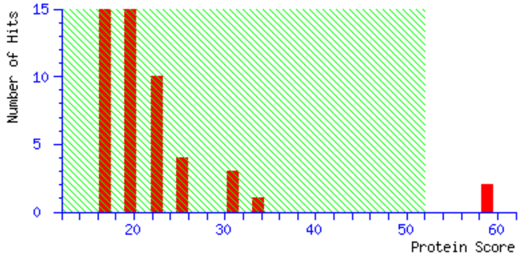

Index

	Accession	Mass	Score	Description
1.	gi|21220836	20375	59	hypothetical protein SCO2368 [Streptomyces coelicolor A3(2)]
2.	gi|256032870	20335	59	Chain A, Crystal Structure Of Putative Tellurium Resistant Like Protein (Terd) From Strepton
3.	gi|21219079	55209	34	hypothetical protein SCO0544 [Streptomyces coelicolor A3(2)]
4.	gi|21222562	9736	32	hypothetical protein SCO4165 [Streptomyces coelicolor A3(2)]
5.	gi|21225808	44087	31	CRP family transcriptional regulator [Streptomyces coelicolor A3(2)]
6.	gi|21225929	36194	30	oxidoreductase [Streptomyces coelicolor A3(2)]
7.	gi|18077876	16303	26	hypothetical protein 2_SCP2_p01 [Streptomyces coelicolor]
8.	gi|21222099	18803	25	hypothetical protein SCO3686 [Streptomyces coelicolor A3(2)]
9.	gi|21223227	17639	25	hypothetical protein SCO4851 [Streptomyces coelicolor A3(2)]
10.	gi|21233998	17404	25	putative regulatory protein [Streptomyces coelicolor A3(2)]

Sequence Coverage: 20%

Matched peptides shown in **Bold Red**

1 MGVSLSKGGN VSLTKEAPGL TAVIVGLGWD IRTTTGTDFD LDASALLLNS
51 GGKVASDAHF IFFNNLKSPD GSVEHTGDNI TGEGEGDDEQ IKINLATVPA
101 DIEKIVFPVS IYDAENR**QQS FGQVR**NAFIR **VVNQAGEAEI AR**YDLSEDAS
151 TETAMVFGEL YR**HGAEWKFR AIGQGYASGL R**GIAQDFGVN V

Peptide Mass Fingerprint for spot 6685: SCO2633

m/z
842,498
1050,082
1085,631
2211,104
2657,321
2691,139
3316,596
3332,591
3337,347

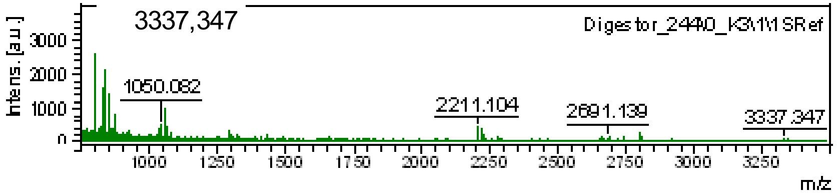

Mascot Search Results

```
User          : irene
Email         : isanh@unileon.es
Search title  : spot 6685
Database      : NCBInr 20100602 (11126451 sequences; 3793134231 residues)
Taxonomy      : Streptomyces coelicolor (8575 sequences)
Timestamp     : 8 Jun 2010 at 10:18:46 GMT
Top Score     : 66 for gi|21221090, superoxide dismutase [Fe-Zn] [Streptomyces coelicolor A3(2)]
```

Mascot Score Histogram

Protein score is -10*Log(P), where P is the probability that the observed match is a random event.
Protein scores greater than 52 are significant (p<0.05).

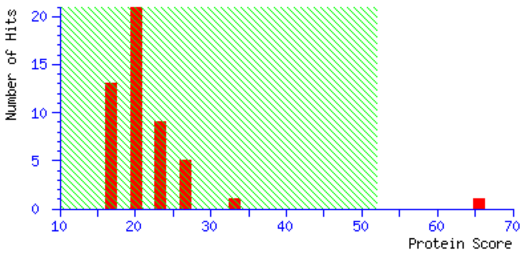

Index

	Accession	Mass	Score	Description
1.	gi|21221090	23513	66	superoxide dismutase [Fe-Zn] [Streptomyces coelicolor A3(2)]
2.	gi|286143258	17854	33	Fe-containing superoxide dismutase [Streptomyces coelicolor]
3.	gi|21221379	30298	28	permease membrane component [Streptomyces coelicolor A3(2)]
4.	gi|21223226	28257	26	TetR family transcriptional regulator [Streptomyces coelicolor A3(2)]
5.	gi|167013500	30656	26	Chain A, Crystal Structure Of A Tetr Family Transcriptional Regulator From Streptomyces Coeli
6.	gi|21221880	29237	25	ABC-transporter transport protein [Streptomyces coelicolor A3(2)]
7.	gi|32141144	45263	25	bifunctional phosphopantothenoylcysteine decarboxylase/phosphopantothenate synthase [Streptom
8.	gi|21223650	24914	25	hypothetical protein SCO5284 [Streptomyces coelicolor A3(2)]
9.	gi|21222422	25807	25	gntR family regulatory protein [Streptomyces coelicolor A3(2)]
10.	gi|21220913	37178	24	hypothetical protein SCO2449 [Streptomyces coelicolor A3(2)]

Sequence Coverage: 30%

Matched peptides shown in **Bold Red**

```
  1 MSVYTLPELP YDYSALAPVI SPEIIELHHD KHHAAYVKGA NDTLEQLAEA
 51 RDKETWGSIN GLEKNLAFHL SGHILHSIYW HNMTGDGGGE PLDKDGVGEL
101 ADAIAESFGS FAGFRAQLTK AAATTQGSGW GVLAYEPLSG RLIVEQIYDH
151 QGNVGQGSTP ILVFDAWEHA FYLQYKNQKV DFIDAMWAVV NWQDVARRYE
201 AAKSRTNTLL LAP
```

Peptide Mass Fingerprint for spot 6366: SCO3649

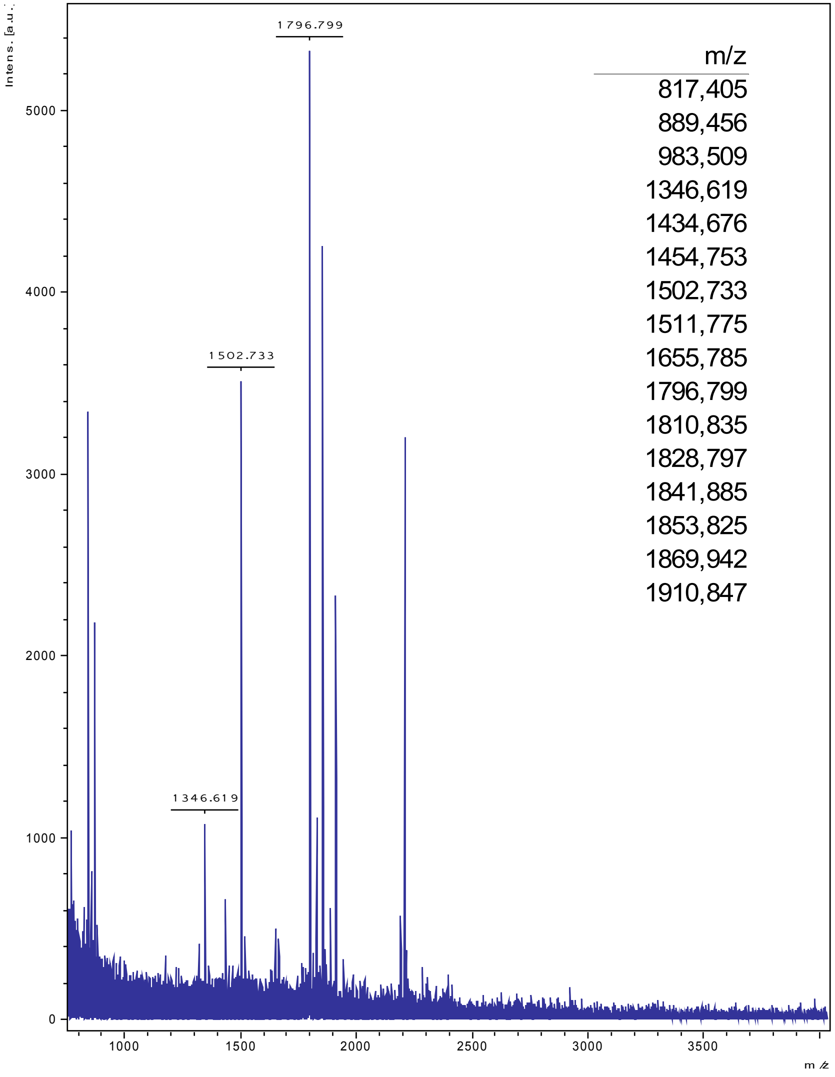

m/z
817,405
889,456
983,509
1346,619
1434,676
1454,753
1502,733
1511,775
1655,785
1796,799
1810,835
1828,797
1841,885
1853,825
1869,942
1910,847

MATRIX SCIENCE Mascot Search Results

**User**	:	irene
**Email**	:	isanh@unileon.es
**Search title**	:	spot 6366
**Database**	:	NCBInr 20100602 (11126451 sequences; 3793134231 residues)
**Taxonomy**	:	Streptomyces coelicolor (8575 sequences)
**Timestamp**	:	7 Jun 2010 at 11:25:35 GMT
**Top Score**	:	69 for gi|21222064, fructose-bisphosphate aldolase [Streptomyces coelicolor A3(2)]

Mascot Score Histogram

Protein score is -10*Log(P), where P is the probability that the observed match is a random event.
Protein scores greater than 52 are significant (p<0.05).

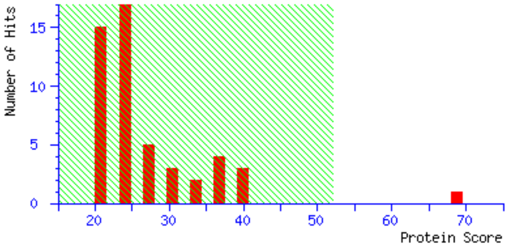

Index

	Accession	Mass	Score	Description
1.	gi|21222064	37018	69	fructose-bisphosphate aldolase [Streptomyces coelicolor A3(2)]
2.	gi|21219352	34299	41	hypothetical protein SCO0831 [Streptomyces coelicolor A3(2)]
3.	gi|21221068	35706	40	rod shape-determining protein MreC [Streptomyces coelicolor A3(2)]
4.	gi|2253080	38571	39	mreC [Streptomyces coelicolor A3(2)]
5.	gi|4927470	39068	38	transposase [Streptomyces coelicolor A3(2)]
6.	gi|21223078	39170	38	IS1652 transposase [Streptomyces coelicolor A3(2)]
7.	gi|21219973	39144	38	transposase [Streptomyces coelicolor A3(2)]
8.	gi|21221089	39158	38	transposase [Streptomyces coelicolor A3(2)]
9.	gi|21223731	57449	33	FOF1 ATP synthase subunit alpha [Streptomyces coelicolor A3(2)]
10.	gi|21222741	50035	33	integrase [Streptomyces coelicolor A3(2)]

Sequence Coverage: **17%**

Matched peptides shown in **Bold Red**

1 **MPIATPEVYN EMLDR**AKAGK FAYPAINVTS SQTLHAALRG FAEAESDGIV

51 QISTGGAEFL GGQHNKDMVT GAVALAEFAH IVAEK**YDVTV ALHTDHCPK**D

101 KLDGYVRPLI AVSEERVKAG RNPLFQSHMW DGSAETLADN LSIAQELLAR

151 ARAARIILEV EITPTGGEED GVSHEINDSL YTTVDDAVRT VEALGLGEKG

201 RYLLAASFGN VHGVYKPGNV VLRPELLKEL NEGIASKYGQ PAGSKPFDFV

251 FHGGSGSTAE EIATALENGV VKMNIDTDTQ YAFTRPVVDH MFRNYDGVLK

301 **VDGEVGNK**KT YDPRTWGK**LA EAGMAARVVE ACGHLRSAGQ K**IK

Peptide Mass Fingerprint for spot 6264: SCO4770

m/z
885,552
922,522
1007,582
1100,595
1403,764
1424,801
1682,894
1791,766

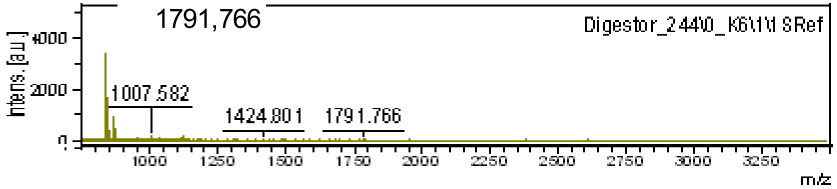

Mascot Search Results

```
User          : irene
Email         : isanh@unileon.es
Search title  : spot 6264
Database      : NCBInr 20100602 (11126451 sequences; 3793134231 residues)
Taxonomy      : Streptomyces coelicolor (8575 sequences)
Timestamp     : 9 Jun 2010 at 09:12:57 GMT
Top Score     : 62 for gi|21223149, inosine 5' monophosphate dehydrogenase [Streptomyces coelicolor A3(2)]
```

Mascot Score Histogram

Protein score is -10*Log(P), where P is the probability that the observed match is a random event.
Protein scores greater than 52 are significant (p<0.05).

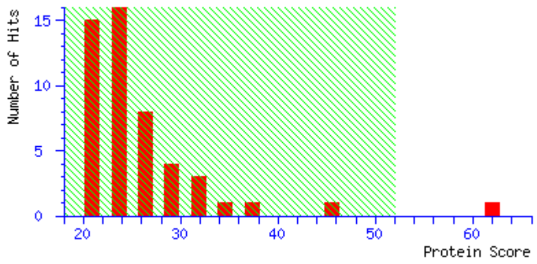

Index

	Accession	Mass	Score	Description
1.	gi|21223149	52614	62	inosine 5' monophosphate dehydrogenase [Streptomyces coelicolor A3(2)]
2.	gi|21224354	59624	46	hypothetical protein SCO6022 [Streptomyces coelicolor A3(2)]
3.	gi|21223850	80865	37	NAD-dependent DNA ligase LigA [Streptomyces coelicolor A3(2)]
4.	gi|21220490	43534	34	branched chain amino acid binding protein [Streptomyces coelicolor A3(2)]
5.	gi|21226033	34109	33	transcriptional regulator [Streptomyces coelicolor A3(2)]
6.	gi|21223063	49622	32	glutamate dehydrogenase [Streptomyces coelicolor A3(2)]
7.	gi|21223616	52389	31	nucleotide-binding protein [Streptomyces coelicolor A3(2)]
8.	gi|21222098	14747	29	hypothetical protein SCO3685 [Streptomyces coelicolor A3(2)]
9.	gi|21234249	15421	28	hypothetical protein SCP1.250 [Streptomyces coelicolor A3(2)]
10.	gi|21223805	71361	28	neutral zinc metalloprotease [Streptomyces coelicolor A3(2)]

Sequence Coverage: 7%

Matched peptides shown in **Bold Red**

```
  1 MTANVDGVPE KFATLGLTYD DVLLLPGASA VLPNAVDTSS RISRNVRVNI
 51 PLLSAAMDKV TESRMAISMA RQGGVGVLHR NLSIEDQANQ VDLVKRSESG
101 MVANPITIHP DATLGEADAL CAKFRISGVP VTDGAGKLLG IVTNRDMAFE
151 TDRSRQVREV MTPMPLVTGQ VGISGVDAME LLRRHKIEKL PLVDGDGILK
201 GLITVKDFVK AEQYPHAAKD AKGRLLVGAA VGASPEALDR AQALAEAGVD
251 FLVVDTSHGH NSNALSWMSK IKSSVGIDVV GGNVATRDGA QALIDAGVDG
301 IKVGVGPGSI CTTRVVAGIG VPQVTAIYEA SLAARAAGVP LIGDGGLQYS
351 GDIGKALAAG ADTVMLGSLL AGCEESPGEL QFINGKQFKS YRGMGSLGAM
401 QSRGQGRSYS KDRYFQAEVA SDDKLVPEGI EGQVPYRGPL ANVLHQLVGG
451 LRQTMGYVGA ATIEEMESKG RFVRITSAGL KESHPHDIQM TVEAPNYSRS
501 K
```

Peptide Mass Fingerprint for spot 6269: SCO4771

m/z
916,510
1029,394
1045,564
1101,626
1301,758
1402,664
1450,732
1597,754
1699,887

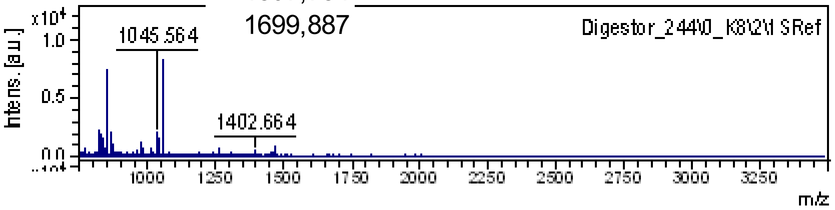

Mascot Search Results

User : irene
Email : isanh@unileon.es
Search title : spot 6269
Database : NCBInr 20100602 (11126451 sequences; 3793134231 residues)
Taxonomy : Streptomyces coelicolor (8575 sequences)
Timestamp : 9 Jun 2010 at 10:40:44 GMT
Top Score : 67 for gi|21223150, inosine 5-monophosphate dehydrogenase [Streptomyces coelicolor A3(2)]

Mascot Score Histogram

Protein score is -10*Log(P), where P is the probability that the observed match is a random event.
Protein scores greater than 52 are significant (p<0.05).

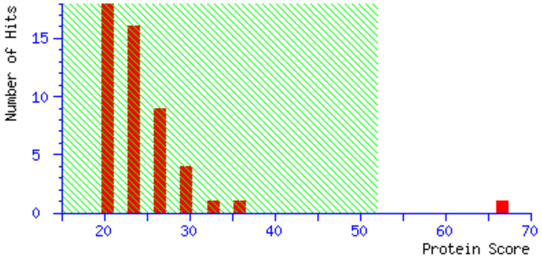

Index

	Accession	Mass	Score	Description
1.	gi|21223150	40205	67	inosine 5-monophosphate dehydrogenase [Streptomyces coelicolor A3(2)]
2.	gi|21223094	20840	36	50S ribosomal protein L5 [Streptomyces coelicolor A3(2)]
3.	gi|21223258	35743	33	lipoprotein [Streptomyces coelicolor A3(2)]
4.	gi|21223436	86651	31	bifunctional protein [Streptomyces coelicolor A3(2)]
5.	gi|21223194	49256	30	serine/threonine protein kinase [Streptomyces coelicolor A3(2)]
6.	gi|21224476	51284	30	ADA-like regulatory protein [Streptomyces coelicolor A3(2)]
7.	gi|21218689	13519	29	hypothetical protein SCO0129 [Streptomyces coelicolor A3(2)]
8.	gi|21225083	66344	27	long chain fatty acid CoA ligase [Streptomyces coelicolor A3(2)]
9.	gi|21222649	101193	27	hypothetical protein SCO4254 [Streptomyces coelicolor A3(2)]
10.	gi|21221892	56747	27	transposase [Streptomyces coelicolor A3(2)]

Sequence Coverage: 17%

Matched peptides shown in **Bold Red**

```
  1 MTEIEIGRGK RGRRAYAFDD IAVVPSRRTR DPKEVSIAWQ IDAYRFELPF
 51 LAAPMDSVVS PATAIRIGEL GGLGVLNLEG LWTRHEDPQP LLDEIAELDT
101 DNATRRLQEI YAAPIKEELI GQRIKEVRDS GVVTAAALSP QRTAQFSKAV
151 VDAGVDIFVI RGTTVSAEHV SGSHEPLNLK QFIYELDVPV IVGGCATYTA
201 ALHLMRTGAA GVLVGFGGGA AHTTRNVLGI QVPMATAVAD VAAARRDYMD
251 ESGGRYVHVI ADGGVGWSGD LPKAIACGAD SVMMGSPLAR ATDAPGRGNH
301 WGMEAVNEEL PRGKKVDLGT VGTIEEILTG PSRNPDGSMN FFGALRRAMA
351 TTGYSELKEF QRVEVTVADS QHRR
```

Peptide Mass Fingerprint for spot 6400: SCO4809

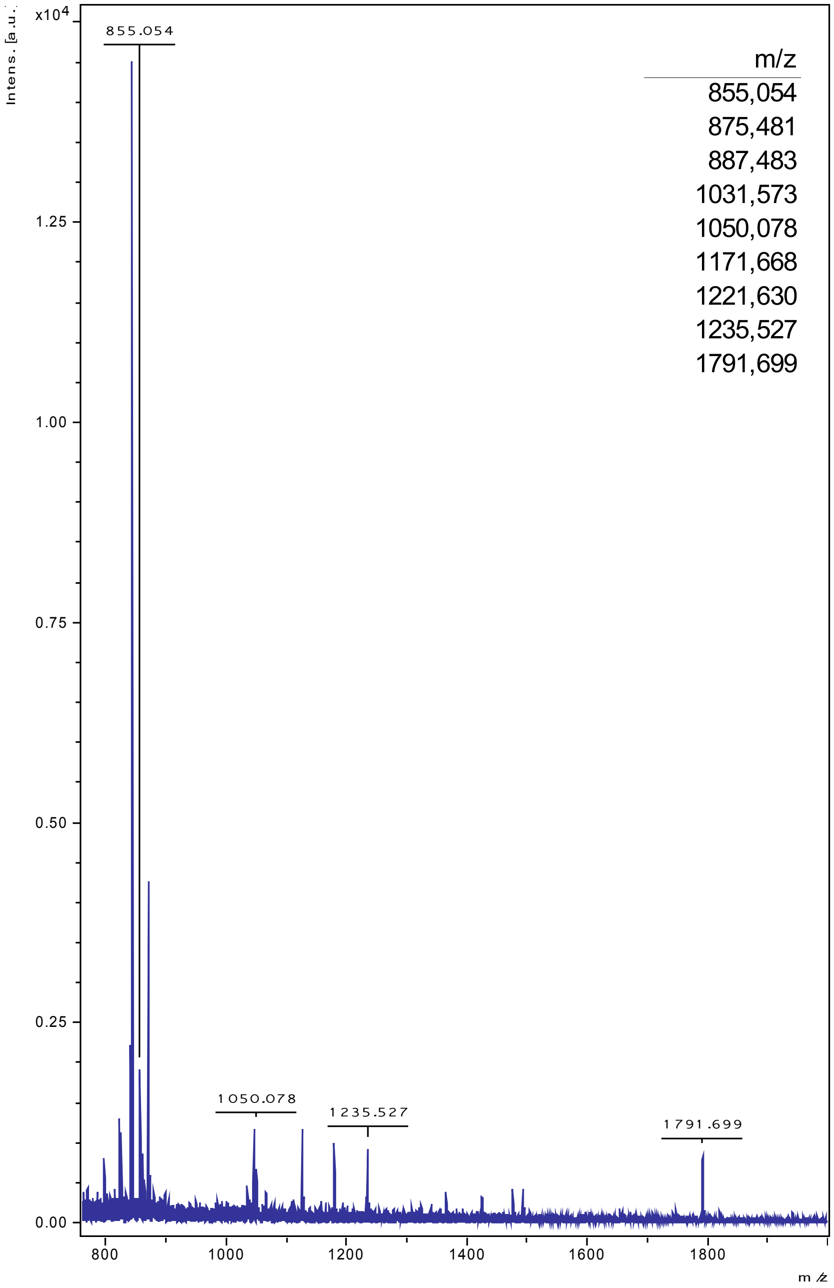

Mascot Search Results

User : irene
Email : isanh@unileon.es
Search title : spot 6400
Database : NCBInr 20100602 (11126451 sequences; 3793134231 residues)
Taxonomy : Streptomyces coelicolor (8575 sequences)
Timestamp : 8 Jun 2010 at 09:03:25 GMT
Top Score : 70 for gi|21223187, succinyl-CoA synthetase subunit alpha [Streptomyces coelicolor A3(2)]

Mascot Score Histogram

Protein score is -10*Log(P), where P is the probability that the observed match is a random event.
Protein scores greater than 52 are significant (p<0.05).

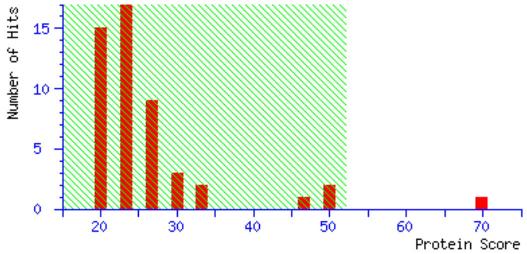

Index

	Accession	Mass	Score	Description
1.	gi|21223187	30277	70	succinyl-CoA synthetase subunit alpha [Streptomyces coelicolor A3(2)]
2.	gi|21220340	22223	51	cob(I)yrinic acid a,c-diamide adenosyltransferase [Streptomyces coelicolor A3(2)]
3.	gi|1532204	38461	48	putative ABC transporter intracellular ATPase subunit BldkD [Streptomyces coelicolor A3(2)]
4.	gi|21223486	38465	45	BldKD, ABC transporter intracellular ATPase subunit [Streptomyces coelicolor A3(2)]
5.	gi|21221650	36991	33	hypothetical protein SCO3215 [Streptomyces coelicolor A3(2)]
6.	gi|21234217	42020	32	putative plasmid partitioning protein, para2 [Streptomyces coelicolor A3(2)]
7.	gi|21223929	32719	31	formamidopyrimidine-DNA glycosylase [Streptomyces coelicolor A3(2)]
8.	gi|21224337	9513	30	hypothetical protein SCO6001 [Streptomyces coelicolor A3(2)]
9.	gi|21222312	10483	30	hypothetical protein SCO3905 [Streptomyces coelicolor A3(2)]
10.	gi|21219532	11954	28	hypothetical protein SCO1015 [Streptomyces coelicolor A3(2)]

Sequence Coverage: 9%

Matched peptides shown in **Bold Red**

```
  1 MAIWLNKDSK VIVQGMTGAT GMKHTKLMLG DGTEVVGGVN PRKAGTSVDF
 51 DGNEVPVFGT VKEAIEKTGA NVSVIFVPEK FTKDAVVEAI DAEIPLAVVI
101 TEGIAVHDSA AFWSYAGKKG NKTRIIGPNC PGIITPGQSN VGIIPGDITK
151 PGRIGLVSKS GTLTYQMMYE LRDIGFSTAV GIGGDPIIGT THIDALAAFE
201 ADPETDLIVM IGEIGGDAEE RAAEYISKNV TKPVVGYVAG FTAPEGKTMG
251 HAGAIVSGSS GTAQAKKEAL EAAGVKVGKT PTETAKLARA ILAG
```

Peptide Mass Fingerprint for spot 6125: SCO4814

m/z
829,514
874,4628
1034,128
1050,594
1127,715
1471,74
1791,644
2225,115
2312,145

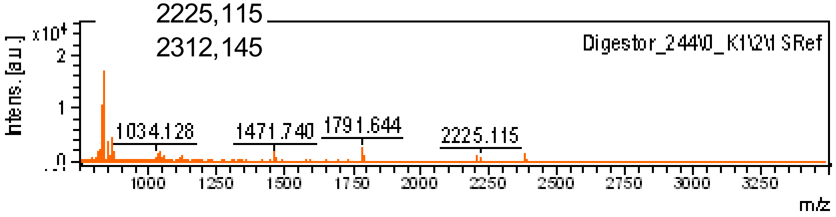

Mascot Search Results

```
User         : irene
Email        : isanh@unileon.es
Search title : spot 6125
Database     : NCBInr 20100602 (11126451 sequences; 3793134231 residues)
Taxonomy     : Streptomyces coelicolor (8575 sequences)
Timestamp    : 9 Jun 2010 at 08:26:31 GMT
Top Score    : 59 for gi|21223192, bifunctional phosphoribosylaminoimidazolecarboxamide formyltransferase/
```

Mascot Score Histogram

Protein score is -10*Log(P), where P is the probability that the observed match is a random event.
Protein scores greater than 52 are significant (p<0.05).

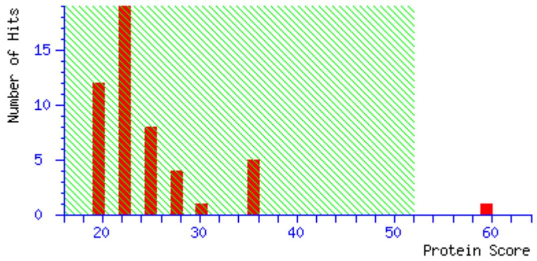

Index

	Accession	Mass	Score	Description
1.	gi|21223192	55351	59	bifunctional phosphoribosylaminoimidazolecarboxamide formyltransferase/
2.	gi|21224356	76616	37	transferase [Streptomyces coelicolor A3(2)]
3.	gi|21220435	34945	36	hypothetical protein SCO1950 [Streptomyces coelicolor A3(2)]
4.	gi|21220402	27034	36	hypothetical protein SCO1915 [Streptomyces coelicolor A3(2)]
5.	gi|256788439	35147	35	hypothetical protein SlivT_28456 [Streptomyces lividans TK24]
6.	gi|21219693	185293	35	hypothetical protein SCO1182 [Streptomyces coelicolor A3(2)]
7.	gi|21220071	49116	31	oxidoreductase membrane protein [Streptomyces coelicolor A3(2)]
8.	gi|21223574	15538	29	hydrogen peroxide sensitive repressor [Streptomyces coelicolor A3(2)]
9.	gi|21221752	12050	29	redoxin [Streptomyces coelicolor A3(2)]
10.	gi|21902159	61416	27	putative integral membrane ATPase [Streptomyces coelicolor A3(2)]

Sequence Coverage: 9%

Matched peptides shown in **Bold Red**

```
  1 MTATAGSNKR AIRRALVSVY DKTGLEDLAR GLHEAGVELV STGSTAGRIA
 51 AAGVPVTKVE ELTGFPECLD GRVKTLHPKV HAGILADLRL ESHRQQLDEL
101 GVAPFDLVVV NLYPFRETVA SGATPDECVE QIDIGGPSMV RAAAKNHPSV
151 AVVTSPARYA DVLLAVEGGG FDLAARKRLA AEAFQHTAAY DVAVASWFAA
201 EYAPVDESGF PDFLGATYER ANTLRYGENP HQPAALYTSP EGGGLAQAEQ
251 LHGKEMSYNN YTDTDAARRA AYDHAEPCVA IIKHANPCGI AIGADVAEAH
301 RKAHDCDPVS AYGGVIAVNR PVSKEMAERV AGIFTEVIVA PDYEDGALEA
351 LTKKKNIRVL RAPAAPAAPV EVKPIDGGAL LQVTDRLQAE GDDPATWTLA
401 TGEALSEAEL AELAFAWRAC RAVKSNAILL AKDGASVGVG MGQVNRVDSA
451 KLAVERAGAE RAQGAYAASD AFFPFPDGLE ILTGAGVKAV VQPGGSVRDE
501 LVVEAAKKAG VTMYFTGTRH FFH
```

Peptide Mass Fingerprint for spot 6167: SCO4837

m/z
866,437
974,514
1048,608
1138,508
1433,727
1470,827
1685,749
1919,983
2227,187

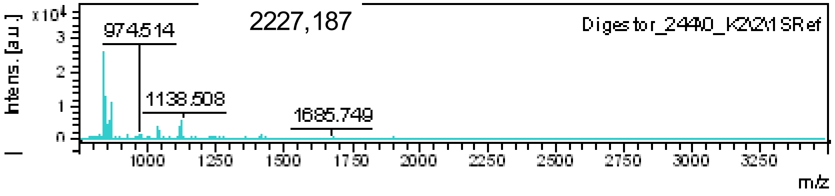

Mascot Search Results

User : irene
Email : isanh@unileon.es
Search title : spot 6167
Database : NCBInr 20100602 (11126451 sequences; 3793134231 residues)
Taxonomy : Streptomyces coelicolor (8575 sequences)
Timestamp : 8 Jun 2010 at 20:08:55 GMT
Top Score : 62 for gi|21223213, serine hydroxymethyltransferase [Streptomyces coelicolor A3(2)]

Mascot Score Histogram

Protein score is -10*Log(P), where P is the probability that the observed match is a random event.
Protein scores greater than 52 are significant (p<0.05).

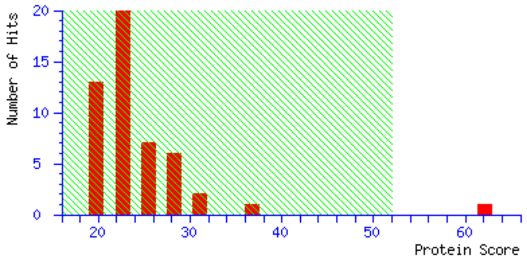

Index

	Accession	Mass	Score	Description
1.	gi|21223213	51573	62	serine hydroxymethyltransferase [Streptomyces coelicolor A3(2)]
2.	gi|21224749	33271	36	hypothetical protein SCO6443 [Streptomyces coelicolor A3(2)]
3.	gi|21223816	42478	30	lipoprotein [Streptomyces coelicolor A3(2)]
4.	gi|21219200	54944	30	glycerol-3-phosphate dehydrogenase [Streptomyces coelicolor A3(2)]
5.	gi|21221968	12371	30	anti-sigma factor antagonist [Streptomyces coelicolor A3(2)]
6.	gi|32141268	54491	30	gamma-aminobutyraldehyde dehydrogenase [Streptomyces coelicolor A3(2)]
7.	gi|21225518	57056	29	oxidoreductase [Streptomyces coelicolor A3(2)]
8.	gi|21221680	64458	28	acyl CoA oxidase [Streptomyces coelicolor A3(2)]
9.	gi|21219807	108469	28	exonuclease [Streptomyces coelicolor A3(2)]
10.	gi|21219211	13484	28	hypothetical protein SCO0682 [Streptomyces coelicolor A3(2)]

Sequence Coverage: 12%

Matched peptides shown in **Bold Red**

1 MPAEISPEST AYRAALDVIR AVEPRVADAI GQEVADQREM LKLIASENYA
51 SPATLLAMGN WFSDK**YAEGT IGR**RFYAGCR NVDTVESLAA EHARELFGAR
101 HAYVQPHSGI DANLVAFWAV LGARVEVPFL EKTGARQVND LTDADWAELR
151 QAFGNQRMLG MSLDAGGHLT HGFRPNISGK MFDQR**SYGTD PATGLIDYEA**
201 **LRASAR**EFKP LIIVAGYSAY PR**LVNFRIMR** EIADEVGATL MVDMAHFAGL
251 VAGKVLTGDF DPVPHAQIVT TTTHKSLRGP RGGMVLCDDS LKDQVDRGCP
301 MVLGGPLPHV MAAKAVALAE AR**RPAFQDYA QRIVDNARAL AEGLTK**RGAT
351 LVTGGTDNHL NLIDVASSYG LTGRQAEAAL LDSGIVTNRN AIPADPNGAW
401 YTSGIRIGTP ALTTRGLGTA EMDEVAGLID RVLTATEPGT TKSGAPSKAS
451 HVLDAKVADE ISHRATDLVA GFPLYPEIDL G

Peptide Mass Fingerprint for spot 6678: SCO4856

m/z
823,377
961,459
1039,542
1117,596
1118,478
1179,557
1223,638
1347,619
1493,611
1765,524
2269,741

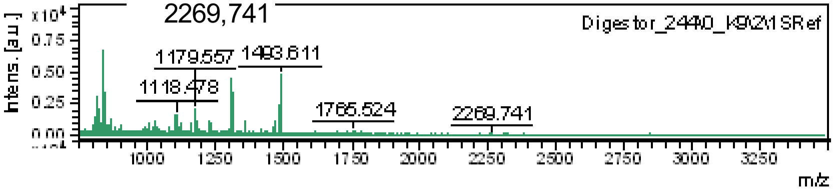

Mascot Search Results

User : irene
Email : isanh@unileon.es
Search title : spot 6678
Database : NCBInr 20100602 (11126451 sequences; 3793134231 residues)
Taxonomy : Streptomyces coelicolor (8575 sequences)
Timestamp : 9 Jun 2010 at 08:51:50 GMT
Top Score : 61 for gi|21223232, succinate dehydrogenase flavoprotein subunit [Streptomyces coelicolor A3(2)]

Mascot Score Histogram

Protein score is -10*Log(P), where P is the probability that the observed match is a random event.
Protein scores greater than 52 are significant (p<0.05).

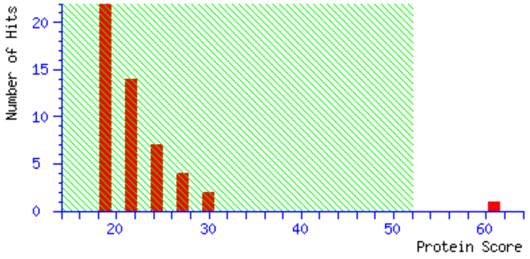

Index

	Accession	Mass	Score	Description
1.	gi|21223232	65033	61	succinate dehydrogenase flavoprotein subunit [Streptomyces coelicolor A3(2)]
2.	gi|31340548	41926	29	two-component system sensor kinase [Streptomyces coelicolor A3(2)]
3.	gi|21234243	42698	29	putative oxidoreductase, mmfh [Streptomyces coelicolor A3(2)]
4.	gi|21223100	6884	28	50S ribosomal protein L30 [Streptomyces coelicolor A3(2)]
5.	gi|21219011	34461	27	AraC family transcription regulator [Streptomyces coelicolor A3(2)]
6.	gi|21223969	7047	27	transcriptional regulator [Streptomyces coelicolor A3(2)]
7.	gi|21221938	52401	26	ATP-binding protein [Streptomyces coelicolor A3(2)]
8.	gi|21223988	14373	25	mutT-like protein [Streptomyces coelicolor A3(2)]
9.	gi|21223146	12216	25	regulatory protein [Streptomyces coelicolor A3(2)]
10.	gi|21223317	32871	25	DNA-binding protein [Streptomyces coelicolor A3(2)]

Sequence Coverage: 8%

Matched peptides shown in **Bold Red**

```
  1 MKVHKYDTVI VGAGGAGMRA AIESTKRSRT AVLTKLYPTR SHTGAAQGGM
 51 AAALANVEED NWEWHTFDTV KGGDYLVDQD AAEILAKEAI DSVLDLEKMG
101 LPFNRTPDGT IDQRRFGGHS RNHGEAPVRR SCYAADRTGH MILQTLYQNC
151 VKEGVEFFNE FYVLDQLITE VDGVKKSAGV VAYELATGEI HVFQAKSVIY
201 ASGGTGKFFK VTSNAHTLTG DGQAAVYRRG LPLEDMEFFQ FHPTGIWRMG
251 ILLTEGARGE GGILRNKDGE RFMEKYAPVM KDLASRDVVS RSIYTEIREG
301 RGCGPEGDHV YLDLTHLPPE QLDAKLPDIT EFARTYLGIE PYTDPIPIQP
351 TAHYAMGGIP TNVEGEVLAD NTTVVPGLYA AGEVACVSVH GANRLGTNSL
401 LDINVFGRRA GIAAAEYAQK ADFVELPEDP ETLVVEQIER LRSSTGTERV
451 ATIRRELQEC MDANVMVFRT EQTIKTAVEK IAELRARYKN VSIQDKGRRF
501 NTDLLEAVEL GNLLDLAEVM AVSALARKES RGGHYREDFP HRDDVNFMRH
551 TMAYREVGDD GAESIRLDYK PVVQTRYQPM ERKY
```

Peptide Mass Fingerprint for spot 6257: SCO4958

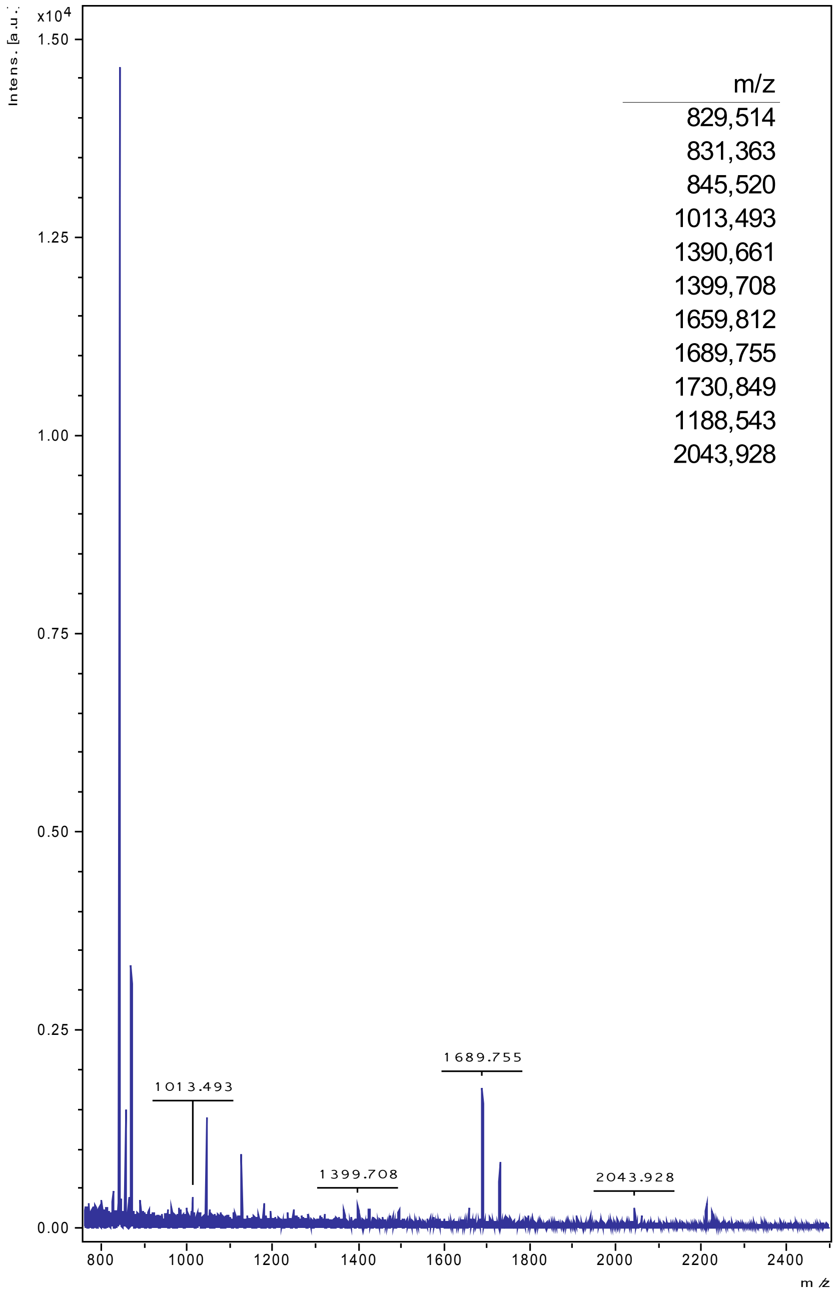

Mascot Search Results

```
User            : irene
Email           : isanh@unileon.es
Search title    : spot 6257
Database        : NCBInr 20100602 (11126451 sequences; 3793134231 residues)
Taxonomy        : Streptomyces coelicolor (8575 sequences)
Timestamp       : 8 Jun 2010 at 08:33:10 GMT
Top Score       : 58 for gi|21223331, cystathionine gamma-synthase [Streptomyces coelicolor A3(2)]
```

Mascot Score Histogram

Protein score is -10*Log(P), where P is the probability that the observed match is a random even
Protein scores greater than 52 are significant (p<0.05).

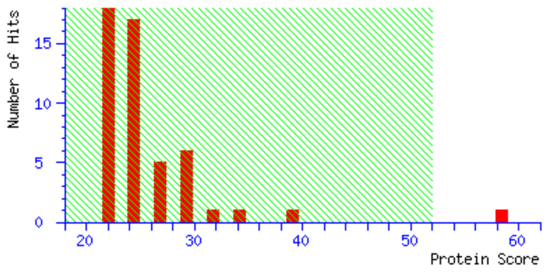

Index

	Accession	Mass	Score	Description
1.	gi|21223331	40844	58	cystathionine gamma-synthase [Streptomyces coelicolor A3(2)]
2.	gi|21222182	56949	40	secreted penicillin binding protein [Streptomyces coelicolor A3(2)]
3.	gi|21218741	68932	35	hypothetical protein SCO0184 [Streptomyces coelicolor A3(2)]
4.	gi|21224977	100528	31	serine/threonine protein kinase [Streptomyces coelicolor A3(2)]
5.	gi|21222513	45928	30	hydroxyglutarate oxidase [Streptomyces coelicolor A3(2)]
6.	gi|13122125	49535	29	putative DNA damage inducible protein [Streptomyces coelicolor A3(2)]
7.	gi|161353682	50783	29	DNA polymerase IV [Streptomyces coelicolor A3(2)]
8.	gi|21220435	34945	28	hypothetical protein SCO1950 [Streptomyces coelicolor A3(2)]
9.	gi|21223401	48275	28	ATP-binding protein [Streptomyces coelicolor A3(2)]
10.	gi|21224785	39246	28	hypothetical protein SCO6481 [Streptomyces coelicolor A3(2)]

Sequence Coverage: **11%**

Matched peptides shown in **Bold Red**

1 MPMSDRHISQ HFETLAIHAG NTADPLTGAV VPPIYQVSTY KQDGVGGLR**G**
51 **GYEYSR**SANP TRTALEENLA ALEGGRRGLA FASGLAAEDC LLRTLLRPGD
101 HVVIPNDAYG GTFRLFAKVA TRWGVEWSVA DTSDAAAVR**A** **ALTPKTK**AVW
151 VETPSNPLLG ITDIAQVAQV ARDAGARLVV DNTFATPYLQ QPLALGADVV
201 VHSLTKYMGG HSDVVGGALI VGDQELGEEL AFHQNAMGAV AGPFDSWLVL
251 R**GTKTLAVRM** **DRHSENATK**V ADMLSRHARV TSVLYPGLPE HPGHEVAAK**Q**
301 **MKAFGGMVSF** **R**VEGGEQAAV EVCNRAKVFT LGESLGGVES LIEHPGRMTH
351 ASAAGSALEV PADLVRLSVG IENADDLLAD LQQALG

Peptide Mass Fingerprint for spot 6340: SCO6282

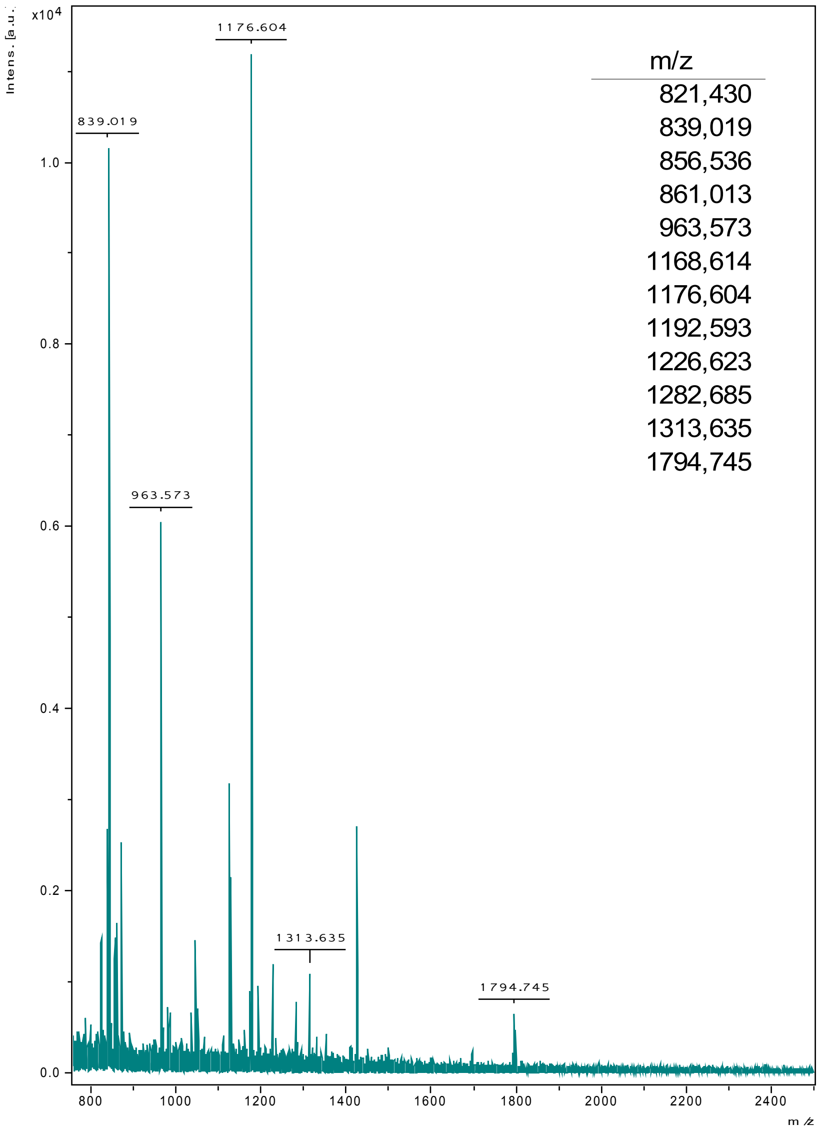

Mascot Search Results

User	: irene
Email	: isanh@unileon.es
Search title	: spot 6340
Database	: NCBInr 20100529 (11111565 sequences; 3786451707 residues)
Taxonomy	: Streptomyces coelicolor (8575 sequences)
Timestamp	: 3 Jun 2010 at 11:12:10 GMT
Top Score	: 92 for gi|21224601, 3-oxoacyl-[acyl-carrier protein] reductase [Streptomyces coelicolor A3(2)]

Mascot Score Histogram

Protein score is -10*Log(P), where P is the probability that the observed match is a random event.
Protein scores greater than 52 are significant (p<0.05).

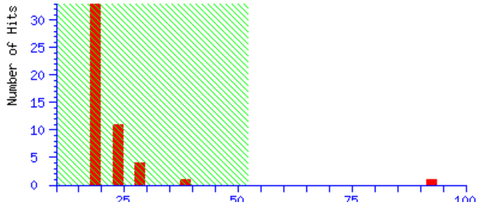

Index

	Accession	Mass	Score	Description
1.	gi|21224601	27414	92	3-oxoacyl-[acyl-carrier protein] reductase [Streptomyces coelicolor A3(2)]
2.	gi|21225778	34325	40	AraC family transcription regulator [Streptomyces coelicolor A3(2)]
3.	gi|21234158	6940	29	putative oxidoreductase subunit [Streptomyces coelicolor A3(2)]
4.	gi|21220174	25825	28	transcriptional regulator [Streptomyces coelicolor A3(2)]
5.	gi|21223851	80012	27	phosphodiesterase [Streptomyces coelicolor A3(2)]
6.	gi|21219610	36798	27	hypothetical protein SCO1095 [Streptomyces coelicolor A3(2)]
7.	gi|21220064	24501	25	acyltransferase [Streptomyces coelicolor A3(2)]
8.	gi|21219588	44969	24	hydrogenase [Streptomyces coelicolor A3(2)]
9.	gi|21225322	13603	24	ABC transporter ATP-binding protein [Streptomyces coelicolor A3(2)]
10.	gi|21219494	12579	24	hypothetical protein SCO0977 [Streptomyces coelicolor A3(2)]

Sequence Coverage: 23%

Matched peptides shown in **Bold Red**

1 MTERLR**SFRM** **TALTNK**TALV TGGSRGIGR**A** **IAQRLGK**EGA LVALTYSSDE
51 AAAK**ETVHSI** **EAAGGR**AFAF RSQLGVPGDA EALWQAFDAQ IGQYADGGSG
101 LDILVNNAGI AGPGLIHEVE EAEYDKVFAV NAK**APFFIIQ** **K**GLERLRDGG
151 RIVNISSGVT K**VAFPGMTSY** **AAAK**GAVEVL TLTLAQTLGS RGITVNAVSP
201 GTIETDIHPW MADPAAK**AHA** **AGFSVFNR**VG QPDDVADVVA FLASDDARWV
251 TGQNIDASGG SGLGL

Peptide Mass Fingerprint for spot 6513: SCO7036

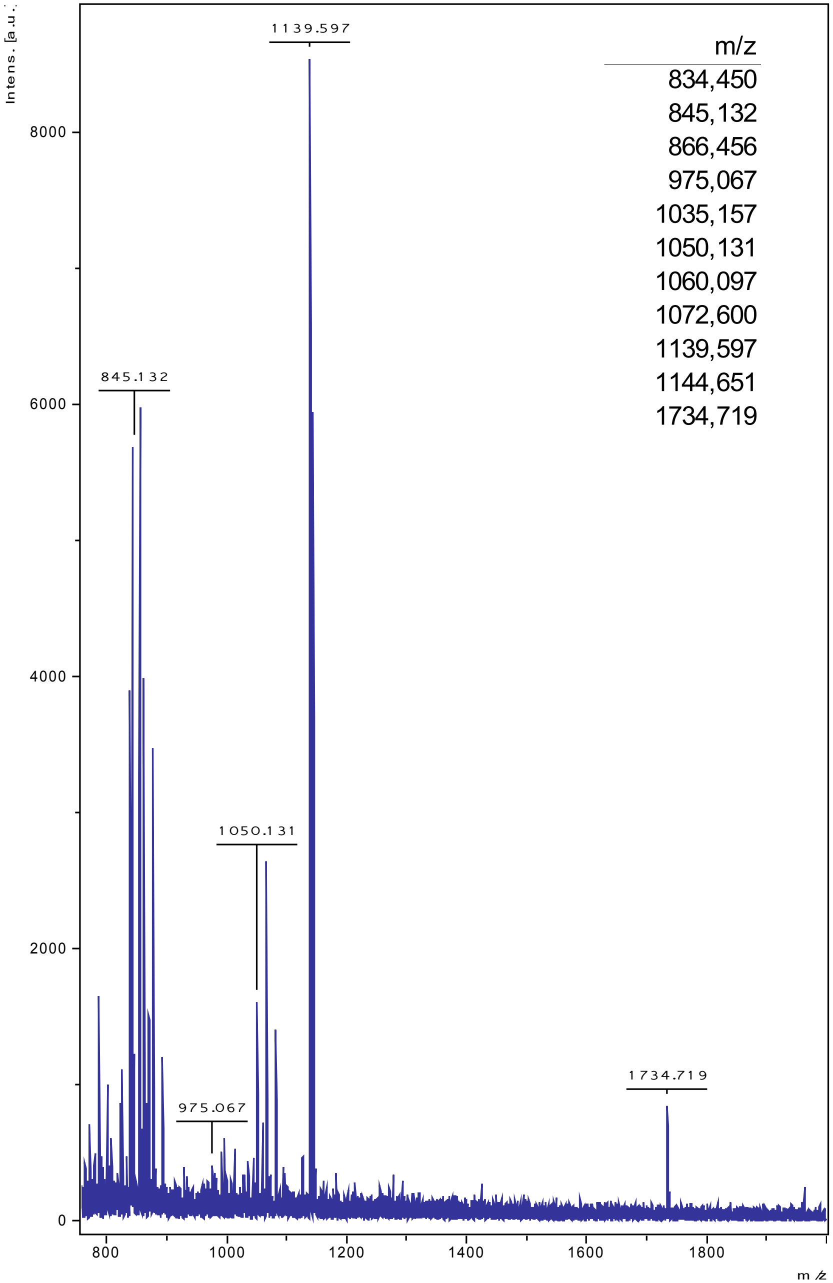

Mascot Search Results

**User**	:	**irene**
**Email**	:	**isanh@unileon.es**
**Search title**	:	**spot 6513**
**Database**	:	**NCBInr 20100602 (11126451 sequences; 3793134231 residues)**
**Taxonomy**	:	**Streptomyces coelicolor (8575 sequences)**
**Timestamp**	:	**8 Jun 2010 at 08:08:12 GMT**
**Top Score**	:	**71 for gi|21225319, argininosuccinate synthase [Streptomyces coelicolor A3(2)]**

Mascot Score Histogram

Protein score is -10*Log(P), where P is the probability that the observed match is a random event.
Protein scores greater than 52 are significant (p<0.05).

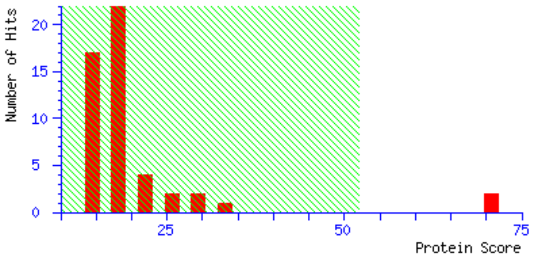

Index

	Accession	Mass	Score	Description
1.	gi|21225319	52408	71	argininosuccinate synthase [Streptomyces coelicolor A3(2)]
2.	gi|216988	53478	69	argininosuccinate synthetase [Streptomyces coelicolor A3(2)]
3.	gi|5459434	2564	34	ADP glucose pyrophosphorylase [Streptomyces coelicolor A3(2)]
4.	gi|21222200	36058	30	integral membrane protein [Streptomyces coelicolor A3(2)]
5.	gi|32141265	32546	28	hypothetical protein SCO5489 [Streptomyces coelicolor A3(2)]
6.	gi|21223200	6504	27	hypothetical protein SCO4823 [Streptomyces coelicolor A3(2)]
7.	gi|21220231	13242	26	hypothetical protein SCO1737 [Streptomyces coelicolor A3(2)]
8.	gi|21224836	60670	23	nitrate reductase beta chain NarH [Streptomyces coelicolor A3(2)]
9.	gi|21219805	20177	23	hypothetical protein SCO1298 [Streptomyces coelicolor A3(2)]
10.	gi|21219747	22767	22	ATP-dependent Clp protease [Streptomyces coelicolor A3(2)]

Sequence Coverage: **12%**

Matched peptides shown in **Bold Red**

1 MSK**VLTSLPT** **GER**VGIAFSG GLDTSVAVAW MRDKGAVPCT YTADIGQYDE
51 PDIASVPDRA K**TYGAEVAR**L VDCRAALVEE GLAALTCGAF HIRSGGR**AYF**
101 **NTTPLGR**AVT GTLLVRAMLE DDVQIWGDGS TFKGNDIERF YR**YGLLANPQ**
151 **LR**IYKPWLDA DFVTELGGRK EMSEWLVAHD LPYRDSTEK**A** **YSTDANIWGA**
201 **THEAK**TLEHL DTGVETVEPI MGVRFWDPSV EIAPEDVTIG FDQGRPVTIN
251 GKEFASAVDL VMEANAVGGR HGMGMSDQIE NRIIEAKSRG IYEAPGMALL
301 HAAYERLVNA IHNEDTLAQY HTEGRRLGRL MYEGRWLDPQ SLMIRESLQR
351 WVGSAVTGEV TLRLRRGEDY SILDTTGPAF SYHPDKLSME RTEDSAFGPV
401 DR**IGQLTMR**N LDIADSRAKL EQYAGLGLIG TANPAIGAAQ AAATGLIGAM
451 PEGGAQAIAS RGEVSADDEL LDRAAMESGT D

Peptide Mass Fingerprint for spot 6771: SCO7510

m/z
1032,536
1060,552
1065,521
1381,696
1424,734
1727,933
1910,828
2383,916

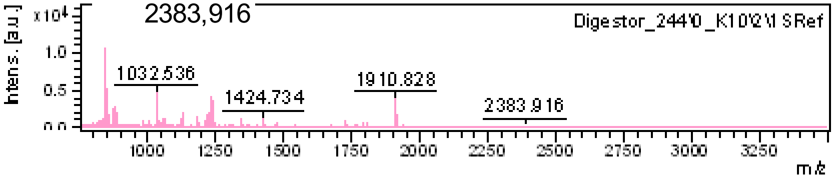

Mascot Search Results

User : irene
Email : isanh@unileon.es
Search title : spot 6771
Database : NCBInr 20100602 (11126451 sequences; 3793134231 residues)
Taxonomy : Streptomyces coelicolor (8575 sequences)
Timestamp : 9 Jun 2010 at 08:02:15 GMT
Top Score : 65 for gi|21225776, peptidyl-prolyl cis-trans isomerase [Streptomyces coelicolor A3(2)]

Mascot Score Histogram

Protein score is -10*Log(P), where P is the probability that the observed match is a random event.
Protein scores greater than 52 are significant (p<0.05).

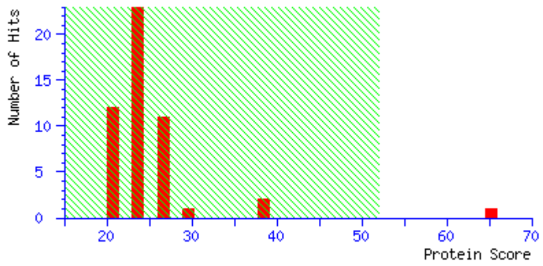

Index

	Accession	Mass	Score	Description
1.	gi|21225776	17878	65	peptidyl-prolyl cis-trans isomerase [Streptomyces coelicolor A3(2)]
2.	gi|32141251	21837	39	TetR family transcriptional regulator [Streptomyces coelicolor A3(2)]
3.	gi|21225765	24077	38	transcriptional regulator [Streptomyces coelicolor A3(2)]
4.	gi|21218759	62886	28	two-component sensor [Streptomyces coelicolor A3(2)]
5.	gi|21224756	17870	27	hypothetical protein SCO6450 [Streptomyces coelicolor A3(2)]
6.	gi|21221455	72760	27	two-component system histidine kinase [Streptomyces coelicolor A3(2)]
7.	gi|21220835	20515	26	hypothetical protein SCO2367 [Streptomyces coelicolor A3(2)]
8.	gi|21219601	22107	26	hypothetical protein SCO1086 [Streptomyces coelicolor A3(2)]
9.	gi|21225981	22061	26	hypothetical protein SCO7722 [Streptomyces coelicolor A3(2)]
10.	gi|21223596	18258	26	acetyltransferase [Streptomyces coelicolor A3(2)]

Sequence Coverage: **18%**

Matched peptides shown in **Bold Red**

```
  1 MSNSKVYFDI DIDGQDAGRI VFELFEDVTP KTARNFLELA TGEHGFGYAG
 51 SPFHRVIPQF MLQGGDFTNG NGTGGKSIYG EKFADENFQL KHTEPYLLSM
101 ANAGPNTNGS QFFVTTVPTP WLDGKHVVFG KVVEGTDVVD KIEGYGSSPA
151 GKTSKEIKVR ASGKL
```

Peptide Mass Fingerprint for spot 6317: SCO7511

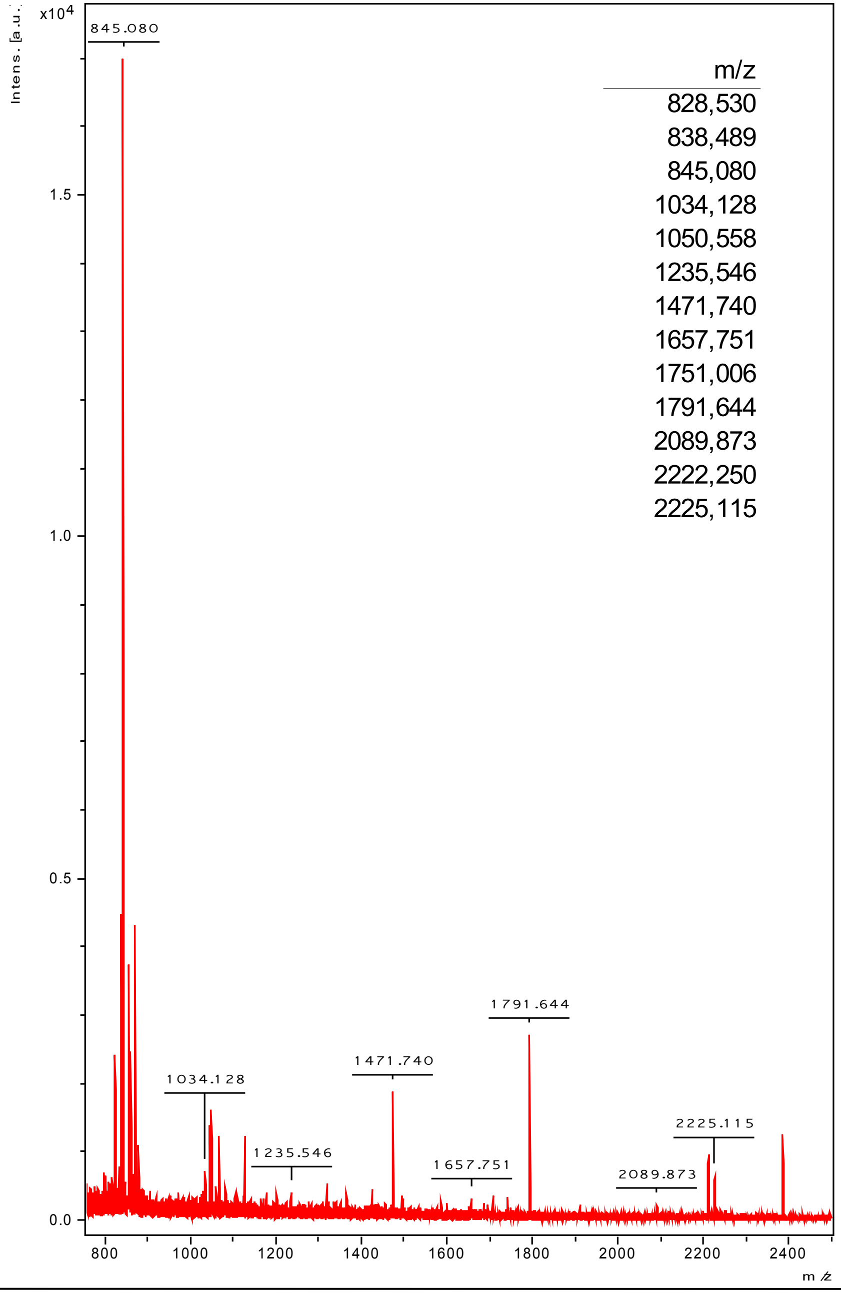

MATRIX SCIENCE Mascot Search Results

**User**	: **irene**
**Email**	: **isanh@unileon.es**
**Search title**	: **spot 6317**
**Database**	: **NCBInr 20100602 (11126451 sequences; 3793134231 residues)**
**Taxonomy**	: **Streptomyces coelicolor (8575 sequences)**
**Timestamp**	: **7 Jun 2010 at 11:44:12 GMT**
**Top Score**	: **60 for gi|21225777, glyceraldehyde 3-phosphate dehydrogenase [Streptomyces coelicolor A3(2)]**

Mascot Score Histogram

Protein score is -10*Log(P), where P is the probability that the observed match is a random event.
Protein scores greater than 52 are significant (p<0.05).

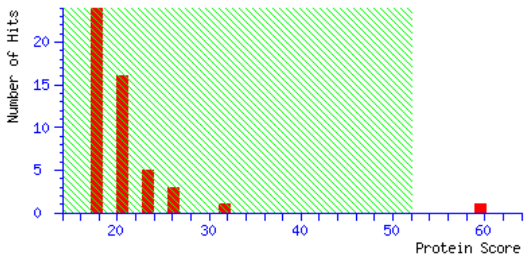

Index

	Accession	Mass	Score	Description
1.	gi|21225777	35003	60	glyceraldehyde 3-phosphate dehydrogenase [Streptomyces coelicolor A3(2)]
2.	gi|21222306	39433	31	hypothetical protein SCO3899 [Streptomyces coelicolor A3(2)]
3.	gi|21234034	10665	27	hypothetical protein SCP1.35 [Streptomyces coelicolor A3(2)]
4.	gi|21223044	12677	27	hypothetical protein SCO4663 [Streptomyces coelicolor A3(2)]
5.	gi|21223028	10468	26	preprotein translocase subunit SecE [Streptomyces coelicolor A3(2)]
6.	gi|21223219	17424	23	integral membrane protein [Streptomyces coelicolor A3(2)]
7.	gi|21219711	21020	23	regulatory protein [Streptomyces coelicolor A3(2)]
8.	gi|21219378	22366	23	TetR family transcriptional regulator [Streptomyces coelicolor A3(2)]
9.	gi|119390540	22225	23	Chain A, Crystal Structure Of Tetr-Family Regulator (Sco0857) From Streptomyces Coelicolor
10.	gi|21221982	71172	23	acetyl-CoA synthetase [Streptomyces coelicolor A3(2)]

Sequence Coverage: **13%**

Matched peptides shown in **Bold Red**

1 MTRIGINGFG RIGRNVLRAL LERDTKLEVV AVNDLTEPAT LAR**LLAFDST**
51 **AGR**LGRPVTV DGDTLVVDGH R**IKVLAER**EP ARLPWAELGV DIVLEATGRF
101 TSADAARAHL TAGARKVLVS APSSGADVTL AYGVNTDAYD PAAHTIVSNA
151 SCTTNALAPL AAVLDELAGI EHGFMTTVHA YTQEQNLQDG PHRDARRARA
201 AGVNIVPTTT GAAKAIGLVL PGLDGKLSGD SIR**VPVPVGS** **IVELNTTVAR**
251 **DVTR**EDVLAA YRAAADGPLA GVLEYSDDPL VSSDITGNPA SSIFDSALTR
301 **VEGRHIK**VVA WYDNEWGFSN RVIDTLALLA TR
